# An observational study of international normalized ratio control according to NICE criteria in patients with non-valvular atrial fibrillation: the SAIL Warfarin Out of Range Descriptors Study (SWORDS)

**DOI:** 10.1093/ehjcvp/pvz071

**Published:** 2019-11-27

**Authors:** Daniel E Harris, Daniel Thayer, Ting Wang, Caroline Brooks, Geoff Murley, Mike Gravenor, Nathan R Hill, Steven Lister, Julian Halcox

**Affiliations:** 1 Swansea University Medical School, Swansea University, Swansea SA28PP, UK; 2 Swansea Bay University Health Board, Morriston Hospital, Swansea SA66NL, UK; 3 HDR UK Wales & Northern Ireland, Data Science Building, Swansea University, Swansea SA28PP, UK; 4 Swansea University Medical School, Data Science Building, Swansea University, Swansea SA28PP, UK; 5 SAIL Databank, Data Science Building, Swansea University, Swansea SA28PP, UK; 6 Department of Outcomes Research, Bristol-Myers Squibb Ltd, Sanderson Rd, Uxbridge UB8 1DH, UK; 7 Department of Health Economics, Bristol-Myers Squibb Ltd, Uxbridge UB8 1DH, UK

**Keywords:** Warfarin, Pharmacoepidemiology, Atrial fibrillation

## Abstract

**Aims:**

In patients with non-valvular atrial fibrillation prescribed warfarin, the UK National Institute of Health and Care Excellence (NICE) defines poor anticoagulation as a time in therapeutic range (TTR) of <65%, any two international normalized ratios (INRs) within a 6-month period of ≤1.5 (‘low’), two INRs ≥5 within 6 months, or any INR ≥8 (‘high’). Our objectives were to (i) quantify the number of patients with poor INR control and (ii) describe the demographic and clinical characteristics associated with poor INR control.

**Method and results:**

Linked anonymized health record data for Wales, UK (2006–2017) was used to evaluate patients prescribed warfarin who had at least 6 months of INR data. 32 380 patients were included. In total, 13 913 (43.0%) patients had at least one of the NICE markers of poor INR control. Importantly, in the 24 123 (74.6%) of the cohort with an acceptable TTR (≥65%), 5676 (23.5%) had either low or high INR readings at some point in their history. In a multivariable regression female gender, age (≥75 years), excess alcohol, diabetes heart failure, ischaemic heart disease, and respiratory disease were independently associated with all markers of poor INR control.

**Conclusion:**

Acceptable INR control according to NICE standards is poor. Of those with an acceptable TTR (>65%), one-quarter still had unacceptably low or high INR levels according to NICE criteria. Thus, only using TTR to assess effectiveness with warfarin has the potential to miss a large number of patients with non-therapeutic INRs who are likely to be at increased risk.

## Introduction

Warfarin is the most common oral anticoagulant prescribed to reduce the risk of stroke in patients with atrial fibrillation (AF). Warfarin, like other vitamin K antagonists (VKAs), has several limitations, including many drug–drug and drug–food interactions.^1^ Furthermore, patient characteristics and comorbidities can lead to high intra- and inter-patient variability in response.^2^^,^^3^ In patients with non-valvular AF (NVAF) without any other indication for anticoagulation, current guidelines recommend an international normalized ratio (INR) range of 2.0–3.0.^4–6^ The net clinical benefit of warfarin is associated with the proportion of time that INR values are maintained within the therapeutic range, referred to as the time in therapeutic range (TTR).^7^^,^^8^ Subtherapeutic INR results are associated with an increase in thromboembolism,^9^ while supertherapeutic INR results are associated with increased risk of bleeding including haemorrhagic stroke. ^10–12^

In the UK, the National Institute for Health and Care Excellence (NICE) recommends that, in patients prescribed warfarin for AF to, ‘Reassess anticoagulation for a person with poor anticoagulation control shown by any of the following: two INR values higher than five or one INR value higher than eight within the past 6 months; two INR values <1.5 within the past 6 months; (and/or ) TTR <65%’.^13^ NICE advises that ‘If anticoagulation control cannot be improved, then the risks and benefits of alternative stroke prevention strategies should be discussed with the patient ’. For patients with NVAF alternative anticoagulation with direct oral anticoagulants (DOACs) can now be provided. ^14^^,^^15^

A number of observational studies and clinical trials have reported the TTR of patients prescribed VKAs for AF,^3^^,^^10^^,^^16–21^ with the average TTR in these studies ranging from 53.7% to 68.4%, highlighting the increased risk of stroke and systemic embolism with subtherapeutic INR, as well as the excess bleeding risk with supertherapeutic INRs. However, the wider variability in INR control described by frequency of very low or very high INRs (as defined by NICE), as distinct from TTR, has not been previously described. This is of particular importance as it would characterize important therapeutic gaps at both an individual and population level, which are not captured by TTR alone.

The objectives of this study were (i) to quantify the number of patients with NVAF prescribed warfarin who exhibit NICE-defined poor INR control and (ii) describe the demographic and clinical characteristics of these patients, as well as the relationship between these characteristics and poor INR control.

## Methods

### Study design and data sources

A retrospective observational cohort study was conducted using linked anonymized healthcare data for patients prescribed warfarin for NVAF between January 2006 and April 2017 in Wales, UK, using the Secure Anonymised Information Linkage (SAIL) Databank.^22–24^ SAIL is part of the national e-health records research infrastructure. The following datasets held within SAIL were linked: the Patient Episode Database for Wales (PEDW), ^25^ which records hospital admission and discharge dates, diagnoses and operational procedures, demographic data, and date of death where applicable for the population of Wales; the Welsh Longitudinal General Practice (WLGP) dataset^26^ containing demographic, clinical, and prescribing data for ∼76% of primary care practices across Wales; the Welsh Demographic dataset, ^27^ which contains basic demographic information and history of individuals’ residence in Wales and registration with GP practices; and the Welsh Index of Multiple Deprivation (WIMD) 2011, ^26^ an area-based deprivation measure.

Study subjects included those who had a diagnosis of AF/atrial flutter recorded in the WLGP dataset at any point prior to or during the study period and who were at least 18 years old at time of diagnosis. Patients were excluded if they had valvular AF (defined as AF in the presence of mitral stenosis, rheumatic mitral valve disease, prior mitral valve surgery, and any metallic prosthetic heart valve), were pregnant during the study period, or had a history of deep vein thrombosis (DVT) or pulmonary embolism (PE). This AF cohort was then restricted to patients who were prescribed warfarin during the study period and had at least 6 months of recurrent INR tests recorded in the WLGP dataset during the study period (excluding the first 6 weeks after start of treatment; a period when the warfarin dose is typically still being tailored to the patient’s needs).

### Medical history, demographic information, and prescriptions

A census date was assigned to each patient from when they met all of the inclusion criteria. Demographic data, prior diagnoses, and comorbidities (chosen to reflect standard stroke and bleeding risk classification, and comorbidities of major organ systems) prior to the census date for each patient were identified. Individual age was calculated at the census date. The presence of heart failure, hypertension, vascular disease [defined as prior myocardial infarction (MI) or peripheral vascular disease (PVD) including peripheral artery disease and aortic plaque], prior stroke [including transient ischaemic attack (TIA)], gender, and age were used to calculate the individual CHA_2_DS_2_-VASc score. In addition, the presence of the following were also identified prior to the census date for each patient (see Supplementary material online, *Table S1* for list codes): chronic kidney disease (CKD) Stage 4+, chronic liver disease (including cirrhosis, fibrosis, chronic hepatitis and chronic active hepatitis, fatty liver, sclerosis of the liver, unspecified alcoholic liver damage, and hepatic failure), dementia, thyroid disease (both hyper and hypothyroidism), epilepsy and respiratory disease, ischaemic heart disease (including stable, unstable, and MI), haemorrhagic stroke, major bleeding events (including respiratory bleeds, urinary tract bleeds, intracranial bleeds, and gastrointestinal bleeds) and excess alcohol consumption.

### Calculation of individual time in therapeutic range and identification of low and high international normalized ratios

NICE recommends using the Rosendaal method for calculating TTR; this method assumes a linear change in INR between consecutive tests (e.g. if two consecutive INR tests are 2.5 and 3.5 with 30 days between tests, the method estimates that 15 days were in range, and 15 days were out of range.^28^^,^^29^ Thus, the estimated TTR is 50% during those 30 days period).

In this study, a modified Rosendaal method was used to calculate individual TTR. Following the census date for each patient, the first 6 weeks of INR results were excluded, to account for any initiation period. Individual INR results were identified, as well as the time span between them; when the interval between INR results was >84 days, the INR test results were excluded from the overall calculation of individual TTR. The calculation began again when there were two INR results within 84 days carried out because long gaps between INR tests most likely represented periods where treatment was discontinued. An INR value of <2.0 was considered subtherapeutic and an INR value >3.0 was considered supertherapeutic.

Patients were categorized as having: (i) ‘unacceptable’ or ‘acceptable’ individual TTR control (< 65% or ≥ 65%, respectively); (ii) ‘low’ INRs (two INR results <1.5 in any 6-month period), or (iii) ‘high’ INRs defined by two INR results >5 in any 6-month period or one result >8. In addition, these three markers were combined into an overall ‘poor’ INR control category, which included all patients with at least one of these NICE-defined indicators of poor control. Patients without any NICE criteria of poor INR control were categorized as ‘adequate’ INR control.

### Statistical methods

Baseline variables and characteristics of patients included in the analysis were presented as percentages or means with standard deviations. Characteristics of patients with each of the three markers of poor control, as well as the overall poor control category, were compared to those with ‘acceptable’ INR control (defined as the absence of any marker of poor control). Differences in these characteristics between groups were summarized using the χ^2^ tests for categorical variables and independent *t*-tests for continuous variables. Next, we investigated two sets of multivariable models for the adverse outcomes. First, a binary logistic regression model was constructed with CHA_2_DS_2_-VASc score and deprivation index (using WIMD quintiles) as the predictors and ‘poor’ control vs ‘adequate’ as the primary (binary) dependent outcome. This model was repeated with ‘unacceptable’ TTR, ‘low’ INR, and ‘high’ INR as the dependent outcome (in each case in a binary comparison with ‘adequate’ INR control).

The second set of models attempted to identify all independent risk factors, by testing all available predictors from the baseline comorbidities and risk factors (including those components within the CHA_2_DS_2_-VASc score), age, gender, and WIMD quintile. Binary dependent variables were the same as above (each of the three individual markers of poor control, as well as the overall poor control category, in comparison with a baseline good control). For this exploratory analysis, a large number of independent variables were considered, and the final set of predictors was chosen based on a search of all models (without interactions) and minimizing the Bayesian Information Criterion (BIC).^30^ Model selection was also carried out using the Lasso regularization method, ^31^ to check for consistency in the variables found in the final models. Analysis was carried out using SPSS version 22.0 and the package glmnet in R.^32^

### Missing data

Comparisons were made between those included in the final cohort for analysis and (i) those with NVAF prescribed warfarin but with <6 months of INR test results for analysis, and (ii) those where there was no INR recorded in the WLGP dataset (see Supplementary material online, *Table S2*). Finally, within the final cohort for analysis, comparisons were made between those with and without deprivation index data available (see Supplementary material online, *Table S3*).

## Results

**Figure 1:**
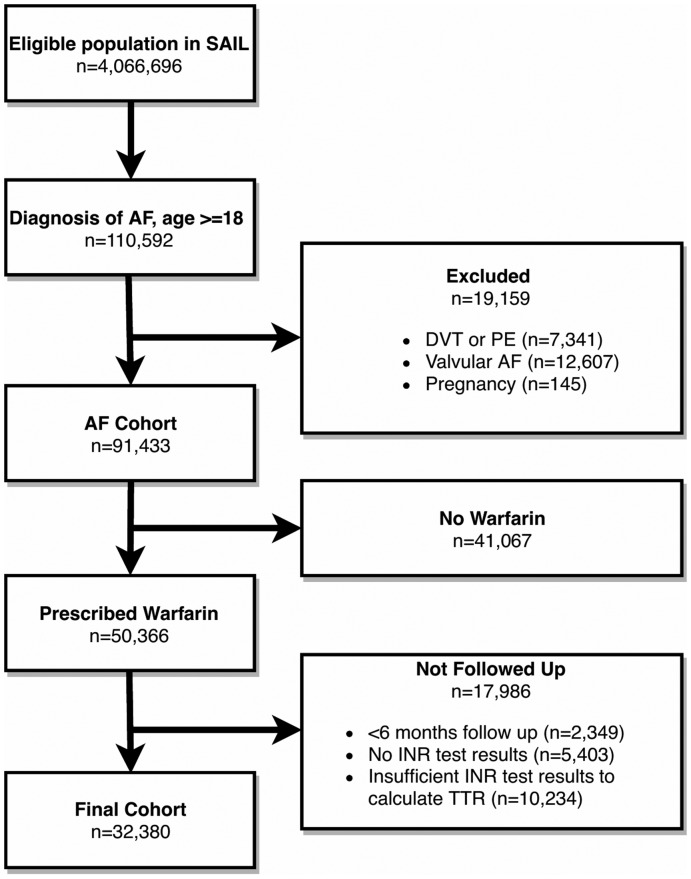
Inclusion criteria for study cohort.

Over 4 million patient records were identified in the SAIL Databank during the study period; 110 592 had a diagnosis of AF and were aged over 18 at the time of diagnosis, of whom a total of 32 380 met the final inclusion criteria for this study (*Figure 1*). During a mean follow-up time of 4.3 years per patient, the mean TTR was 72.6%; 42.5% of the cohort was female; the mean age was 73.5 years (standard deviation = 9.7 years); and the median CHA_2_DS_2_-VASc score was 3 (*Table 1*).

**Table 1:** Cohort demographics and medical history (*N* = 32 380)

	*N* (%)
Age (years)	
<65	5412 (16.7)
65–74	10 875 (33.6)
≥75	16 093 (49.7)
Female	13 751 (42.5)
Deprivation index (quintile)	
1 (most deprived)	5309 (17.5)
2	5875 (19.3)
3	6728 (22.1)
4	5862 (19.3)
5	6645 (21.8)
CHA_2_DS_2_-Vasc score	
0 and 1	4356 (13.5)
2	5949 (18.4)
3	7242 (22.4)
4	6814 (21.0)
5	4281 (13.2)
6	2495 (7.7)
≥7	1243 (3.8)
Excessive alcohol intake	850 (2.6)
Cancer	6134 (18.9)
CKD Stage 4+	375 (1.2)
Dementia	364 (1.1)
Diabetes	6876 (21.2)
Epilepsy	206 (0.6)
Haemorrhagic stroke	204 (0.6)
Heart failure	7264 (22.4)
Hypertension	21 234 (65.6)
Ischaemic heart disease	9641 (29.8)
Ischaemic stroke	6661 (20.6)
Liver disease	611 (1.9)
Major bleeding event	4536 (14.0)
Peripheral vascular disease	1883 (5.8)
Respiratory disease	6305 (19.5)
Thromboembolism	426 (1.3)
Thyroid disease	4079 (12.6)

**Figure 2:**
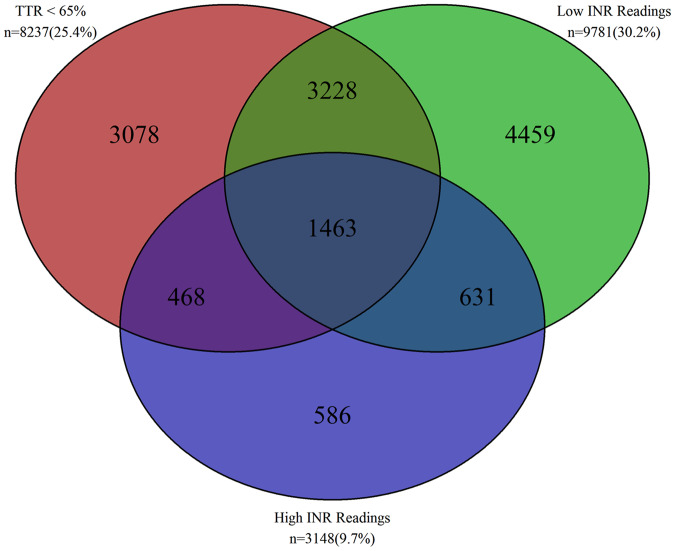
Number of patients with poor international normalized ratio control according to NICE criteria (total cohort = 32 380).

In total, 13 913 (43.0%) patients had at least one of the NICE markers of poor INR control (*Figure 2*). Of this group, 8237 (25.4%) had an unacceptable TTR (<65%) and 9781 (30.2%) had two low -INR readings within a 6-month period. Overall, 3148 (9.7%) had high INRs during the study period, including 2649 (8.2%) that had two or more INR results >5 in a 6-month period, and 961 (3.0%) had an INR of >8.

In the 24 143 (74.6%) cohort with an acceptable TTR (≥65%), many had other signs of poor INR control: 5090 (21.1%) had low INRs and 1217 (5.0%) had high INRs. Overall, of those with acceptable TTR, 5676 (23.5%) had either low or high INR readings at some point in their history.

When considering European Society of Cardiology guidelines, which recommend a TTR ≥70%; 11 876 (36.7%) of patients’ TTR fell below this threshold.^6^ Furthermore, of the 20 504 patients with recommended TTR ≥70%, 3990 (19.5%) patients met the NICE criteria for low or high INRs (Supplementary material online, *Figure S2*).

**Figure 3:**
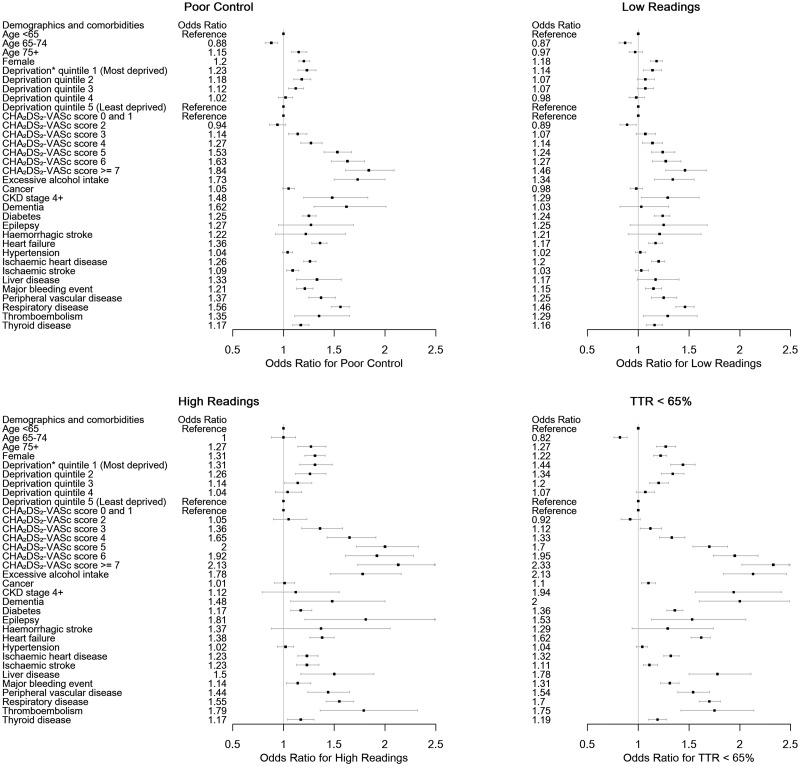
Characteristics associated with poor international normalized ratio control. *Deprivation index used is the WIMD quintile.

**Figure 4:**
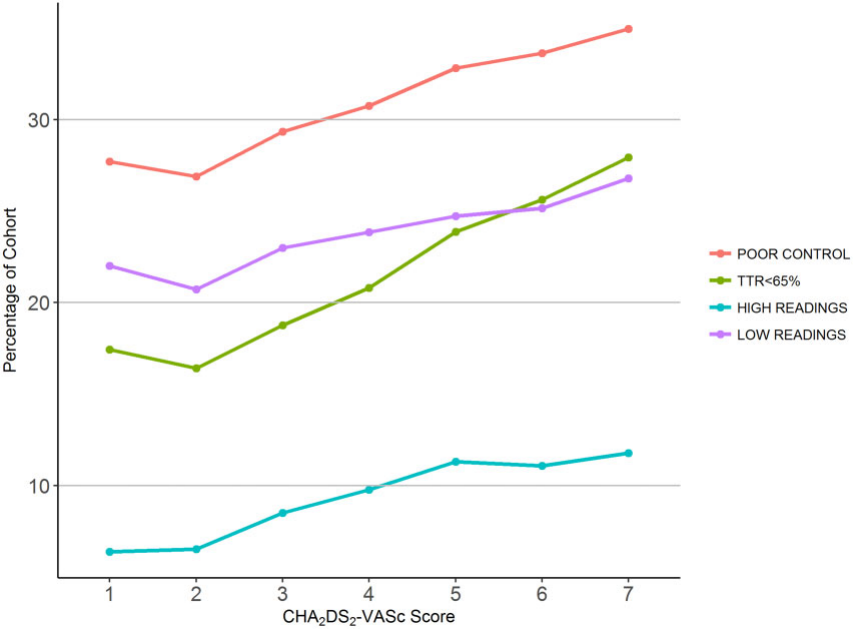
International normalized ratio control vs. thromboembolic risk.

Patient characteristics associated with one or more signs of poor INR control include female sex, increasing social deprivation, increasing CHA_2_DS_2_-VASc score, heart failure, prior bleeding events, cancer, ischaemic heart disease, PVD, ischaemic stroke, thromboembolism, thyroid disease, respiratory disease, diabetes, epilepsy, dementia, excessive alcohol intake, liver disease, and CKD Stage 4+ (*Figure 3*). Increasing CHA_2_DS_2_-VASc score from 2 was associated with an increasing likelihood of each marker of poor INR control, including the overall combined marker of poor INR control (*Figure 4*).

### Multivariable modelling

In the first set of models, a CHA_2_DS_2_-VASc score of 3 or more was significantly associated with all markers of poor INR control (*Table 2*). A similar relationship was observed between higher levels of deprivation and the risk of poor INR control.

**Table 2:** Multivariable logistic regression model of international normalized ratio control vs. deprivation index^a^ and CHA_2_DS_2_-VASc score

Predictor	Poor control	Low INRs	High INRs	TTR <65%
Deprivation index^a^ (quintiles). Adjusted odds ratio (95% CI)				
5 (least deprived)	Reference, overall *P*-value (<0.001)	Reference, overall *P*-value (<0.001)	Reference, overall *P*-value (<0.001)	Reference, overall *P*-value (<0.001)
4	1.02 (0.95–1.10)	0.99 (0.92–1.08)	1.05 (0.93–1.20)	1.07 (0.98–1.17)
3	1.12 (1.05–1.21)	1.11 (1.02–1.19)	1.18 (1.05–1.33)	1.21 (1.12–1.32)
2	1.17 (1.09–1.26)	1.12 (1.03–1.21)	1.31 (1.16–1.48)	1.32 (1.22–1.44)
1 (most deprived)	1.21 (1.13–1.31)	1.18 (1.09–1.28)	1.36 (1.20–1.54)	1.41 (1.30–1.54)
CHA_2_DS_2_-VASc score. Adjusted odds ratio (95% CI)				
0 or 1	Reference, overall *P*-value (<0.001)	Reference, overall *P*-value (<0.001)	Reference, overall *P*-value (<0.001)	Reference, overall *P*-value (<0.001)
2	0.94 (0.86–1.02)	0.90 (0.82–0.98)	1.02 (0.87–1.19)	0.91 (0.82–1.01)
3	1.13 (1.05–1.23)	1.11 (1.01–1.21)	1.39 (1.19–1.61)	1.14 (1.04–1.26)
4	1.27 (1.17–1.37)	1.20 (1.10–1.32)	1.72 (1.49–2.01)	1.36 (1.23–1.50)
5	1.53 (1.39–1.67)	1.39 (1.26–1.53)	2.21 (1.85–2.65)	1.76 (1.58–1.95)
6	1.62 (1.46–1.79)	1.46 (1.30–1.63)	2.22 (1.90–2.59)	1.99 (1.77–2.25)
≥7	1.82 (1.60–2.07)	1.69 (1.46–1.96)	2.57 (2.07–3.20)	2.37 (2.04–2.75)

a Deprivation index used is the WIMD quintile.^26^

In the second set of models, exploring all possible independent variables, after BIC model selection, age, female gender, excess alcohol consumption, heart failure, ischaemic heart disease, respiratory disease, and diabetes were independently associated with all measures of poor INR control (*Table 3*). Peripheral vascular disease was associated with ‘poor control’, ‘high INRs’, and ‘TTR <65%’, while prior major bleeding and dementia were associated with ‘poor control’ and ‘TTR < 65%’. Ischaemic stroke was only associated with ‘high INRs’ and deprivation index was only associated with ‘TTR < 65%’. Highest adjusted odds ratios, across all markers or poor control, were detected for excess alcohol consumption, which is also predictive of bleeding.

**Table 3:** Multivariable regression models of patient characteristics vs. international normalized ratio control^a^

	Poor control	Low INRs	High INRs	TTR <65%
	Adjusted odds ratio (95% CI), *P*-value	Adjusted odds ratio (95% CI), *P*-value	Adjusted odds ratio (95% CI), *P*-value	Adjusted odds ratio (95% CI), *P*-value
Age (years)				
≤64	Reference, <0.001	Reference, <0.001	Reference, <0.001	Reference, <0.001
65–74	0.84 (0.78–0.90)	0.82 (0.76–0.88)	0.88 (0.78–1.01)	0.76 (0.70–0.84)
≥75	1.07 (1.00–1.15)	0.99 (0.92–1.06)	1.19 (1.06–1.35)	1.19 (1.09–1.28)
Female	1.23 (1.17–1.29), <0.001	1.25 (1.19–1.32), <0.001	1.45 (1.33–1.57), <0.001	1.29 (1.21–1.36), <0.001
Excess alcohol	1.79 (1.55–2.08), <0.001	1.62 (1.38–1.90), <0.001	2.45 (1.97–3.03), <0.001	2.32 (1.97–2.72), 0.001
Major bleeding events	1.15 (1.08–1.23), 0.001			1.23 (1.14–1.32), <0.001
Cancer				
CKD Stage 4+				
Dementia	1.51 (1.22–1.89), 0.001			1.83 (1.44–2.33), <0.001
Diabetes	1.20 (1.14–1.28), <0.001	1.24 (1.17–1.32), <0.001	1.21 (1.10–1.33), <0.001	1.29 (1.21–1.38), 0.001
Epilepsy				
Heart failure	1.24 (1.17–1.31), <0.001	1.17 (1.11–1.25), <0.001	1.39 (1.27–1.53), <0.001	1.42 (1.33–1.52), <0.001
Hypertension				
Ischaemic heart disease	1.17 (1.11–1.23), <0.001	1.20 (1.14–1.27), <0.001	1.22 (1.11–1.32), <0.001	1.20 (1.13–1.27), <0.001
Ischaemic stroke			1.24 (1.13–1.36), 0.001	
Liver disease				
PVD	1.25 (1.13–1.38), <0.001		1.42 (1.22–1.65), <0.001	1.35 (1.20–1.51), <0.001
Respiratory disease	1.51 (1.43–1.60), <0.001	1.54 (1.45–1.64), <0.001	1.75 (1.59–1.92), <0.001	1.69 (1.59–1.82), <0.001
Thromboembolis thyroid disease				
Deprivation index^b^ (quintiles)				
5 (Least deprived)				Reference, <0.001
4				1.03 (0.95–1.13)
3				1.15 (1.06–1.26)
2				1.23 (1.13–1.35)
1 (most deprived)				1.28 (1.17–1.40)

a All patient characteristics shown in *Table 1* were modelled, only characteristics that were significant in any of models are shown in the table.

b Deprivation index used is the WIMD quintile.^26^

We found very good match between the variables selected by the BIC and Lasso model selection procedures (classifying by inclusion/exclusion the match was 78.9% for ‘poor control’, 89.4% for ‘Low’, 89.4% for ‘High’, 89.4% for ‘TTR < 65%’; see Supplementary material online, *Table S4*). All predictors highlighted above were consistently selected by both procedures. Bayesian Information Criterion selection tended to be more conservative, with slightly fewer variables selected in the final models.

## Discussion

This is the first population study examining the effectiveness of warfarin therapy according to the NICE clinical guideline criteria for INR indicators of poor anticoagulation control, across a population with NVAF. In this study, only 57.0% of patients had adequate INR control according to NICE criteria. Increasing stroke risk, as assessed by the CHA_2_DS_2_-VASc score, was associated with a greater risk of poor INR control, as were many individual clinical characteristics that are also associated with increased risk of stroke or bleeding. Unlike previous studies, not only was TTR evaluated but also the NICE criteria for unacceptably low and high INR levels. Importantly, we found that almost a quarter of those patients with acceptable TTR (>65%) demonstrated evidence of unacceptably low or high INR levels according to NICE criteria during the study period. These findings suggest that the risk of stroke, systemic embolism and/or bleeding, at both an individual patient and a population level, may be under-appreciated if TTR is followed as the sole measure of effectiveness of anticoagulation. Whilst it is important to recognize that the specific relationships between NICE low and high criteria and risks of major bleeding and stroke have not been definitively characterized, these are pragmatic values identifying very low and high INR readings in chronically treated patients, defined by an expert consensus panel that should mandate clinical attention in UK practice.

This study evaluated the impact of multiple clinical and demographic factors in one of the largest real-world studies of INR control in patients with NVAF. Increasing CHA_2_DS_2_-VASc score (above 3), and hence increasing stroke risk, was strongly associated with poor INR control. As these patients are at the greatest thromboembolic risk, and therefore, likely to derive the greatest absolute benefits from effective anticoagulation, our data emphasize the particular importance of close monitoring and appropriate treatment selection in these vulnerable individuals. Individual risk factors for stroke including diabetes, heart failure, PVD, ischaemic heart disease, and female gender were independently associated with markers of poor INR control. Prior to major bleeding events and excess alcohol consumption, both risk factors for bleeding, were also associated with poor INR control. This is likely to reflect that patients with increasing comorbidity have an increasing number of potential influences on warfarin bioavailability and coagulation factor synthesis.

Previous studies have demonstrated that increasing CHA_2_DS_2_-VASc score, as well as comorbidities including heart failure, diabetes, CKD, chronic obstructive pulmonary disease, female sex, and lower income, are associated with lower TTR. ^2^^,^^3^^,^^21^ The models presented in our study confirm this finding and also show that both CHA_2_DS_2_-VASc score and multiple individual comorbidities are associated with low and high INRs. It is not known whether it is the direct physiological effect of these comorbidities, or medications prescribed for them that are responsible for poor INR control; however, the observed association between increasing stroke risk and risk factors for bleeding associated with poor INR control warrants increased vigilance in those patients with increasing risk of stroke or bleeding.

The mean TTR of our cohort was higher than recorded in many previous studies, and the number of patients achieving adequate TTR had improved each year during the study (Supplementary material online, *Figure S1*). Previous studies have suggested that INR management within anticoagulation clinics is associated with better TTR control.^3^^,^^33^ This study does not make comparisons between individual anticoagulation services or models of service delivery. There are several ways of delivering anticoagulation services in Wales, with many anticoagulation services being provided within primary care GP services. This may have contributed to the high TTR observed in this study, because it is also possible that patients who are difficult to control are managed within specialist anticoagulation services within secondary care, and their data were not included in this study. Furthermore, those with troublesome INR control may have been switched to DOACs, a newer class of medications that were introduced in the latter period of this study.

The observation that the number of patients with adequate TTR increased across the study period, yet the number with low or high INRs remained relatively constant, is of interest but unexplained. It may be possible to improve TTR across the population through improved monitoring, interventions or patient selection, but less easy to prevent low or high INRs in response to acute events or changes to medication, especially in patients with multiple comorbidities that impact on INR variability.^34–36^

We excluded AF patients with ‘valvular AF’ (mitral stenosis, rheumatic mitral valve disease, prior mitral valve surgery, or any metallic prosthetic heart valve), those with a history of DVT or PE and those pregnant during the study period. These patients may have had ‘individualized’ INR targets, which would not necessarily have been identifiable in the SAIL databank and may potentially have biased the study towards a greater number of patients with ‘poor INR control’ when applying specific NICE and/or ESC criteria for AF. Thus, we decided to take a conservative approach by excluding them from the analyses. Furthermore, our clinical experience suggests that these more complex patients are more often managed via specialist secondary care haematology led anticoagulation services and their INR results would not have been available for analysis in this study.

### Strengths and limitations of this study

This is the first study that has investigated not just only TTR but also low and high INR events, as markers of poor therapeutic control with warfarin therapy, allowing us to highlight that there is a substantial cohort of patients likely to be at risk of poor outcomes who may be missed if TTR is the sole focus.

The use of a large, data-rich, linked population data source is a particular strength of this study. The linked primary and secondary care data held by SAIL enable the investigation of a very large cohort of individuals longitudinally over a period of years and across multiple data sources, giving a much more complete picture of patient treatment, health, and characteristics than previous studies.

In calculating the TTR, NICE guidance recommends excluding the first 6 weeks of INR results and calculating the TTR over a maintenance period of 6 months. This recommendation was incorporated in to the methodology of this study. During the study period, it is possible that there were temporary discontinuations of warfarin therapy due to acute illness, in response to elevated INR results or admissions to hospital. In order to address this, periods, where there was a gap of >84 days between INR readings, were excluded, but this is an imperfect measure, and it is not possible to definitively identify gaps in treatment.

Although it may be argued that periods of temporary discontinuation should be excluded from assessing INR control according to NICE criteria, unless patients receive alternative treatment to reduce the risk of stroke, they are exposed to an increased risk of thromboembolic events. The destabilization of INR control during acute illnesses and the prolonged subtherapeutic or supertherapeutic coagulation during gaps in anticoagulation is a recognized limitation in the use of warfarin.

In total, there were 17 905 patients identified with NVAF and prescribed warfarin that were excluded from the study, of which 5368 did not have any INR readings available, and a further 10 190 that had insufficient INR readings (<6 months) to analyse. It is not known why 5368 patients did not have INR readings recorded but it is possible that these patients were managed via coagulation clinics outside of the primary care setting and their results are not incorporated into the WLGP dataset. It is not known what effect the incorporation of their results into this study would have made; however, these patients had a significantly higher rate of nearly all comorbidities and higher prevalence of excess alcohol consumption that were associated with greater likelihood of poor INR control in the models presented in this study.

In the final cohort, 1959 (6.1%) had a missing deprivation index and were, therefore, excluded from the multivariable analyses. This group had slightly lower prevalence of comorbidities (other than excess alcohol consumption), suggesting an overall lower risk group than those included in the multivariable analyses. Regardless, all major comorbidities were well represented in the multivariable models and the inclusion of this group would not be expected to have a significant impact on the observed associations.

Some patients may have had different, individualized INR targets, which would not be evaluable in this study. By identifying and excluding patients with valvular AF and those with other indications for anticoagulation, both groups with potentially higher INR targets, we have limited overestimates of poor INR control. The linkage of hospital and GP datasets has further improved the identification and exclusion of patients. However, it remains possible that undocumented valvular disease, multiple DVTs or PEs, or individually adjusted INR targets, may have resulted in the inclusion of patients with a targeted INR range outside of 2 –3, who would then potentially be misclassified as having poor INR control.

Due to the nature of the study, it was not possible to detect whether excess alcohol consumption has an interacting effect on warfarin, directly affected the INR, or was a marker of poor compliance.

## Conclusion

In this study, 43% of patients had at least one marker of poor INR control. Of those, with an acceptable TTR (>65%) one-quarter still had unacceptably low or high INR levels according to NICE criteria. Paradoxically, patients at the highest risk of stroke and with risk factors for bleeding were most likely to have poor INR control and may benefit from closer attention to therapeutic effectiveness and alternative anticoagulation strategies where appropriate. If TTR is used as the sole measure of warfarin’s therapeutic effectiveness, the risk of stroke, systemic embolism and bleeding may well be under-estimated. Further work is required to define the specific level of risk associated with NICE and other guidelines’ criteria for poor INR control and seek to identify novel measures of INR control for optimal risk stratification.

While the results of this study suggest there is considerable opportunity to improve both embolic and bleeding risk, the relationship between poor INR control and these clinical outcomes remains to be determined. Nevertheless, in accordance with NICE guidelines, almost a half of NVAF patients prescribed warfarin for thromboembolic risk reduction warrant review to optimize INR control or consider alternative anticoagulation strategies where appropriate.

## Approvals 

Research ethics approval was not required for this study. However, permission to conduct this study (study number 646) was granted by the independent Information Governance Review Panel (IGRP) for the SAIL Databank. The IGRP contains independent members from the National Research Ethical Committee and the British Medical Association, as well as lay members of the public. The review process confirms the value of the study, is not service evaluation, and conforms to all current information governance standards.

## Supplementary material

Supplementary material is available at *European Heart Journal – Cardiovascular Pharmacotherapy* online.

## Supplementary Material

pvz071_Supplementary_Data
