# Supplementary material for: An observational study of international normalized ratio control according to NICE criteria in patients with non-valvular atrial fibrillation: the SAIL Warfarin Out of Range Descriptors Study (SWORDS)
Source: Eur Heart J Cardiovasc Pharmacother. 2019 Nov 27;7(1):40–9. doi: 10.1093/ehjcvp/pvz071 (PMC7811400; doi:10.1093/ehjcvp/pvz071)
Supplement: pvz071_Supplementary_Data [file pvz071_supplementary_data.zip › Suppl_table1_05022019.docx]

Supplementary table 1. ICD-10, OPCS and Read codes used to define comorbidities and exclusion criteria

| ICD-10 cods used to define co-morbidities | | |
| --- | --- | --- |
| NAME | CODE | DESCRIPTION |
| DVT/PE | I269 | Pulmonary embolism without mention of acute cor pulmonale |
| DVT/PE | I802 | Phlebitis and thrombophlebitis of other deep vessels of lower extremities |
| DVT/PE | I260 | Pulmonary embolism with mention of acute cor pulmonale |
| PVD | I738 | Other specified peripheral vascular diseases |
| PVD | I739 | Peripheral vascular disease, unspecified |
| PVD | I702 | Atherosclerosis of arteries of extremities |
| PVD | I700 | Atherosclerosis of aorta |
| Cancer | C089 | Malignant neoplasm: Major salivary gland, unspecified |
| Cancer | C48 | Malignant neoplasm of retroperitoneum and peritoneum |
| Cancer | C822 | Follicular lymphoma grade III, unspecified |
| Cancer | C845 | Other and unspecified T-cell lymphomas |
| Cancer | C31 | Malignant neoplasm of accessory sinuses |
| Cancer | C187 | Malignant neoplasm: Sigmoid colon |
| Cancer | C939 | Monocytic leukaemia, unspecified |
| Cancer | C173 | Malignant neoplasm: Meckel's diverticulum |
| Cancer | C06 | Malignant neoplasm of other and unspecified parts of mouth |
| Cancer | C838 | Other types of diffuse non-Hodgkin's lymphoma |
| Cancer | C509 | Malignant neoplasm: Breast, unspecified |
| Cancer | C169 | Malignant neoplasm: Stomach, unspecified |
| Cancer | C068 | Malignant neoplasm: Overlapping lesion of other and unspecified parts of mouth |
| Cancer | C880 | [NULL] |
| Cancer | C052 | Malignant neoplasm: Uvula |
| Cancer | C67 | Malignant neoplasm of bladder |
| Cancer | C693 | Malignant neoplasm: Choroid |
| Cancer | C694 | Malignant neoplasm: Ciliary body |
| Cancer | C321 | Malignant neoplasm: Supraglottis |
| Cancer | C910 | Acute lymphoblastic leukaemia [ALL] |
| Cancer | C178 | Malignant neoplasm: Overlapping lesion of small intestine |
| Cancer | C062 | Malignant neoplasm: Retromolar area |
| Cancer | C81 | Hodgkin lymphoma |
| Cancer | C388 | Malignant neoplasm: Overlapping lesion of heart, mediastinum and pleura |
| Cancer | C049 | Malignant neoplasm: Floor of mouth, unspecified |
| Cancer | C920 | Acute myeloid leukaemia |
| Cancer | C05 | Malignant neoplasm of palate |
| Cancer | C313 | Malignant neoplasm: Sphenoidal sinus |
| Cancer | C467 | Kaposi sarcoma of other sites |
| Cancer | C462 | Kaposi sarcoma of palate |
| Cancer | C468 | Kaposi sarcoma of multiple organs |
| Cancer | C102 | Malignant neoplasm: Lateral wall of oropharynx |
| Cancer | C749 | Malignant neoplasm: Adrenal gland, unspecified |
| Cancer | C845 | Other mature T/NK-cell lymphomas |
| Cancer | C142 | Malignant neoplasm: Waldeyer's ring |
| Cancer | C97X | Malignant neoplasms of independent (primary) multiple sites |
| Cancer | C811 | Nodular sclerosis classical Hodgkin lymphoma |
| Cancer | C812 | Mixed cellularity classical Hodgkin lymphoma |
| Cancer | C68 | Malignant neoplasm of other and unspecified urinary organs |
| Cancer | C150 | Malignant neoplasm: Cervical part of oesophagus |
| Cancer | C921 | Chronic myeloid leukaemia [CML], BCR/ABL-positive |
| Cancer | C418 | Malignant neoplasm: Overlapping lesion of bone and articular cartilage |
| Cancer | C101 | Malignant neoplasm: Anterior surface of epiglottis |
| Cancer | C679 | Malignant neoplasm: Bladder, unspecified |
| Cancer | C84 | Mature T/NK-cell lymphomas |
| Cancer | C837 | Burkitt lymphoma |
| Cancer | C914 | Hairy-cell leukaemia |
| Cancer | C718 | Malignant neoplasm: Overlapping lesion of brain |
| Cancer | C383 | Malignant neoplasm: Mediastinum, part unspecified |
| Cancer | C548 | Malignant neoplasm: Overlapping lesion of corpus uteri |
| Cancer | C91 | Lymphoid leukaemia |
| Cancer | C171 | Malignant neoplasm: Jejunum |
| Cancer | C924 | Acute promyelocytic leukaemia |
| Cancer | C180 | Malignant neoplasm: Caecum |
| Cancer | C724 | Malignant neoplasm: Acoustic nerve |
| Cancer | C15 | Malignant neoplasm of oesophagus |
| Cancer | C530 | Malignant neoplasm: Endocervix |
| Cancer | C830 | Small cell B-cell lymphoma |
| Cancer | C95 | Leukaemia of unspecified cell type |
| Cancer | C61X | Malignant neoplasm of prostate |
| Cancer | C716 | Malignant neoplasm: Cerebellum |
| Cancer | C721 | Malignant neoplasm: Cauda equina |
| Cancer | C71 | Malignant neoplasm of brain |
| Cancer | C43 | Malignant melanoma of skin |
| Cancer | C829 | Follicular lymphoma, unspecified |
| Cancer | C600 | Malignant neoplasm: Prepuce |
| Cancer | C763 | Malignant neoplasm of other and ill-defined sites: Pelvis |
| Cancer | C937 | Other monocytic leukaemia |
| Cancer | C838 | Other non-follicular lymphoma |
| Cancer | C813 | Hodgkin's disease: Lymphocytic depletion |
| Cancer | C103 | Malignant neoplasm: Posterior wall of oropharynx |
| Cancer | C81 | Hodgkin's disease |
| Cancer | C542 | Malignant neoplasm: Myometrium |
| Cancer | C501 | Malignant neoplasm: Central portion of breast |
| Cancer | C218 | Malignant neoplasm: Overlapping lesion of rectum, anus and anal canal |
| Cancer | C574 | Malignant neoplasm: Uterine adnexa, unspecified |
| Cancer | C050 | Malignant neoplasm: Hard palate |
| Cancer | C930 | Acute monoblastic/monocytic leukaemia |
| Cancer | C470 | Malignant neoplasm: Peripheral nerves of head, face and neck |
| Cancer | C915 | Adult T-cell lymphoma/leukaemia (HTLV-1-associated) |
| Cancer | C251 | Malignant neoplasm: Body of pancreas |
| Cancer | C459 | Mesothelioma, unspecified |
| Cancer | C827 | Other types of follicular lymphoma |
| Cancer | C414 | Malignant neoplasm: Pelvic bones, sacrum and coccyx |
| Cancer | C220 | Malignant neoplasm: Liver cell carcinoma |
| Cancer | C913 | Prolymphocytic leukaemia |
| Cancer | C835 | Lymphoblastic (diffuse) lymphoma |
| Cancer | C432 | Malignant neoplasm: Malignant melanoma of ear and external auricular canal |
| Cancer | C399 | Malignant neoplasm: Ill-defined sites within the respiratory system |
| Cancer | C943 | Mast cell leukaemia |
| Cancer | C768 | Malignant neoplasm of other and ill-defined sites: Overlapping lesion of other and ill-defined sites |
| Cancer | C430 | Malignant neoplasm: Malignant melanoma of lip |
| Cancer | C410 | Malignant neoplasm: Bones of skull and face |
| Cancer | C759 | Malignant neoplasm: Endocrine gland, unspecified |
| Cancer | C493 | Malignant neoplasm: Connective and soft tissue of thorax |
| Cancer | C461 | Kaposi's sarcoma of soft tissue |
| Cancer | C841 | [NULL] |
| Cancer | C258 | Malignant neoplasm: Overlapping lesion of pancreas |
| Cancer | C920 | Acute myeloblastic leukaemia [AML] |
| Cancer | C009 | Malignant neoplasm: Lip, unspecified |
| Cancer | C23X | Malignant neoplasm of gallbladder |
| Cancer | C041 | Malignant neoplasm: Lateral floor of mouth |
| Cancer | C54 | Malignant neoplasm of corpus uteri |
| Cancer | C857 | Other specified types of non-Hodgkin's lymphoma |
| Cancer | C436 | Malignant neoplasm: Malignant melanoma of upper limb, including shoulder |
| Cancer | C960 | Letterer-Siwe disease |
| Cancer | C38 | Malignant neoplasm of heart, mediastinum and pleura |
| Cancer | C672 | Malignant neoplasm: Lateral wall of bladder |
| Cancer | C957 | Other leukaemia of unspecified cell type |
| Cancer | C450 | Mesothelioma of pleura |
| Cancer | C859 | Non-Hodgkin lymphoma, unspecified |
| Cancer | C451 | Mesothelioma of peritoneum |
| Cancer | C104 | Malignant neoplasm: Branchial cleft |
| Cancer | C452 | Mesothelioma of pericardium |
| Cancer | C435 | Malignant neoplasm: Malignant melanoma of trunk |
| Cancer | C889 | Malignant immunoproliferative disease, unspecified |
| Cancer | C240 | Malignant neoplasm: Extrahepatic bile duct |
| Cancer | C751 | Malignant neoplasm: Pituitary gland |
| Cancer | C188 | Malignant neoplasm: Overlapping lesion of colon |
| Cancer | C024 | Malignant neoplasm: Lingual tonsil |
| Cancer | C84 | Peripheral and cutaneous T-cell lymphomas |
| Cancer | C447 | Malignant neoplasm: Skin of lower limb, including hip |
| Cancer | C676 | Malignant neoplasm: Ureteric orifice |
| Cancer | C328 | Malignant neoplasm: Overlapping lesion of larynx |
| Cancer | C947 | Other specified leukaemias |
| Cancer | C186 | Malignant neoplasm: Descending colon |
| Cancer | C001 | Malignant neoplasm: External lower lip |
| Cancer | C002 | Malignant neoplasm: External lip, unspecified |
| Cancer | C944 | Acute panmyelosis |
| Cancer | C153 | Malignant neoplasm: Upper third of oesophagus |
| Cancer | C719 | Malignant neoplasm: Brain, unspecified |
| Cancer | C63 | Malignant neoplasm of other and unspecified male genital organs |
| Cancer | C951 | Chronic leukaemia of unspecified cell type |
| Cancer | C675 | Malignant neoplasm: Bladder neck |
| Cancer | C917 | Other lymphoid leukaemia |
| Cancer | C500 | Malignant neoplasm: Nipple and areola |
| Cancer | C760 | Malignant neoplasm of other and ill-defined sites: Head, face and neck |
| Cancer | C80 | Malignant neoplasm without specification of site |
| Cancer | C419 | Malignant neoplasm: Bone and articular cartilage, unspecified |
| Cancer | C541 | Malignant neoplasm: Endometrium |
| Cancer | C060 | Malignant neoplasm: Cheek mucosa |
| Cancer | C491 | Malignant neoplasm: Connective and soft tissue of upper limb, including shoulder |
| Cancer | C029 | Malignant neoplasm: Tongue, unspecified |
| Cancer | C400 | Malignant neoplasm: Scapula and long bones of upper limb |
| Cancer | C711 | Malignant neoplasm: Frontal lobe |
| Cancer | C813 | Lymphocyte depleted classical Hodgkin lymphoma |
| Cancer | C925 | Acute myelomonocytic leukaemia |
| Cancer | C496 | Malignant neoplasm: Connective and soft tissue of trunk, unspecified |
| Cancer | C44 | Other malignant neoplasms of skin |
| Cancer | C341 | Malignant neoplasm: Upper lobe, bronchus or lung |
| Cancer | C181 | Malignant neoplasm: Appendix |
| Cancer | C692 | Malignant neoplasm: Retina |
| Cancer | C110 | Malignant neoplasm: Superior wall of nasopharynx |
| Cancer | C494 | Malignant neoplasm: Connective and soft tissue of abdomen |
| Cancer | C572 | Malignant neoplasm: Round ligament |
| Cancer | C118 | Malignant neoplasm: Overlapping lesion of nasopharynx |
| Cancer | C398 | Malignant neoplasm: Overlapping lesion of respiratory and intrathoracic organs |
| Cancer | C33X | Malignant neoplasm of trachea |
| Cancer | C04 | Malignant neoplasm of floor of mouth |
| Cancer | C437 | Malignant neoplasm: Malignant melanoma of lower limb, including hip |
| Cancer | C03 | Malignant neoplasm of gum |
| Cancer | C880 | [NULL] |
| Cancer | C028 | Malignant neoplasm: Overlapping lesion of tongue |
| Cancer | C342 | Malignant neoplasm: Middle lobe, bronchus or lung |
| Cancer | C468 | Kaposi's sarcoma of multiple organs |
| Cancer | C883 | Immunoproliferative small intestinal disease |
| Cancer | C902 | Plasmacytoma, extramedullary |
| Cancer | C154 | Malignant neoplasm: Middle third of oesophagus |
| Cancer | C73X | Malignant neoplasm of thyroid gland |
| Cancer | C673 | Malignant neoplasm: Anterior wall of bladder |
| Cancer | C37X | Malignant neoplasm of thymus |
| Cancer | C119 | Malignant neoplasm: Nasopharynx, unspecified |
| Cancer | C165 | Malignant neoplasm: Lesser curvature of stomach, unspecified |
| Cancer | C571 | Malignant neoplasm: Broad ligament |
| Cancer | C003 | Malignant neoplasm: Upper lip, inner aspect |
| Cancer | C969 | Malignant neoplasm of lymphoid, haematopoietic and related tissue, unspecified |
| Cancer | C512 | Malignant neoplasm: Clitoris |
| Cancer | C69 | Malignant neoplasm of eye and adnexa |
| Cancer | C457 | Mesothelioma of other sites |
| Cancer | C53 | Malignant neoplasm of cervix uteri |
| Cancer | C184 | Malignant neoplasm: Transverse colon |
| Cancer | C222 | Malignant neoplasm: Hepatoblastoma |
| Cancer | C573 | Malignant neoplasm: Parametrium |
| Cancer | C409 | Malignant neoplasm: Bone and articular cartilage of limb, unspecified |
| Cancer | C069 | Malignant neoplasm: Mouth, unspecified |
| Cancer | C318 | Malignant neoplasm: Overlapping lesion of accessory sinuses |
| Cancer | C56X | Malignant neoplasm of ovary |
| Cancer | C108 | Malignant neoplasm: Overlapping lesion of oropharynx |
| Cancer | C674 | Malignant neoplasm: Posterior wall of bladder |
| Cancer | C754 | Malignant neoplasm: Carotid body |
| Cancer | C713 | Malignant neoplasm: Parietal lobe |
| Cancer | C55X | Malignant neoplasm of uterus, part unspecified |
| Cancer | C911 | Chronic lymphocytic leukaemia of B-cell type |
| Cancer | C269 | Malignant neoplasm: Ill-defined sites within the digestive system |
| Cancer | C138 | Malignant neoplasm: Overlapping lesion of hypopharynx |
| Cancer | C922 | Subacute myeloid leukaemia |
| Cancer | C340 | Malignant neoplasm: Main bronchus |
| Cancer | C000 | Malignant neoplasm: External upper lip |
| Cancer | C502 | Malignant neoplasm: Upper-inner quadrant of breast |
| Cancer | C348 | Malignant neoplasm: Overlapping lesion of bronchus and lung |
| Cancer | C061 | Malignant neoplasm: Vestibule of mouth |
| Cancer | C080 | Malignant neoplasm: Submandibular gland |
| Cancer | C349 | Malignant neoplasm: Bronchus or lung, unspecified |
| Cancer | C851 | B-cell lymphoma, unspecified |
| Cancer | C431 | Malignant neoplasm: Malignant melanoma of eyelid, including canthus |
| Cancer | C549 | Malignant neoplasm: Corpus uteri, unspecified |
| Cancer | C689 | Malignant neoplasm: Urinary organ, unspecified |
| Cancer | C538 | Malignant neoplasm: Overlapping lesion of cervix uteri |
| Cancer | C680 | Malignant neoplasm: Urethra |
| Cancer | C519 | Malignant neoplasm: Vulva, unspecified |
| Cancer | C301 | Malignant neoplasm: Middle ear |
| Cancer | C390 | Malignant neoplasm: Upper respiratory tract, part unspecified |
| Cancer | C021 | Malignant neoplasm: Border of tongue |
| Cancer | C112 | Malignant neoplasm: Lateral wall of nasopharynx |
| Cancer | C166 | Malignant neoplasm: Greater curvature of stomach, unspecified |
| Cancer | C812 | Mixed cellularity (classical) Hodgkin lymphoma |
| Cancer | C01X | Malignant neoplasm of base of tongue |
| Cancer | C83 | Diffuse non-Hodgkin's lymphoma |
| Cancer | C488 | Malignant neoplasm: Overlapping lesion of retroperitoneum and peritoneum |
| Cancer | C66X | Malignant neoplasm of ureter |
| Cancer | C11 | Malignant neoplasm of nasopharynx |
| Cancer | C833 | Diffuse large B-cell lymphoma |
| Cancer | C481 | Malignant neoplasm: Specified parts of peritoneum |
| Cancer | C927 | Other myeloid leukaemia |
| Cancer | C940 | Acute erythraemia and erythroleukaemia |
| Cancer | C40 | Malignant neoplasm of bone and articular cartilage of limbs |
| Cancer | C577 | Malignant neoplasm: Other specified female genital organs |
| Cancer | C839 | Non-follicular (diffuse) lymphoma, unspecified |
| Cancer | C081 | Malignant neoplasm: Sublingual gland |
| Cancer | C17 | Malignant neoplasm of small intestine |
| Cancer | C26 | Malignant neoplasm of other and ill-defined digestive organs |
| Cancer | C030 | Malignant neoplasm: Upper gum |
| Cancer | C62 | Malignant neoplasm of testis |
| Cancer | C438 | Malignant neoplasm: Overlapping malignant melanoma of skin |
| Cancer | C820 | Follicular lymphoma grade I |
| Cancer | C837 | Burkitt's tumour |
| Cancer | C83 | Non-follicular lymphoma |
| Cancer | C910 | Acute lymphoblastic leukaemia |
| Cancer | C829 | Follicular non-Hodgkin's lymphoma, unspecified |
| Cancer | C159 | Malignant neoplasm: Oesophagus, unspecified |
| Cancer | C16 | Malignant neoplasm of stomach |
| Cancer | C300 | Malignant neoplasm: Nasal cavity |
| Cancer | C924 | Acute promyelocytic leukaemia [PML] |
| Cancer | C695 | Malignant neoplasm: Lacrimal gland and duct |
| Cancer | C179 | Malignant neoplasm: Small intestine, unspecified |
| Cancer | C962 | Malignant mast cell tumour |
| Cancer | C408 | Malignant neoplasm: Overlapping lesion of bone and articular cartilage of limbs |
| Cancer | C698 | Malignant neoplasm: Overlapping lesion of eye and adnexa |
| Cancer | C921 | Chronic myeloid leukaemia |
| Cancer | C750 | Malignant neoplasm: Parathyroid gland |
| Cancer | C08 | Malignant neoplasm of other and unspecified major salivary glands |
| Cancer | C950 | Acute leukaemia of unspecified cell type |
| Cancer | C139 | Malignant neoplasm: Hypopharynx, unspecified |
| Cancer | C13 | Malignant neoplasm of hypopharynx |
| Cancer | C940 | Acute erythroid leukaemia |
| Cancer | C678 | Malignant neoplasm: Overlapping lesion of bladder |
| Cancer | C900 | Multiple myeloma |
| Cancer | C142 | Malignant neoplasm: Waldeyer ring |
| Cancer | C040 | Malignant neoplasm: Anterior floor of mouth |
| Cancer | C540 | Malignant neoplasm: Isthmus uteri |
| Cancer | C10 | Malignant neoplasm of oropharynx |
| Cancer | C701 | Malignant neoplasm: Spinal meninges |
| Cancer | C168 | Malignant neoplasm: Overlapping lesion of stomach |
| Cancer | C931 | Chronic monocytic leukaemia |
| Cancer | C320 | Malignant neoplasm: Glottis |
| Cancer | C52X | Malignant neoplasm of vagina |
| Cancer | C210 | Malignant neoplasm: Anus, unspecified |
| Cancer | C82 | Follicular lymphoma |
| Cancer | C161 | Malignant neoplasm: Fundus of stomach |
| Cancer | C310 | Malignant neoplasm: Maxillary sinus |
| Cancer | C710 | Malignant neoplasm: Cerebrum, except lobes and ventricles |
| Cancer | C915 | Adult T-cell leukaemia |
| Cancer | C959 | Leukaemia, unspecified |
| Cancer | C241 | Malignant neoplasm: Ampulla of Vater |
| Cancer | C76 | Malignant neoplasm of other and ill-defined sites |
| Cancer | C461 | Kaposi sarcoma of soft tissue |
| Cancer | C819 | Hodgkin lymphoma, unspecified |
| Cancer | C006 | Malignant neoplasm: Commissure of lip |
| Cancer | C460 | Kaposi sarcoma of skin |
| Cancer | C887 | Other malignant immunoproliferative diseases |
| Cancer | C472 | Malignant neoplasm: Peripheral nerves of lower limb, including hip |
| Cancer | C140 | Malignant neoplasm: Pharynx, unspecified |
| Cancer | C07X | Malignant neoplasm of parotid gland |
| Cancer | C111 | Malignant neoplasm: Posterior wall of nasopharynx |
| Cancer | C30 | Malignant neoplasm of nasal cavity and middle ear |
| Cancer | C819 | Hodgkin's disease: Hodgkin's disease, unspecified |
| Cancer | C00 | Malignant neoplasm of lip |
| Cancer | C475 | Malignant neoplasm: Peripheral nerves of pelvis |
| Cancer | C50 | Malignant neoplasm of breast |
| Cancer | C21 | Malignant neoplasm of anus and anal canal |
| Cancer | C753 | Malignant neoplasm: Pineal gland |
| Cancer | C915 | Adult T-cell lymphoma/leukaemia [HTLV-1-associated] |
| Cancer | C840 | Mycosis fungoides |
| Cancer | C831 | Mantle cell lymphoma |
| Cancer | C439 | Malignant neoplasm: Malignant melanoma of skin, unspecified |
| Cancer | C004 | Malignant neoplasm: Lower lip, inner aspect |
| Cancer | C511 | Malignant neoplasm: Labium minus |
| Cancer | C831 | Non-Hodgkin's lymphoma: Small cleaved cell (diffuse) |
| Cancer | C930 | Acute monocytic leukaemia |
| Cancer | C752 | Malignant neoplasm: Craniopharyngeal duct |
| Cancer | C820 | Non-Hodgkin's lymphoma: Small cleaved cell, follicular |
| Cancer | C857 | Other specified types of non-Hodgkin lymphoma |
| Cancer | C212 | Malignant neoplasm: Cloacogenic zone |
| Cancer | C250 | Malignant neoplasm: Head of pancreas |
| Cancer | C221 | Malignant neoplasm: Intrahepatic bile duct carcinoma |
| Cancer | C75 | Malignant neoplasm of other endocrine glands and related structures |
| Cancer | C051 | Malignant neoplasm: Soft palate |
| Cancer | C170 | Malignant neoplasm: Duodenum |
| Cancer | C817 | Other (classical) Hodgkin lymphoma |
| Cancer | C098 | Malignant neoplasm: Overlapping lesion of tonsil |
| Cancer | C709 | Malignant neoplasm: Meninges, unspecified |
| Cancer | C859 | Non-Hodgkin's lymphoma, unspecified type |
| Cancer | C960 | Multifocal and multisystemic (disseminated) Langerhans-cell histiocytosis [Letterer-Siwe disease] |
| Cancer | C434 | Malignant neoplasm: Malignant melanoma of scalp and neck |
| Cancer | C433 | Malignant neoplasm: Malignant melanoma of other and unspecified parts of face |
| Cancer | C833 | Non-Hodgkin's lymphoma: Large cell (diffuse) |
| Cancer | C539 | Malignant neoplasm: Cervix uteri, unspecified |
| Cancer | C51 | Malignant neoplasm of vulva |
| Cancer | C411 | Malignant neoplasm: Mandible |
| Cancer | C761 | Malignant neoplasm of other and ill-defined sites: Thorax |
| Cancer | C469 | Kaposi's sarcoma, unspecified |
| Cancer | C93 | Monocytic leukaemia |
| Cancer | C113 | Malignant neoplasm: Anterior wall of nasopharynx |
| Cancer | C579 | Malignant neoplasm: Female genital organ, unspecified |
| Cancer | C005 | Malignant neoplasm: Lip, unspecified, inner aspect |
| Cancer | C821 | Non-Hodgkin's lymphoma: Mixed small cleaved and large cell, follicular |
| Cancer | C942 | Acute megakaryoblastic leukaemia |
| Cancer | C57 | Malignant neoplasm of other and unspecified female genital organs |
| Cancer | C65X | Malignant neoplasm of renal pelvis |
| Cancer | C322 | Malignant neoplasm: Subglottis |
| Cancer | C34 | Malignant neoplasm of bronchus and lung |
| Cancer | C696 | Malignant neoplasm: Orbit |
| Cancer | C151 | Malignant neoplasm: Thoracic part of oesophagus |
| Cancer | C479 | Malignant neoplasm: Peripheral nerves and autonomic nervous system, unspecified |
| Cancer | C474 | Malignant neoplasm: Peripheral nerves of abdomen |
| Cancer | C45 | Mesothelioma |
| Cancer | C621 | Malignant neoplasm: Descended testis |
| Cancer | C681 | Malignant neoplasm: Paraurethral gland |
| Cancer | C531 | Malignant neoplasm: Exocervix |
| Cancer | C671 | Malignant neoplasm: Dome of bladder |
| Cancer | C835 | Non-Hodgkin's lymphoma: Lymphoblastic (diffuse) |
| Cancer | C499 | Malignant neoplasm: Connective and soft tissue, unspecified |
| Cancer | C764 | Malignant neoplasm of other and ill-defined sites: Upper limb |
| Cancer | C728 | Malignant neoplasm: Overlapping lesion of brain and other parts of central nervous system |
| Cancer | C058 | Malignant neoplasm: Overlapping lesion of palate |
| Cancer | C631 | Malignant neoplasm: Spermatic cord |
| Cancer | C183 | Malignant neoplasm: Hepatic flexure |
| Cancer | C155 | Malignant neoplasm: Lower third of oesophagus |
| Cancer | C578 | Malignant neoplasm: Overlapping lesion of female genital organs |
| Cancer | C839 | Diffuse non-Hodgkin's lymphoma, unspecified |
| Cancer | C931 | Chronic myelomonocytic leukaemia |
| Cancer | C402 | Malignant neoplasm: Long bones of lower limb |
| Cancer | C811 | Hodgkin's disease: Nodular sclerosis |
| Cancer | C812 | Hodgkin's disease: Mixed cellularity |
| Cancer | C755 | Malignant neoplasm: Aortic body and other paraganglia |
| Cancer | C172 | Malignant neoplasm: Ileum |
| Cancer | C677 | Malignant neoplasm: Urachus |
| Cancer | C813 | Lymphocyte depleted (classical) Hodgkin lymphoma |
| Cancer | C85 | Other and unspecified types of non-Hodgkin lymphoma |
| Cancer | C688 | Malignant neoplasm: Overlapping lesion of urinary organs |
| Cancer | C20X | Malignant neoplasm of rectum |
| Cancer | C490 | Malignant neoplasm: Connective and soft tissue of head, face and neck |
| Cancer | C82 | Follicular [nodular] non-Hodgkin's lymphoma |
| Cancer | C543 | Malignant neoplasm: Fundus uteri |
| Cancer | C319 | Malignant neoplasm: Accessory sinus, unspecified |
| Cancer | C817 | Other classical Hodgkin lymphoma |
| Cancer | C96 | Other and unspecified malignant neoplasms of lymphoid, haematopoietic and related tissue |
| Cancer | C445 | Malignant neoplasm: Skin of trunk |
| Cancer | C444 | Malignant neoplasm: Skin of scalp and neck |
| Cancer | C260 | Malignant neoplasm: Intestinal tract, part unspecified |
| Cancer | C60 | Malignant neoplasm of penis |
| Cancer | C448 | Malignant neoplasm: Overlapping lesion of skin |
| Cancer | C12X | Malignant neoplasm of piriform sinus |
| Cancer | C164 | Malignant neoplasm: Pylorus |
| Cancer | C722 | Malignant neoplasm: Olfactory nerve |
| Cancer | C911 | Chronic lymphocytic leukaemia |
| Cancer | C446 | Malignant neoplasm: Skin of upper limb, including shoulder |
| Cancer | C608 | Malignant neoplasm: Overlapping lesion of penis |
| Cancer | C381 | Malignant neoplasm: Anterior mediastinum |
| Cancer | C023 | Malignant neoplasm: Anterior two-thirds of tongue, part unspecified |
| Cancer | C700 | Malignant neoplasm: Cerebral meninges |
| Cancer | C691 | Malignant neoplasm: Cornea |
| Cancer | C163 | Malignant neoplasm: Pyloric antrum |
| Cancer | C498 | Malignant neoplasm: Overlapping lesion of connective and soft tissue |
| Cancer | C602 | Malignant neoplasm: Body of penis |
| Cancer | C690 | Malignant neoplasm: Conjunctiva |
| Cancer | C09 | Malignant neoplasm of tonsil |
| Cancer | C639 | Malignant neoplasm: Male genital organ, unspecified |
| Cancer | C261 | Malignant neoplasm: Spleen |
| Cancer | C720 | Malignant neoplasm: Spinal cord |
| Cancer | C944 | Acute panmyelosis with myelofibrosis |
| Cancer | C714 | Malignant neoplasm: Occipital lobe |
| Cancer | C443 | Malignant neoplasm: Skin of other and unspecified parts of face |
| Cancer | C211 | Malignant neoplasm: Anal canal |
| Cancer | C091 | Malignant neoplasm: Tonsillar pillar (anterior)(posterior) |
| Cancer | C505 | Malignant neoplasm: Lower-outer quadrant of breast |
| Cancer | C478 | Malignant neoplasm: Overlapping lesion of peripheral nerves and autonomic nervous system |
| Cancer | C048 | Malignant neoplasm: Overlapping lesion of floor of mouth |
| Cancer | C508 | Malignant neoplasm: Overlapping lesion of breast |
| Cancer | C510 | Malignant neoplasm: Labium majus |
| Cancer | C268 | Malignant neoplasm: Overlapping lesion of digestive system |
| Cancer | C844 | Peripheral T-cell lymphoma, not elsewhere classified |
| Cancer | C24 | Malignant neoplasm of other and unspecified parts of biliary tract |
| Cancer | C463 | Kaposi sarcoma of lymph nodes |
| Cancer | C929 | Myeloid leukaemia, unspecified |
| Cancer | C152 | Malignant neoplasm: Abdominal part of oesophagus |
| Cancer | C830 | Non-Hodgkin's lymphoma: Small cell (diffuse) |
| Cancer | C254 | Malignant neoplasm: Endocrine pancreas |
| Cancer | C810 | Hodgkin's disease: Lymphocytic predominance |
| Cancer | C476 | Malignant neoplasm: Peripheral nerves of trunk, unspecified |
| Cancer | C473 | Malignant neoplasm: Peripheral nerves of thorax |
| Cancer | C14 | Malignant neoplasm of other and ill-defined sites in the lip, oral cavity and pharynx |
| Cancer | C130 | Malignant neoplasm: Postcricoid region |
| Cancer | C32 | Malignant neoplasm of larynx |
| Cancer | C882 | Other heavy chain disease |
| Cancer | C039 | Malignant neoplasm: Gum, unspecified |
| Cancer | C495 | Malignant neoplasm: Connective and soft tissue of pelvis |
| Cancer | C518 | Malignant neoplasm: Overlapping lesion of vulva |
| Cancer | C227 | Malignant neoplasm: Other specified carcinomas of liver |
| Cancer | C480 | Malignant neoplasm: Retroperitoneum |
| Cancer | C741 | Malignant neoplasm: Medulla of adrenal gland |
| Cancer | C22 | Malignant neoplasm of liver and intrahepatic bile ducts |
| Cancer | C19X | Malignant neoplasm of rectosigmoid junction |
| Cancer | C440 | Malignant neoplasm: Skin of lip |
| Cancer | C059 | Malignant neoplasm: Palate, unspecified |
| Cancer | C72 | Malignant neoplasm of spinal cord, cranial nerves and other parts of central nervous system |
| Cancer | C923 | Myeloid sarcoma |
| Cancer | C811 | Nodular sclerosis (classical) Hodgkin lymphoma |
| Cancer | C810 | Nodular lymphocyte predominant Hodgkin lymphoma |
| Cancer | C185 | Malignant neoplasm: Splenic flexure |
| Cancer | C18 | Malignant neoplasm of colon |
| Cancer | C259 | Malignant neoplasm: Pancreas, unspecified |
| Cancer | C463 | Kaposi's sarcoma of lymph nodes |
| Cancer | C412 | Malignant neoplasm: Vertebral column |
| Cancer | C224 | Malignant neoplasm: Other sarcomas of liver |
| Cancer | C343 | Malignant neoplasm: Lower lobe, bronchus or lung |
| Cancer | C49 | Malignant neoplasm of other connective and soft tissue |
| Cancer | C022 | Malignant neoplasm: Ventral surface of tongue |
| Cancer | C257 | Malignant neoplasm: Other parts of pancreas |
| Cancer | C469 | Kaposi sarcoma, unspecified |
| Cancer | C252 | Malignant neoplasm: Tail of pancreas |
| Cancer | C503 | Malignant neoplasm: Lower-inner quadrant of breast |
| Cancer | C008 | Malignant neoplasm: Overlapping lesion of lip |
| Cancer | C821 | Follicular lymphoma grade II |
| Cancer | C725 | Malignant neoplasm: Other and unspecified cranial nerves |
| Cancer | C712 | Malignant neoplasm: Temporal lobe |
| Cancer | C229 | Malignant neoplasm: Liver, unspecified |
| Cancer | C902 | Extramedullary plasmacytoma |
| Cancer | C638 | Malignant neoplasm: Overlapping lesion of male genital organs |
| Cancer | C460 | Kaposi's sarcoma of skin |
| Cancer | C189 | Malignant neoplasm: Colon, unspecified |
| Cancer | C471 | Malignant neoplasm: Peripheral nerves of upper limb, including shoulder |
| Cancer | C384 | Malignant neoplasm: Pleura |
| Cancer | C158 | Malignant neoplasm: Overlapping lesion of oesophagus |
| Cancer | C844 | Peripheral T-cell lymphoma |
| Cancer | C901 | Plasma cell leukaemia |
| Cancer | C442 | Malignant neoplasm: Skin of ear and external auricular canal |
| Cancer | C630 | Malignant neoplasm: Epididymis |
| Cancer | C162 | Malignant neoplasm: Body of stomach |
| Cancer | C223 | Malignant neoplasm: Angiosarcoma of liver |
| Cancer | C670 | Malignant neoplasm: Trigone of bladder |
| Cancer | C74 | Malignant neoplasm of adrenal gland |
| Cancer | C441 | Malignant neoplasm: Skin of eyelid, including canthus |
| Cancer | C449 | Malignant neoplasm: Malignant neoplasm of skin, unspecified |
| Cancer | C182 | Malignant neoplasm: Ascending colon |
| Cancer | C253 | Malignant neoplasm: Pancreatic duct |
| Cancer | C765 | Malignant neoplasm of other and ill-defined sites: Lower limb |
| Cancer | C92 | Myeloid leukaemia |
| Cancer | C767 | Malignant neoplasm of other and ill-defined sites: Other ill-defined sites |
| Cancer | C403 | Malignant neoplasm: Short bones of lower limb |
| Cancer | C413 | Malignant neoplasm: Ribs, sternum and clavicle |
| Cancer | C131 | Malignant neoplasm: Aryepiglottic fold, hypopharyngeal aspect |
| Cancer | C132 | Malignant neoplasm: Posterior wall of hypopharynx |
| Cancer | C85 | Other and unspecified types of non-Hodgkin's lymphoma |
| Cancer | C323 | Malignant neoplasm: Laryngeal cartilage |
| Cancer | C46 | Kaposi's sarcoma |
| Cancer | C100 | Malignant neoplasm: Vallecula |
| Cancer | C629 | Malignant neoplasm: Testis, unspecified |
| Cancer | C762 | Malignant neoplasm of other and ill-defined sites: Abdomen |
| Cancer | C46 | Kaposi sarcoma |
| Cancer | C088 | Malignant neoplasm: Overlapping lesion of major salivary glands |
| Cancer | C699 | Malignant neoplasm: Eye, unspecified |
| Cancer | C70 | Malignant neoplasm of meninges |
| Cancer | C148 | Malignant neoplasm: Overlapping lesion of lip, oral cavity and pharynx |
| Cancer | C47 | Malignant neoplasm of peripheral nerves and autonomic nervous system |
| Cancer | C919 | Lymphoid leukaemia, unspecified |
| Cancer | C609 | Malignant neoplasm: Penis, unspecified |
| Cancer | C39 | Malignant neoplasm of other and ill-defined sites in the respiratory system and intrathoracic organs |
| Cancer | C173 | Malignant neoplasm: Meckel diverticulum |
| Cancer | C723 | Malignant neoplasm: Optic nerve |
| Cancer | C967 | Other specified malignant neoplasms of lymphoid, haematopoietic and related tissue |
| Cancer | C740 | Malignant neoplasm: Cortex of adrenal gland |
| Cancer | C506 | Malignant neoplasm: Axillary tail of breast |
| Cancer | C312 | Malignant neoplasm: Frontal sinus |
| Cancer | C827 | Other types of follicular non-Hodgkin's lymphoma |
| Cancer | C382 | Malignant neoplasm: Posterior mediastinum |
| Cancer | C109 | Malignant neoplasm: Oropharynx, unspecified |
| Cancer | C248 | Malignant neoplasm: Overlapping lesion of biliary tract |
| Cancer | C160 | Malignant neoplasm: Cardia |
| Cancer | C249 | Malignant neoplasm: Biliary tract, unspecified |
| Cancer | C637 | Malignant neoplasm: Other specified male genital organs |
| Cancer | C492 | Malignant neoplasm: Connective and soft tissue of lower limb, including hip |
| Cancer | C601 | Malignant neoplasm: Glans penis |
| Cancer | C882 | Gamma heavy chain disease |
| Cancer | C632 | Malignant neoplasm: Scrotum |
| Cancer | C02 | Malignant neoplasm of other and unspecified parts of tongue |
| Cancer | C922 | Atypical chronic myeloid leukaemia, BCR/ABL-negative |
| Cancer | C504 | Malignant neoplasm: Upper-outer quadrant of breast |
| Cancer | C25 | Malignant neoplasm of pancreas |
| Cancer | C401 | Malignant neoplasm: Short bones of upper limb |
| Cancer | C64X | Malignant neoplasm of kidney, except renal pelvis |
| Cancer | C913 | Prolymphocytic leukaemia of B-cell type |
| Cancer | C482 | Malignant neoplasm: Peritoneum, unspecified |
| Cancer | C717 | Malignant neoplasm: Brain stem |
| Cancer | C620 | Malignant neoplasm: Undescended testis |
| Cancer | C822 | Non-Hodgkin's lymphoma: Large cell, follicular |
| Cancer | C715 | Malignant neoplasm: Cerebral ventricle |
| Cancer | C758 | Malignant neoplasm: Pluriglandular involvement, unspecified |
| Cancer | C099 | Malignant neoplasm: Tonsil, unspecified |
| Cancer | C380 | Malignant neoplasm: Heart |
| Cancer | C467 | Kaposi's sarcoma of other sites |
| Cancer | C462 | Kaposi's sarcoma of palate |
| Cancer | C841 | [NULL] |
| Cancer | C031 | Malignant neoplasm: Lower gum |
| Cancer | C817 | Hodgkin's disease: Other Hodgkin's disease |
| Cancer | C090 | Malignant neoplasm: Tonsillar fossa |
| Cancer | C570 | Malignant neoplasm: Fallopian tube |
| Cancer | C311 | Malignant neoplasm: Ethmoidal sinus |
| Cancer | C41 | Malignant neoplasm of bone and articular cartilage of other and unspecified sites |
| Cancer | C58X | Malignant neoplasm of placenta |
| Cancer | C020 | Malignant neoplasm: Dorsal surface of tongue |
| Cancer | C729 | Malignant neoplasm: Central nervous system, unspecified |
| Cancer | C329 | Malignant neoplasm: Larynx, unspecified |
| Diabetes | E113 | Non-insulin-dependent diabetes mellitus |
| Diabetes | E114 | Non-insulin-dependent diabetes mellitus |
| Diabetes | E110 | Type 2 diabetes mellitus |
| Diabetes | E137 | Other specified diabetes mellitus |
| Diabetes | E146 | Unspecified diabetes mellitus |
| Diabetes | E136 | Other specified diabetes mellitus |
| Diabetes | E112 | Type 2 diabetes mellitus |
| Diabetes | E111 | Type 2 diabetes mellitus |
| Diabetes | E11 | Non-insulin-dependent diabetes mellitus |
| Diabetes | E112 | Non-insulin-dependent diabetes mellitus |
| Diabetes | E144 | Unspecified diabetes mellitus |
| Diabetes | E118 | Type 2 diabetes mellitus |
| Diabetes | E125 | Malnutrition-related diabetes mellitus |
| Diabetes | E121 | Malnutrition-related diabetes mellitus |
| Diabetes | E138 | Other specified diabetes mellitus |
| Diabetes | E135 | Other specified diabetes mellitus |
| Diabetes | E117 | Type 2 diabetes mellitus |
| Diabetes | E145 | Unspecified diabetes mellitus |
| Diabetes | E120 | Malnutrition-related diabetes mellitus |
| Diabetes | E147 | Unspecified diabetes mellitus |
| Diabetes | E13 | Other specified diabetes mellitus |
| Diabetes | E131 | Other specified diabetes mellitus |
| Diabetes | E140 | Unspecified diabetes mellitus |
| Diabetes | E143 | Unspecified diabetes mellitus |
| Diabetes | E111 | Non-insulin-dependent diabetes mellitus |
| Diabetes | E130 | Other specified diabetes mellitus |
| Diabetes | E122 | Malnutrition-related diabetes mellitus |
| Diabetes | E149 | Unspecified diabetes mellitus |
| Diabetes | E110 | Non-insulin-dependent diabetes mellitus |
| Diabetes | E117 | Non-insulin-dependent diabetes mellitus |
| Diabetes | E128 | Malnutrition-related diabetes mellitus |
| Diabetes | E127 | Malnutrition-related diabetes mellitus |
| Diabetes | E114 | Type 2 diabetes mellitus |
| Diabetes | E129 | Malnutrition-related diabetes mellitus |
| Diabetes | E115 | Non-insulin-dependent diabetes mellitus |
| Diabetes | E139 | Other specified diabetes mellitus |
| Diabetes | E148 | Unspecified diabetes mellitus |
| Diabetes | E123 | Malnutrition-related diabetes mellitus |
| Diabetes | E126 | Malnutrition-related diabetes mellitus |
| Diabetes | E119 | Non-insulin-dependent diabetes mellitus |
| Diabetes | E116 | Non-insulin-dependent diabetes mellitus |
| Diabetes | E133 | Other specified diabetes mellitus |
| Diabetes | E142 | Unspecified diabetes mellitus |
| Diabetes | E12 | Malnutrition-related diabetes mellitus |
| Diabetes | E113 | Type 2 diabetes mellitus |
| Diabetes | E134 | Other specified diabetes mellitus |
| Diabetes | E116 | Type 2 diabetes mellitus |
| Diabetes | E118 | Non-insulin-dependent diabetes mellitus |
| Diabetes | E11 | Type 2 diabetes mellitus |
| Diabetes | E124 | Malnutrition-related diabetes mellitus |
| Diabetes | E119 | Type 2 diabetes mellitus |
| Diabetes | E141 | Unspecified diabetes mellitus |
| Diabetes | E132 | Other specified diabetes mellitus |
| Diabetes | E14 | Unspecified diabetes mellitus |
| Diabetes | E115 | Type 2 diabetes mellitus |
| Haemorrhagic stroke | I692 | Sequelae of other nontraumatic intracranial haemorrhage |
| Haemorrhagic stroke | I618 | Other intracerebral haemorrhage |
| Haemorrhagic stroke | I610 | Intracerebral haemorrhage in hemisphere, subcortical |
| Haemorrhagic stroke | I613 | Intracerebral haemorrhage in brain stem |
| Haemorrhagic stroke | I619 | Intracerebral haemorrhage, unspecified |
| Haemorrhagic stroke | I616 | Intracerebral haemorrhage, multiple localized |
| Haemorrhagic stroke | I615 | Intracerebral haemorrhage, intraventricular |
| Haemorrhagic stroke | I614 | Intracerebral haemorrhage in cerebellum |
| Haemorrhagic stroke | I691 | Sequelae of intracerebral haemorrhage |
| Haemorrhagic stroke | I612 | Intracerebral haemorrhage in hemisphere, unspecified |
| Haemorrhagic stroke | I629 | Intracranial haemorrhage (nontraumatic), unspecified |
| Haemorrhagic stroke | I611 | Intracerebral haemorrhage in hemisphere, cortical |
| Haemorrhagic stroke | I61 | Intracerebral haemorrhage |
| Heart failure | I509 | Heart failure, unspecified |
| Heart failure | I501 | Left ventricular failure |
| Heart failure | I500 | Congestive heart failure |
| Heart failure | I50 | Heart failure |
| Ischaemic heart disease | I211 | Acute transmural myocardial infarction of inferior wall |
| Ischaemic heart disease | I213 | Acute transmural myocardial infarction of unspecified site |
| Ischaemic heart disease | I258 | Other forms of chronic ischaemic heart disease |
| Ischaemic heart disease | I209 | Angina pectoris, unspecified |
| Ischaemic heart disease | I21 | Acute myocardial infarction |
| Ischaemic heart disease | I208 | Other forms of angina pectoris |
| Ischaemic heart disease | I210 | Acute transmural myocardial infarction of anterior wall |
| Ischaemic heart disease | I221 | Subsequent myocardial infarction of inferior wall |
| Ischaemic heart disease | I214 | Acute subendocardial myocardial infarction |
| Ischaemic heart disease | I251 | Atherosclerotic heart disease |
| Ischaemic heart disease | I212 | Acute transmural myocardial infarction of other sites |
| Ischaemic heart disease | I228 | Subsequent myocardial infarction of other sites |
| Ischaemic heart disease | I219 | Acute myocardial infarction, unspecified |
| Ischaemic heart disease | I220 | Subsequent myocardial infarction of anterior wall |
| Ischaemic heart disease | I22 | Subsequent myocardial infarction |
| Ischaemic heart disease | I201 | Angina pectoris with documented spasm |
| Ischaemic heart disease | I229 | Subsequent myocardial infarction of unspecified site |
| Ischaemic heart disease | I240 | Coronary thrombosis not resulting in myocardial infarction |
| Ischaemic heart disease | I250 | Atherosclerotic cardiovascular disease, so described |
| Ischaemic heart disease | I248 | Other forms of acute ischaemic heart disease |
| Ischaemic stroke | I661 | Occlusion and stenosis of anterior cerebral artery |
| Ischaemic stroke | I635 | Cerebral infarction due to unspecified occlusion or stenosis of cerebral arteries |
| Ischaemic stroke | I660 | Occlusion and stenosis of middle cerebral artery |
| Ischaemic stroke | I650 | Occlusion and stenosis of vertebral artery |
| Ischaemic stroke | I633 | Cerebral infarction due to thrombosis of cerebral arteries |
| Ischaemic stroke | I663 | Occlusion and stenosis of cerebellar arteries |
| Ischaemic stroke | I651 | Occlusion and stenosis of basilar artery |
| Ischaemic stroke | I631 | Cerebral infarction due to embolism of precerebral arteries |
| Ischaemic stroke | I652 | Occlusion and stenosis of carotid artery |
| Ischaemic stroke | I639 | Cerebral infarction, unspecified |
| Ischaemic stroke | G458 | Other transient cerebral ischaemic attacks and related syndromes |
| Ischaemic stroke | I632 | Cerebral infarction due to unspecified occlusion or stenosis of precerebral arteries |
| Ischaemic stroke | I664 | Occlusion and stenosis of multiple and bilateral cerebral arteries |
| Ischaemic stroke | I634 | Cerebral infarction due to embolism of cerebral arteries |
| Ischaemic stroke | I659 | Occlusion and stenosis of unspecified precerebral artery |
| Ischaemic stroke | I653 | Occlusion and stenosis of multiple and bilateral precerebral arteries |
| Ischaemic stroke | I638 | Other cerebral infarction |
| Ischaemic stroke | I630 | Cerebral infarction due to thrombosis of precerebral arteries |
| Ischaemic stroke | I636 | Cerebral infarction due to cerebral venous thrombosis, nonpyogenic |
| Ischaemic stroke | I662 | Occlusion and stenosis of posterior cerebral artery |
| Ischaemic stroke | I658 | Occlusion and stenosis of other precerebral artery |
| Ischaemic stroke | G459 | Transient cerebral ischaemic attack, unspecified |
| Ischaemic stroke | I63 | Cerebral infarction |
| Ischaemic stroke | I669 | Occlusion and stenosis of unspecified cerebral artery |
| Ischaemic stroke | I668 | Occlusion and stenosis of other cerebral artery |
| Liver disease | K744 | Secondary biliary cirrhosis |
| Liver disease | R162 | Hepatomegaly with splenomegaly, not elsewhere classified |
| Liver disease | R161 | Splenomegaly, not elsewhere classified |
| Liver disease | K715 | Toxic liver disease with chronic active hepatitis |
| Liver disease | K760 | Fatty (change of) liver, not elsewhere classified |
| Liver disease | R160 | Hepatomegaly, not elsewhere classified |
| Liver disease | K766 | Portal hypertension |
| Liver disease | R17X | Unspecified jaundice |
| Liver disease | K764 | Peliosis hepatis |
| Liver disease | K769 | Liver disease, unspecified |
| Liver disease | K770 | Liver disorders in infectious and parasitic diseases classified elsewhere |
| Liver disease | K762 | Central haemorrhagic necrosis of liver |
| Liver disease | K702 | Alcoholic fibrosis and sclerosis of liver |
| Liver disease | K703 | Alcoholic cirrhosis of liver |
| Liver disease | K701 | Alcoholic hepatitis |
| Liver disease | K751 | Phlebitis of portal vein |
| Liver disease | K765 | Hepatic veno-occlusive disease |
| Liver disease | K730 | Chronic persistent hepatitis, not elsewhere classified |
| Liver disease | K718 | Toxic liver disease with other disorders of liver |
| Liver disease | K759 | Inflammatory liver disease, unspecified |
| Liver disease | K739 | Chronic hepatitis, unspecified |
| Liver disease | K72 | Hepatic failure, not elsewhere classified |
| Liver disease | K758 | Other specified inflammatory liver diseases |
| Liver disease | K738 | Other chronic hepatitis, not elsewhere classified |
| Liver disease | K743 | Primary biliary cirrhosis |
| Liver disease | K70 | Alcoholic liver disease |
| Liver disease | K763 | Infarction of liver |
| Liver disease | K711 | Toxic liver disease with hepatic necrosis |
| Liver disease | K767 | Hepatorenal syndrome |
| Liver disease | K741 | Hepatic sclerosis |
| Liver disease | K761 | Chronic passive congestion of liver |
| Liver disease | R16 | Hepatomegaly and splenomegaly, not elsewhere classified |
| Liver disease | K768 | Other specified diseases of liver |
| Liver disease | K729 | Hepatic failure, unspecified |
| Liver disease | K717 | Toxic liver disease with fibrosis and cirrhosis of liver |
| Liver disease | K720 | Acute and subacute hepatic failure |
| Liver disease | K75 | Other inflammatory liver diseases |
| Liver disease | K709 | Alcoholic liver disease, unspecified |
| Liver disease | K746 | Other and unspecified cirrhosis of liver |
| Liver disease | K704 | Alcoholic hepatic failure |
| Liver disease | K778 | Liver disorders in other diseases classified elsewhere |
| Liver disease | K752 | Nonspecific reactive hepatitis |
| Liver disease | K714 | Toxic liver disease with chronic lobular hepatitis |
| Liver disease | K71 | Toxic liver disease |
| Liver disease | K716 | Toxic liver disease with hepatitis, not elsewhere classified |
| Liver disease | K745 | Biliary cirrhosis, unspecified |
| Liver disease | K731 | Chronic lobular hepatitis, not elsewhere classified |
| Liver disease | K76 | Other diseases of liver |
| Liver disease | K750 | Abscess of liver |
| Liver disease | K742 | Hepatic fibrosis with hepatic sclerosis |
| Liver disease | K700 | Alcoholic fatty liver |
| Liver disease | K753 | Granulomatous hepatitis, not elsewhere classified |
| Liver disease | K732 | Chronic active hepatitis, not elsewhere classified |
| Liver disease | K77 | Liver disorders in diseases classified elsewhere |
| Liver disease | K721 | Chronic hepatic failure |
| Liver disease | K74 | Fibrosis and cirrhosis of liver |
| Liver disease | K719 | Toxic liver disease, unspecified |
| Liver disease | K712 | Toxic liver disease with acute hepatitis |
| Liver disease | K73 | Chronic hepatitis, not elsewhere classified |
| Liver disease | K710 | Toxic liver disease with cholestasis |
| Liver disease | K713 | Toxic liver disease with chronic persistent hepatitis |
| Liver disease | K754 | Autoimmune hepatitis |
| Liver disease | K740 | Hepatic fibrosis |
| Prior bleed | I602 | Subarachnoid haemorrhage from anterior communicating artery |
| Prior bleed | I608 | Other subarachnoid haemorrhage |
| Prior bleed | S0651 | Traumatic subdural haemorrhage |
| Prior bleed | K762 | Central haemorrhagic necrosis of liver |
| Prior bleed | I604 | Subarachnoid haemorrhage from basilar artery |
| Prior bleed | S065 | Traumatic subdural haemorrhage |
| Prior bleed | N421 | Congestion and haemorrhage of prostate |
| Prior bleed | S0640 | Epidural haemorrhage |
| Prior bleed | H450 | Vitreous haemorrhage in diseases classified elsewhere |
| Prior bleed | I601 | Subarachnoid haemorrhage from middle cerebral artery |
| Prior bleed | N93 | Other abnormal uterine and vaginal bleeding |
| Prior bleed | N923 | Ovulation bleeding |
| Prior bleed | K250 | Gastric ulcer |
| Prior bleed | S064 | Epidural haemorrhage |
| Prior bleed | K921 | Melaena |
| Prior bleed | Y60 | Unintentional cut, puncture, perforation or haemorrhage during surgical and medical care |
| Prior bleed | I61 | Intracerebral haemorrhage |
| Prior bleed | S0660 | Traumatic subarachnoid haemorrhage |
| Prior bleed | I610 | Intracerebral haemorrhage in hemisphere, subcortical |
| Prior bleed | T810 | Haemorrhage and haematoma complicating a procedure, not elsewhere classified |
| Prior bleed | K226 | Gastro-oesophageal laceration-haemorrhage syndrome |
| Prior bleed | S0641 | Epidural haemorrhage |
| Prior bleed | I621 | Nontraumatic extradural haemorrhage |
| Prior bleed | R31X | Unspecified haematuria |
| Prior bleed | D698 | Other specified haemorrhagic conditions |
| Prior bleed | I603 | Subarachnoid haemorrhage from posterior communicating artery |
| Prior bleed | I618 | Other intracerebral haemorrhage |
| Prior bleed | H313 | Choroidal haemorrhage and rupture |
| Prior bleed | N950 | Postmenopausal bleeding |
| Prior bleed | I629 | Intracranial haemorrhage (nontraumatic), unspecified |
| Prior bleed | R048 | Haemorrhage from other sites in respiratory passages |
| Prior bleed | R041 | Haemorrhage from throat |
| Prior bleed | N028 | Recurrent and persistent haematuria |
| Prior bleed | H356 | Retinal haemorrhage |
| Prior bleed | I62 | Other nontraumatic intracranial haemorrhage |
| Prior bleed | I616 | Intracerebral haemorrhage, multiple localized |
| Prior bleed | R58X | Haemorrhage, not elsewhere classified |
| Prior bleed | K920 | Haematemesis |
| Prior bleed | I983 | Oesophageal varices with bleeding in diseases classified elsewhere |
| Prior bleed | I619 | Intracerebral haemorrhage, unspecified |
| Prior bleed | N930 | Postcoital and contact bleeding |
| Prior bleed | D62X | Acute posthaemorrhagic anaemia |
| Prior bleed | R049 | Haemorrhage from respiratory passages, unspecified |
| Prior bleed | K254 | Gastric ulcer |
| Prior bleed | K290 | Acute haemorrhagic gastritis |
| Prior bleed | N938 | Other specified abnormal uterine and vaginal bleeding |
| Prior bleed | I614 | Intracerebral haemorrhage in cerebellum |
| Prior bleed | I60 | Subarachnoid haemorrhage |
| Prior bleed | I600 | Subarachnoid haemorrhage from carotid siphon and bifurcation |
| Prior bleed | S0661 | Traumatic subarachnoid haemorrhage |
| Prior bleed | I606 | Subarachnoid haemorrhage from other intracranial arteries |
| Prior bleed | N029 | Recurrent and persistent haematuria |
| Prior bleed | I609 | Subarachnoid haemorrhage, unspecified |
| Prior bleed | S066 | Traumatic subarachnoid haemorrhage |
| Prior bleed | I613 | Intracerebral haemorrhage in brain stem |
| Prior bleed | K280 | Gastrojejunal ulcer |
| Prior bleed | J942 | Haemothorax |
| Prior bleed | K922 | Gastrointestinal haemorrhage, unspecified |
| Prior bleed | S0650 | Traumatic subdural haemorrhage |
| Prior bleed | I612 | Intracerebral haemorrhage in hemisphere, unspecified |
| Prior bleed | I615 | Intracerebral haemorrhage, intraventricular |
| Prior bleed | I607 | Subarachnoid haemorrhage from intracranial artery, unspecified |
| Prior bleed | H431 | Vitreous haemorrhage |
| Prior bleed | I850 | Oesophageal varices with bleeding |
| Prior bleed | I605 | Subarachnoid haemorrhage from vertebral artery |
| Prior bleed | K260 | Duodenal ulcer |
| Prior bleed | D699 | Haemorrhagic condition, unspecified |
| Prior bleed | K264 | Duodenal ulcer |
| Prior bleed | N924 | Excessive bleeding in the premenopausal period |
| Prior bleed | R04 | Haemorrhage from respiratory passages |
| Prior bleed | R042 | Haemoptysis |
| Prior bleed | K270 | Peptic ulcer, site unspecified |
| Prior bleed | I620 | Subdural haemorrhage (acute)(nontraumatic) |
| Prior bleed | T792 | Traumatic secondary and recurrent haemorrhage |
| Prior bleed | I611 | Intracerebral haemorrhage in hemisphere, cortical |
| Prior bleed | D683 | Haemorrhagic disorder due to circulating anticoagulants |
| Prior bleed | N939 | Abnormal uterine and vaginal bleeding, unspecified |
| Thromboembolism | I743 | Embolism and thrombosis of arteries of lower extremities |
| Thromboembolism | I742 | Embolism and thrombosis of arteries of upper extremities |
| Thromboembolism | I749 | Embolism and thrombosis of unspecified artery |
| Thromboembolism | I745 | Embolism and thrombosis of iliac artery |
| Thromboembolism | I741 | Embolism and thrombosis of other and unspecified parts of aorta |
| Thromboembolism | I74 | Arterial embolism and thrombosis |
| Thromboembolism | I748 | Embolism and thrombosis of other arteries |
| Thromboembolism | I744 | Embolism and thrombosis of arteries of extremities, unspecified |
| Thromboembolism | I740 | Embolism and thrombosis of abdominal aorta |
| Valvular AF | I052 | Mitral stenosis with insufficiency |
| Valvular AF | I059 | Mitral valve disease, unspecified |
| Valvular AF | I050 | Mitral stenosis |
| Valvular AF | I058 | Other mitral valve diseases |
| Valvular AF | I080 | Disorders of both mitral and aortic valves |
| Valvular AF | I089 | Multiple valve disease, unspecified |
| Valvular AF | Q230 | Congenital stenosis of aortic valve |
| Valvular AF | Q232 | Congenital mitral stenosis |
| Valvular AF | I083 | Combined disorders of mitral, aortic and tricuspid valves |
| Valvular AF | I081 | Disorders of both mitral and tricuspid valves |
| Valvular AF | I051 | Rheumatic mitral insufficiency |
| Valvular AF | Q233 | Congenital mitral insufficiency |
| Valvular AF | I342 | Nonrheumatic mitral (valve) stenosis |
| Valvular AF | Z952 | Presence of prosthetic heart valve |

Supplementary table 1.1. OPCS codes used to identify valvular AF

| NAME | CODE | DESCRIPTION |
| --- | --- | --- |
| Valvular AF | K251 | Allograft replacement of mitral valve |
| Valvular AF | K263 | Prosthetic replacement of aortic valve |
| Valvular AF | K253 | Prosthetic replacement of mitral valve |
| Valvular AF | K273 | Prosthetic replacement of tricuspid valve |
| Valvular AF | K264 | Replacement of aortic valve NEC |
| Valvular AF | K254 | Replacement of mitral valve NEC |
| Valvular AF | K252 | Xenograft replacement of mitral valve |

Supplementary table 1.3. Read codes used in the GP dataset to identify comorbidities, patient characteristics and exclusion criteria

| NAME | CODE | DESCRIPTION |
| --- | --- | --- |
| AF | G5730 | Atrial fibrillation |
| AF | 14AR. | History of atrial flutter |
| AF | G5732 | Paroxysmal atrial fibrillation |
| AF | G5739 | Atypical atrial flutter |
| AF | G573. | Atrial fibrillation and flutter |
| AF | G5736 | Paroxysmal atrial flutter |
| AF | G5731 | Atrial flutter |
| AF | G5737 | Chronic atrial fibrillation |
| AF | G573z | Atrial fibrillation and flutter NOS |
| AF | G5733 | Non-rheumatic atrial fibrillation |
| AF | 14AN. | H/O: atrial fibrillation |
| AF | G5735 | Persistent atrial fibrillation |
| AF | 8CMW2 | Atrial fibrillation care pathway |
| AF | G5734 | Permanent atrial fibrillation |
| AF | G5738 | Typical atrial flutter |
| AF | 3272. | ECG: atrial fibrillation |
| AF | 3273. | ECG: atrial flutter |
| DVT/PE | G801B | Deep vein thrombophlebitis of the leg unspecified |
| DVT/PE | G801F | Deep vein thrombosis of peroneal vein |
| DVT/PE | 14A8. | H/O: thrombo-embolism |
| DVT/PE | G801G | Recurrent deep vein thrombosis |
| DVT/PE | G801D | Deep vein thrombosis of lower limb |
| DVT/PE | 14A81 | H/O: Deep Vein Thrombosis |
| DVT/PE | G401. | Pulmonary embolism |
| DVT/PE | G801C | Deep vein thrombosis of leg related to air travel |
| DVT/PE | G801E | Deep vein thrombosis of leg related to intravenous drug use |
| DVT/PE | 14AC. | H/O: pulmonary embolus |
| DVT/PE | G4010 | Post operative pulmonary embolus |
| DVT/PE | G4011 | Recurrent pulmonary embolism |
| PVD | G73.. | Other peripheral vascular disease |
| PVD | G73z. | Peripheral vascular disease NOS |
| PVD | G73zz | Peripheral vascular disease NOS |
| PVD | G73y. | Other specified peripheral vascular disease |
| PVD | G73z0 | Intermittent claudication |
| PVD | G734. | Peripheral arterial disease |
| PVD | G700. | Aortic atherosclerosis |
| PVD | Gyu74 | [X]Other specified peripheral vascular diseases |
| Thyroid disease | C02yz | Thyrotoxicosis of other specified origin NOS |
| Thyroid disease | C043. | Other iatrogenic hypothyroidism |
| Thyroid disease | C0202 | Thyroid-associated dermopathy |
| Thyroid disease | C0241 | Thyrotoxicosis from ectopic thyroid nodule with crisis |
| Thyroid disease | C04z. | Hypothyroidism NOS |
| Thyroid disease | C02.. | Thyrotoxicosis |
| Thyroid disease | C0210 | Toxic uninodular goitre with no crisis |
| Thyroid disease | C022. | Toxic multinodular goitre |
| Thyroid disease | C02y. | Thyrotoxicosis of other specified origin |
| Thyroid disease | C02y1 | Thyrotoxicosis of other specified origin with crisis |
| Thyroid disease | C02z. | Thyrotoxicosis without mention of goitre or other cause |
| Thyroid disease | C04z0 | Premature puberty due to hypothyroidism |
| Thyroid disease | C02z1 | Thyrotoxicosis without mention of goitre, cause with crisis |
| Thyroid disease | C024z | Thyrotoxicosis from ectopic thyroid nodule NOS |
| Thyroid disease | C041z | Postablative hypothyroidism NOS |
| Thyroid disease | C02z0 | Thyrotoxicosis without mention of goitre or cause no crisis |
| Thyroid disease | C0211 | Toxic uninodular goitre with crisis |
| Thyroid disease | C0430 | Hypothyroidism resulting from para-aminosalicylic acid |
| Thyroid disease | C04y. | Other acquired hypothyroidism |
| Thyroid disease | C020z | Toxic diffuse goitre NOS |
| Thyroid disease | C046. | Autoimmune myxoedema |
| Thyroid disease | C02y3 | Thyroid crisis |
| Thyroid disease | C0200 | Toxic diffuse goitre with no crisis |
| Thyroid disease | C0221 | Toxic multinodular goitre with crisis |
| Thyroid disease | C02y0 | Thyrotoxicosis of other specified origin with no crisis |
| Thyroid disease | C0230 | Toxic nodular goitre unspecified with no crisis |
| Thyroid disease | C0240 | Thyrotoxicosis from ectopic thyroid nodule with no crisis |
| Thyroid disease | C045. | Acquired atrophy of thyroid |
| Thyroid disease | C022z | Toxic multinodular goitre NOS |
| Thyroid disease | C0410 | Irradiation hypothyroidism |
| Thyroid disease | C0201 | Toxic diffuse goitre with crisis |
| Thyroid disease | C04z1 | Myxoedema coma |
| Thyroid disease | C0231 | Toxic nodular goitre unspecified with crisis |
| Thyroid disease | C042. | Iodine hypothyroidism |
| Thyroid disease | C0220 | Toxic multinodular goitre with no crisis |
| Thyroid disease | C0431 | Hypothyroidism resulting from phenylbutazone |
| Thyroid disease | C04.. | Acquired hypothyroidism |
| Thyroid disease | C02zz | Thyrotoxicosis NOS |
| Thyroid disease | C020. | Toxic diffuse goitre |
| Thyroid disease | C025. | Subclinical hyperthyroidism |
| Thyroid disease | C023z | Toxic nodular goitre NOS |
| Thyroid disease | C021z | Toxic uninodular goitre NOS |
| Thyroid disease | C023. | Toxic nodular goitre unspecified |
| Thyroid disease | C043z | Iatrogenic hypothyroidism NOS |
| Thyroid disease | C044. | Postinfectious hypothyroidism |
| Thyroid disease | C047. | Subclinical hypothyroidism |
| Thyroid disease | C02y2 | Thyrotoxicosis factitia |
| Thyroid disease | C024. | Thyrotoxicosis from ectopic thyroid nodule |
| Thyroid disease | C0432 | Hypothyroidism resulting from resorcinol |
| Thyroid disease | C040. | Postsurgical hypothyroidism |
| Thyroid disease | C021. | Toxic uninodular goitre |
| Thyroid disease | C041. | Other postablative hypothyroidism |
| Excessive alcohol consumption | E230z | Acute alcoholic intoxication in alcoholism NOS |
| Excessive alcohol consumption | du23. | OPIZONE 50mg tablets |
| Excessive alcohol consumption | ZV113 | [V]Personal history of alcoholism |
| Excessive alcohol consumption | J611. | Acute alcoholic hepatitis |
| Excessive alcohol consumption | E2503 | Nondependent alcohol abuse in remission |
| Excessive alcohol consumption | du12. | ANTABUSE 200mg tablets |
| Excessive alcohol consumption | du1.. | DISULFIRAM |
| Excessive alcohol consumption | G555. | Alcoholic cardiomyopathy |
| Excessive alcohol consumption | 8IEA. | Referral to community alcohol team declined |
| Excessive alcohol consumption | du51. | ACAMPROSATE CALCIUM 333mg e/c tablets |
| Excessive alcohol consumption | du11. | DISULFIRAM 200mg tablets |
| Excessive alcohol consumption | E2312 | Episodic chronic alcoholism |
| Excessive alcohol consumption | J610. | Alcoholic fatty liver |
| Excessive alcohol consumption | 136S. | Hazardous alcohol use |
| Excessive alcohol consumption | G8523 | Oesophageal varices in alcoholic cirrhosis of the liver |
| Excessive alcohol consumption | F25B. | Alcohol-induced epilepsy |
| Excessive alcohol consumption | 9k19. | Alcohol assesment declined - enhanced services admin |
| Excessive alcohol consumption | E231z | Chronic alcoholism NOS |
| Excessive alcohol consumption | 8W2.. | Refer to MH services deferred until alcohol misuse resolved |
| Excessive alcohol consumption | J6130 | Alcoholic hepatic failure |
| Excessive alcohol consumption | 1B1c. | Alcohol induced hallucinations |
| Excessive alcohol consumption | 9k11. | Alcohol consumption counselling |
| Excessive alcohol consumption | du2.. | NALTREXONE HYDROCHLORIDE |
| Excessive alcohol consumption | 9NJz. | In-house alcohol detoxification |
| Excessive alcohol consumption | E2311 | Continuous chronic alcoholism |
| Excessive alcohol consumption | E2500 | Nondependent alcohol abuse, unspecified |
| Excessive alcohol consumption | 9k12. | Alcohol misuse - enhanced service completed |
| Excessive alcohol consumption | J612. | Alcoholic cirrhosis of liver |
| Excessive alcohol consumption | 8H7p. | Referral to community alcohol team |
| Excessive alcohol consumption | F1440 | Cerebellar ataxia due to alcoholism |
| Excessive alcohol consumption | 66e.. | Alcohol disorder monitoring |
| Excessive alcohol consumption | ZV6D6 | [V]Alcohol abuse counselling and surveillance |
| Excessive alcohol consumption | J6710 | Alcohol-induced chronic pancreatitis |
| Excessive alcohol consumption | C1505 | Alcohol-induced pseudo-Cushing's syndrome |
| Excessive alcohol consumption | 8G32. | Aversion therapy - alcoholism |
| Excessive alcohol consumption | ZV57A | [V]Alcohol rehabilitation |
| Excessive alcohol consumption | J613. | Alcoholic liver damage unspecified |
| Excessive alcohol consumption | 8HkJ. | Referral to alcohol brief intervention service |
| Excessive alcohol consumption | 8CAv. | Advised to contact primary care alcohol worker |
| Excessive alcohol consumption | 136T. | Harmful alcohol use |
| Excessive alcohol consumption | 1462. | H/O: alcoholism |
| Excessive alcohol consumption | du5.. | ACAMPROSATE CALCIUM |
| Excessive alcohol consumption | 38Dz. | Severity of alcohol dependence questionnaire |
| Excessive alcohol consumption | F3941 | Alcoholic myopathy |
| Excessive alcohol consumption | 9k1.. | Alcohol misuse - enhanced services administration |
| Excessive alcohol consumption | E2300 | Acute alcoholic intoxication, unspecified, in alcoholism |
| Excessive alcohol consumption | E2313 | Chronic alcoholism in remission |
| Excessive alcohol consumption | du22. | NALOREX 50mg tablets |
| Excessive alcohol consumption | 8IAt. | Extended interven for excessive alcohol consumption declined |
| Excessive alcohol consumption | J153. | Alcoholic gastritis |
| Excessive alcohol consumption | E230. | Acute alcoholic intoxication in alcoholism |
| Excessive alcohol consumption | du21. | NALTREXONE HYDROCHLORIDE 50mg tablets |
| Excessive alcohol consumption | 8BA8. | Alcohol detoxification |
| Excessive alcohol consumption | 9k1B. | Extended intervention for excessive alcohol consumptn complt |
| Excessive alcohol consumption | E2310 | Unspecified chronic alcoholism |
| Excessive alcohol consumption | E2303 | Acute alcoholic intoxication in remission, in alcoholism |
| Excessive alcohol consumption | du24. | ADEPEND 50mg tablets |
| Excessive alcohol consumption | E250z | Nondependent alcohol abuse NOS |
| Excessive alcohol consumption | 7P221 | Delivery of rehabilitation for alcohol addiction |
| Excessive alcohol consumption | E2501 | Nondependent alcohol abuse, continuous |
| Excessive alcohol consumption | 9NN2. | Under care of community alcohol team |
| Excessive alcohol consumption | E2502 | Nondependent alcohol abuse, episodic |
| Excessive alcohol consumption | 66e0. | Alcohol abuse monitoring |
| Excessive alcohol consumption | 136W. | Alcohol misuse |
| Excessive alcohol consumption | E23z. | Alcohol dependence syndrome NOS |
| Excessive alcohol consumption | 9k10. | Community detoxification registered |
| Excessive alcohol consumption | F11x0 | Cerebral degeneration due to alcoholism |
| Excessive alcohol consumption | J6170 | Chronic alcoholic hepatitis |
| Excessive alcohol consumption | E2302 | Episodic acute alcoholic intoxication in alcoholism |
| Excessive alcohol consumption | 136R. | Binge drinker |
| Excessive alcohol consumption | 136Q. | Very heavy drinker |
| Excessive alcohol consumption | 1366. | Very heavy drinker - >9u/day |
| Excessive alcohol consumption | J6120 | Alcoholic fibrosis and sclerosis of liver |
| Excessive alcohol consumption | E2301 | Continuous acute alcoholic intoxication in alcoholism |
| Excessive alcohol consumption | 9k1A. | Brief intervention for excessive alcohol consumptn completed |
| Excessive alcohol consumption | du52. | CAMPRAL EC 333mg e/c tablets |
| Excessive alcohol consumption | 9k14. | Alcohol counselling by other agencies |
| Excessive alcohol consumption | E231. | Chronic alcoholism |
| Excessive alcohol consumption | 8H35. | Admitted to alcohol detoxification centre |
| Excessive alcohol consumption | E250. | Nondependent alcohol abuse |
| Excessive alcohol consumption | E23.. | Alcohol dependence syndrome |
| Excessive alcohol consumption | 8IAJ. | Declined referral to specialist alcohol treatment service |
| Excessive alcohol consumption | 8IAF. | Brief intervention for excessive alcohol consumptn declined |
| Excessive alcohol consumption | 8HkG. | Referral to specialist alcohol treatment service |
| Excessive alcohol consumption | J6708 | Alcohol-induced acute pancreatitis |
| Excessive alcohol consumption | F375. | Alcoholic polyneuropathy |
| Excessive alcohol consumption | J617. | Alcoholic hepatitis |
| Cancer | B5450 | Malignant neoplasm of glomus jugulare |
| Cancer | B6004 | Reticulosarcoma of lymph nodes of axilla and upper limb |
| Cancer | B082. | Malignant neoplasm aryepiglottic fold, hypopharyngeal aspect |
| Cancer | B620z | Nodular lymphoma NOS |
| Cancer | B3125 | Malignant neoplasm of connective and soft tissue of toe |
| Cancer | B315. | Malignant neoplasm of connective and soft tissue of pelvis |
| Cancer | B080. | Malignant neoplasm of postcricoid region |
| Cancer | B0600 | Malignant neoplasm of faucial tonsil |
| Cancer | B1z.. | Malig neop oth/ill-defined sites digestive tract/peritoneum |
| Cancer | B680. | Acute leukaemia NOS |
| Cancer | B304. | Malignant neoplasm of scapula and long bones of upper arm |
| Cancer | B022. | Malignant neoplasm of sublingual gland |
| Cancer | B542. | Malignant neoplasm pituitary gland and craniopharyngeal duct |
| Cancer | B6236 | Malignant histiocytosis of intrapelvic lymph nodes |
| Cancer | B5201 | Malignant neoplasm of optic nerve |
| Cancer | ByuD1 | [X]Other types of follicular non-Hodgkin's lymphoma |
| Cancer | B2000 | Malignant neoplasm of cartilage of nose |
| Cancer | B3133 | Malig neoplasm of connective and soft tissues of thor spine |
| Cancer | B0510 | Malignant neoplasm of upper buccal sulcus |
| Cancer | B3331 | Malignant neoplasm of skin of chin |
| Cancer | B5121 | Malignant neoplasm of uncus |
| Cancer | B0512 | Malignant neoplasm of upper labial sulcus |
| Cancer | B615z | Hodgkin's disease, mixed cellularity NOS |
| Cancer | B6167 | Hodgkin's disease, lymphocytic depletion of spleen |
| Cancer | B308A | Malignant neoplasm of third metatarsal bone |
| Cancer | B6123 | Hodgkin's sarcoma of intra-abdominal lymph nodes |
| Cancer | ZV101 | [V]Personal history of malig neop of trachea/bronchus/lung |
| Cancer | B04.. | Malignant neoplasm of floor of mouth |
| Cancer | B014. | Malignant neoplasm of anterior 2/3 of tongue unspecified |
| Cancer | B660. | Acute monocytic leukaemia |
| Cancer | B55y2 | Malignant neoplasm of flank NOS |
| Cancer | B0620 | Malignant neoplasm of faucial pillar |
| Cancer | B510. | Malignant neoplasm cerebrum (excluding lobes and ventricles) |
| Cancer | B311z | Malig neop connective soft tissue upper limb/shoulder NOS |
| Cancer | B23.. | Malignant neoplasm of pleura |
| Cancer | B4A4. | Malignant neoplasm of paraurethral glands |
| Cancer | B512z | Malignant neoplasm of temporal lobe NOS |
| Cancer | B3351 | Malignant neoplasm of skin of chest, excluding breast |
| Cancer | B495. | Malignant neoplasm of bladder neck |
| Cancer | B2011 | Malignant neoplasm of tympanic cavity |
| Cancer | B11y1 | Malignant neoplasm of posterior wall of stomach NEC |
| Cancer | B0zy. | Malignant neoplasm of other sites lip, oral cavity, pharynx |
| Cancer | B6153 | Hodgkin's mixed cellularity of intra-abdominal lymph nodes |
| Cancer | B64z. | Lymphoid leukaemia NOS |
| Cancer | B308D | Malignant neoplasm of phalanges of foot |
| Cancer | B6005 | Reticulosarcoma of lymph nodes of inguinal region and leg |
| Cancer | B622. | Sezary's disease |
| Cancer | B6208 | Nodular lymphoma of lymph nodes of multiple sites |
| Cancer | B3122 | Malig neop connective and soft tissue of popliteal space |
| Cancer | B150. | Primary malignant neoplasm of liver |
| Cancer | B6022 | Burkitt's lymphoma of intrathoracic lymph nodes |
| Cancer | B6103 | Hodgkin's paragranuloma of intra-abdominal lymph nodes |
| Cancer | B42.. | Malignant neoplasm of placenta |
| Cancer | B6303 | Lambda light chain myeloma |
| Cancer | B48y. | Malignant neoplasm of other male genital organ |
| Cancer | B62y. | Malignant lymphoma NOS |
| Cancer | B12.. | Malignant neoplasm of small intestine and duodenum |
| Cancer | B6006 | Reticulosarcoma of intrapelvic lymph nodes |
| Cancer | B63y. | Other immunoproliferative neoplasms |
| Cancer | B6277 | Diffuse non-Hodgkin's lymphoblastic (diffuse) lymphoma |
| Cancer | B430z | Malignant neoplasm of corpus uteri NOS |
| Cancer | B61z7 | Hodgkin's disease NOS of spleen |
| Cancer | B6137 | Hodgkin's, lymphocytic-histiocytic predominance of spleen |
| Cancer | Byu43 | [X]Malignant neoplasm of skin, unspecified |
| Cancer | B61z2 | Hodgkin's disease NOS of intrathoracic lymph nodes |
| Cancer | B6020 | Burkitt's lymphoma of unspecified site |
| Cancer | B6203 | Nodular lymphoma of intra-abdominal lymph nodes |
| Cancer | B0022 | Malignant neoplasm of upper lip, mucosa |
| Cancer | B48z. | Malignant neoplasm of penis and other male genital organ NOS |
| Cancer | B3278 | Malignant melanoma of toe |
| Cancer | B45y. | Malignant neoplasm of other specified female genital organ |
| Cancer | B17y. | Malignant neoplasm of other specified sites of pancreas |
| Cancer | B52W. | Malig neopl, overlap lesion brain & other part of CNS |
| Cancer | B225. | Malignant neoplasm of overlapping lesion of bronchus & lung |
| Cancer | B552. | Malignant neoplasm of abdomen |
| Cancer | B480. | Malignant neoplasm of prepuce (foreskin) |
| Cancer | B61z1 | Hodgkin's disease NOS of lymph nodes of head, face and neck |
| Cancer | B242. | Malignant neoplasm of anterior mediastinum |
| Cancer | B67y0 | Lymphosarcoma cell leukaemia |
| Cancer | B6302 | Plasmacytoma NOS |
| Cancer | B3020 | Malignant neoplasm of cervical vertebra |
| Cancer | B41y0 | Malignant neoplasm of cervical stump |
| Cancer | ZV10y | [V]Personal history of other specified malignant neoplasm |
| Cancer | B221. | Malignant neoplasm of main bronchus |
| Cancer | B040. | Malignant neoplasm of anterior portion of floor of mouth |
| Cancer | B105. | Malignant neoplasm of lower third of oesophagus |
| Cancer | B331. | Malignant neoplasm of eyelid including canthus |
| Cancer | B2010 | Malignant neoplasm of auditory (Eustachian) tube |
| Cancer | B3221 | Malignant melanoma of external auditory meatus |
| Cancer | B6z0. | Kaposi's sarcoma of lymph nodes |
| Cancer | B4510 | Malignant neoplasm of greater vestibular (Bartholin's) gland |
| Cancer | B3050 | Malignant neoplasm of carpal bone - scaphoid |
| Cancer | B18y0 | Malignant neoplasm of mesocolon |
| Cancer | B110. | Malignant neoplasm of cardia of stomach |
| Cancer | B0711 | Malignant neoplasm of pharyngeal tonsil |
| Cancer | B050. | Malignant neoplasm of cheek mucosa |
| Cancer | B06z. | Malignant neoplasm of oropharynx NOS |
| Cancer | B6226 | Sezary's disease of intrapelvic lymph nodes |
| Cancer | B3061 | Malignant neoplasm of ischium |
| Cancer | B3030 | Malignant neoplasm of rib |
| Cancer | B62z3 | Unspec malig neop lymphoid/histiocytic intra-abdominal nodes |
| Cancer | B5211 | Malignant neoplasm of cerebral arachnoid mater |
| Cancer | B3082 | Malignant neoplasm of calcaneum |
| Cancer | B300A | Malignant neoplasm of maxilla |
| Cancer | B5240 | Malignant neoplasm of peripheral nerves of head, face & neck |
| Cancer | B3081 | Malignant neoplasm of talus |
| Cancer | B3376 | Malignant neoplasm of skin of heel |
| Cancer | B334z | Malignant neoplasm of scalp or skin of neck NOS |
| Cancer | B180. | Malignant neoplasm of retroperitoneum |
| Cancer | B471. | Malignant neoplasm of descended testis |
| Cancer | B692. | Subacute myelomonocytic leukaemia |
| Cancer | B220z | Malignant neoplasm of trachea NOS |
| Cancer | B0732 | Malignant neoplasm posterior margin nasal septum and choanae |
| Cancer | B3500 | Malignant neoplasm of nipple of male breast |
| Cancer | B3062 | Malignant neoplasm of pubis |
| Cancer | B51z. | Malignant neoplasm of brain NOS |
| Cancer | B115. | Malignant neoplasm of lesser curve of stomach unspecified |
| Cancer | B0010 | Malignant neoplasm of lower lip, external |
| Cancer | B50.. | Malignant neoplasm of eye |
| Cancer | B5210 | Malignant neoplasm of cerebral dura mater |
| Cancer | B20.. | Malig neop nasal cavities, middle ear and accessory sinuses |
| Cancer | B1z2. | Malignant neoplasm, overlapping lesion of digestive system |
| Cancer | B3359 | Malignant neoplasm of perianal skin |
| Cancer | B015. | Malignant neoplasm of tongue, junctional zone |
| Cancer | B611z | Hodgkin's granuloma NOS |
| Cancer | B544. | Malignant neoplasm of carotid body |
| Cancer | B04y. | Malignant neoplasm of other sites of floor of mouth |
| Cancer | B07z. | Malignant neoplasm of nasopharynx NOS |
| Cancer | Bz... | Neoplasms NOS |
| Cancer | B5170 | Malignant neoplasm of cerebral peduncle |
| Cancer | B057. | Overlapping lesion of other and unspecified parts of mouth |
| Cancer | Byu33 | [X]Malignant neoplasm/bone+articular cartilage, unspecified |
| Cancer | Byu72 | [X]Malignant neoplasm/overlapping lesion/feml genital organs |
| Cancer | B541. | Malignant neoplasm of parathyroid gland |
| Cancer | B104. | Malignant neoplasm of middle third of oesophagus |
| Cancer | B6121 | Hodgkin's sarcoma of lymph nodes of head, face and neck |
| Cancer | B432. | Malignant neoplasm of overlapping lesion of corpus uteri |
| Cancer | B200. | Malignant neoplasm of nasal cavities |
| Cancer | ByuD8 | [X]Other specified leukaemias |
| Cancer | B6227 | Sezary's disease of spleen |
| Cancer | B411. | Malignant neoplasm of exocervix |
| Cancer | B6143 | Hodgkin's nodular sclerosis of intra-abdominal lymph nodes |
| Cancer | B22y. | Malignant neoplasm of other sites of bronchus or lung |
| Cancer | B6028 | Burkitt's lymphoma of lymph nodes of multiple sites |
| Cancer | B6026 | Burkitt's lymphoma of intrapelvic lymph nodes |
| Cancer | B600. | Reticulosarcoma |
| Cancer | B021. | Malignant neoplasm of submandibular gland |
| Cancer | B222. | Malignant neoplasm of upper lobe, bronchus or lung |
| Cancer | B4301 | Malignant neoplasm of fundus of corpus uteri |
| Cancer | B542z | Malig neop pituitary gland or craniopharyngeal duct NOS |
| Cancer | B6255 | Letterer-Siwe disease of lymph nodes inguinal region and leg |
| Cancer | B346. | Malignant neoplasm of axillary tail of female breast |
| Cancer | B241z | Malignant neoplasm of heart NOS |
| Cancer | B0513 | Malignant neoplasm of lower labial sulcus |
| Cancer | B1512 | Malignant neoplasm of intrahepatic biliary passages |
| Cancer | B43y. | Malignant neoplasm of other site of uterine body |
| Cancer | B004z | Malignant neoplasm of lip, inner aspect NOS |
| Cancer | B682. | Subacute leukaemia NOS |
| Cancer | B180z | Malignant neoplasm of retroperitoneum NOS |
| Cancer | B4A1. | Malignant neoplasm of renal pelvis |
| Cancer | B51y1 | Malignant neoplasm of tapetum |
| Cancer | B68.. | Leukaemia of unspecified cell type |
| Cancer | B470. | Malignant neoplasm of undescended testis |
| Cancer | Byu12 | [X]Malignant neoplasm of intestinal tract, part unspecified |
| Cancer | B51.. | Malignant neoplasm of brain |
| Cancer | B2013 | Malignant neoplasm of mastoid air cells |
| Cancer | B64yz | Other lymphoid leukaemia NOS |
| Cancer | B62x6 | True histiocytic lymphoma |
| Cancer | B3051 | Malignant neoplasm of carpal bone - lunate |
| Cancer | B202. | Malignant neoplasm of maxillary sinus |
| Cancer | B1610 | Malignant neoplasm of cystic duct |
| Cancer | B5421 | Malignant neoplasm of craniopharyngeal duct |
| Cancer | B48y2 | Malignant neoplasm, overlapping lesion male genital orgs |
| Cancer | B5244 | Malignant neoplasm of peripheral nerve of abdomen |
| Cancer | B06.. | Malignant neoplasm of oropharynx |
| Cancer | B316. | Malig neop of connective and soft tissue trunk unspecified |
| Cancer | B0110 | Malignant neoplasm of anterior 2/3 of tongue dorsal surface |
| Cancer | B3311 | Malignant neoplasm of upper eyelid |
| Cancer | B6100 | Hodgkin's paragranuloma of unspecified site |
| Cancer | B3132 | Malignant neoplasm of great vessels |
| Cancer | B4101 | Malignant neoplasm of endocervical gland |
| Cancer | B610z | Hodgkin's paragranuloma NOS |
| Cancer | B101. | Malignant neoplasm of thoracic oesophagus |
| Cancer | B24X. | Malignant neoplasm of mediastinum, part unspecified |
| Cancer | B6025 | Burkitt's lymphoma of lymph nodes of inguinal region and leg |
| Cancer | B051. | Malignant neoplasm of vestibule of mouth |
| Cancer | B1z0. | Malignant neoplasm of intestinal tract, part unspecified |
| Cancer | B62z2 | Unspec malig neop lymphoid/histiocytic of intrathoracic node |
| Cancer | B44z. | Malignant neoplasm of uterine adnexa NOS |
| Cancer | B055z | Malignant neoplasm of palate NOS |
| Cancer | B60.. | Lymphosarcoma and reticulosarcoma |
| Cancer | B6201 | Nodular lymphoma of lymph nodes of head, face and neck |
| Cancer | B001z | Malignant neoplasm of lower lip, vermilion border NOS |
| Cancer | B212. | Malignant neoplasm of subglottis |
| Cancer | B064. | Malignant neoplasm of anterior epiglottis |
| Cancer | B3021 | Malignant neoplasm of thoracic vertebra |
| Cancer | B62y4 | Malignant lymphoma NOS of lymph nodes of axilla and arm |
| Cancer | B344. | Malignant neoplasm of upper-outer quadrant of female breast |
| Cancer | B0040 | Malignant neoplasm of lip unspecified, buccal aspect |
| Cancer | B170. | Malignant neoplasm of head of pancreas |
| Cancer | B52y. | Malignant neoplasm of other specified part of nervous system |
| Cancer | B1613 | Malignant neoplasm of sphincter of Oddi |
| Cancer | B34y0 | Malignant neoplasm of ectopic site of female breast |
| Cancer | B3033 | Malignant neoplasm of costal cartilage |
| Cancer | B2133 | Malignant neoplasm of thyroid cartilage |
| Cancer | B551z | Malignant neoplasm of thorax NOS |
| Cancer | ByuDD | [X]Oth and unspecif peripheral & cutaneous T-cell lymphomas |
| Cancer | B162. | Malignant neoplasm of ampulla of Vater |
| Cancer | B55yz | Malignant neoplasm of specified site NOS |
| Cancer | B6530 | Chloroma |
| Cancer | B6101 | Hodgkin's paragranuloma of lymph nodes of head, face, neck |
| Cancer | B5501 | Malignant neoplasm of cheek NOS |
| Cancer | B33y. | Malignant neoplasm of other specified skin sites |
| Cancer | B0730 | Malignant neoplasm of floor of nasopharynx |
| Cancer | B3058 | Malignant neoplasm of first metacarpal bone |
| Cancer | B3141 | Malig neoplasm of connective and soft tissues of lumb spine |
| Cancer | B6223 | Sezary's disease of intra-abdominal lymph nodes |
| Cancer | B31z. | Malignant neoplasm of connective and soft tissue, site NOS |
| Cancer | B4302 | Malignant neoplasm of endometrium of corpus uteri |
| Cancer | Byu73 | [X]Malignant neoplasm of female genital organ, unspecified |
| Cancer | B3501 | Malignant neoplasm of areola of male breast |
| Cancer | B410z | Malignant neoplasm of endocervix NOS |
| Cancer | B505. | Malignant neoplasm of retina |
| Cancer | B6218 | Mycosis fungoides of lymph nodes of multiple sites |
| Cancer | B2001 | Malignant neoplasm of nasal conchae |
| Cancer | B2211 | Malignant neoplasm of hilus of lung |
| Cancer | B454. | Malignant neoplasm of vulva unspecified |
| Cancer | B6263 | Mast cell malignancy of intra-abdominal lymph nodes |
| Cancer | B0622 | Malignant neoplasm of palatoglossal arch |
| Cancer | B627D | Diffuse non-Hodgkin's centroblastic lymphoma |
| Cancer | B20z. | Malignant neoplasm of accessory sinus NOS |
| Cancer | B5231 | Malignant neoplasm of spinal arachnoid mater |
| Cancer | B3087 | Malignant neoplasm of navicular |
| Cancer | B3140 | Malig neop of connective and soft tissue of abdominal wall |
| Cancer | B3355 | Malignant neoplasm of skin of groin |
| Cancer | B640. | Acute lymphoid leukaemia |
| Cancer | B6232 | Malignant histiocytosis of intrathoracic lymph nodes |
| Cancer | B65y1 | Acute promyelocytic leukaemia |
| Cancer | B000. | Malignant neoplasm of upper lip, vermilion border |
| Cancer | B5511 | Malignant neoplasm of chest wall NOS |
| Cancer | By... | Neoplasms otherwise specified |
| Cancer | B55y. | Malignant neoplasm of other specified sites |
| Cancer | B45X. | Malignant neoplasm/overlapping lesion/feml genital organs |
| Cancer | B3260 | Malignant melanoma of shoulder |
| Cancer | B5503 | Malignant neoplasm of jaw NOS |
| Cancer | ZV12C | [V] Personal history of gastric ulcer |
| Cancer | B3220 | Malignant melanoma of auricle (ear) |
| Cancer | Byu0. | [X]Malignant neoplasm of lip, oral cavity and pharynx |
| Cancer | B0551 | Malignant neoplasm of roof of mouth |
| Cancer | B5071 | Malignant neoplasm of nasolacrimal duct |
| Cancer | B61.. | Hodgkin's disease |
| Cancer | B3022 | Malignant neoplasm of lumbar vertebra |
| Cancer | B4A00 | Hypernephroma |
| Cancer | B2... | Malig neop of respiratory tract and intrathoracic organs |
| Cancer | B3234 | Malignant melanoma of external surface of nose |
| Cancer | B5011 | Malignant neoplasm of extraocular muscle of orbit |
| Cancer | B3031 | Malignant neoplasm of sternum |
| Cancer | B6258 | Letterer-Siwe disease of lymph nodes of multiple sites |
| Cancer | B16y. | Malignant neoplasm other gallbladder/extrahepatic bile duct |
| Cancer | B453. | Malignant neoplasm of clitoris |
| Cancer | ZV104 | [V]Personal history of malignant neoplasm of genital organ |
| Cancer | B066. | Malignant neoplasm of lateral wall of oropharynx |
| Cancer | B335. | Malignant neoplasm of skin of trunk, excluding scrotum |
| Cancer | ByuD. | [X]Malignant neoplasms of lymphoid, haematopoietic and rela |
| Cancer | B551. | Malignant neoplasm of thorax |
| Cancer | B5070 | Malignant neoplasm of lacrimal sac |
| Cancer | B0601 | Malignant neoplasm of palatine tonsil |
| Cancer | B011z | Malignant neoplasm of dorsum of tongue NOS |
| Cancer | B6142 | Hodgkin's nodular sclerosis of intrathoracic lymph nodes |
| Cancer | B6240 | Leukaemic reticuloendotheliosis of unspecified sites |
| Cancer | B14z. | Malignant neoplasm rectum,rectosigmoid junction and anus NOS |
| Cancer | B3279 | Malignant melanoma of great toe |
| Cancer | B517. | Malignant neoplasm of brain stem |
| Cancer | B67z. | Other specified leukaemia NOS |
| Cancer | B201z | Malig neop auditory tube, middle ear, mastoid air cells NOS |
| Cancer | B3115 | Malignant neoplasm of connective and soft tissue of thumb |
| Cancer | B624. | Leukaemic reticuloendotheliosis |
| Cancer | B3364 | Malignant neoplasm of skin of finger |
| Cancer | B210. | Malignant neoplasm of glottis |
| Cancer | B3005 | Malignant neoplasm of orbital bone |
| Cancer | B060. | Malignant neoplasm of tonsil |
| Cancer | Byu57 | [X]Malignant neoplasm of peritoneum, unspecified |
| Cancer | B490. | Malignant neoplasm of trigone of urinary bladder |
| Cancer | B21.. | Malignant neoplasm of larynx |
| Cancer | B6126 | Hodgkin's sarcoma of intrapelvic lymph nodes |
| Cancer | B333. | Malignant neoplasm skin of other and unspecified parts face |
| Cancer | B13y. | Malignant neoplasm of other specified sites of colon |
| Cancer | B6127 | Hodgkin's sarcoma of spleen |
| Cancer | B33z0 | Kaposi's sarcoma of skin |
| Cancer | B45.. | Malig neop of other and unspecified female genital organs |
| Cancer | B307z | Malignant neoplasm of long bones of leg NOS |
| Cancer | B3104 | Malignant neoplasm of tarsus of eyelid |
| Cancer | B6010 | Lymphosarcoma of unspecified site |
| Cancer | B5245 | Malignant neoplasm of peripheral nerve of pelvis |
| Cancer | B304z | Malig neop of scapula and long bones of upper arm NOS |
| Cancer | B61z4 | Hodgkin's disease NOS of lymph nodes of axilla and arm |
| Cancer | B6141 | Hodgkin's nodular sclerosis of head, face and neck |
| Cancer | B41.. | Malignant neoplasm of cervix uteri |
| Cancer | Byu30 | [X]Mal neoplasm/overlap lesion/bone+articular cartilage/limb |
| Cancer | B601. | Lymphosarcoma |
| Cancer | Byu9. | [X]Malignant neoplasm of urinary tract |
| Cancer | B003. | Malignant neoplasm of lower lip, inner aspect |
| Cancer | B3130 | Malignant neoplasm of connective and soft tissue of axilla |
| Cancer | B336z | Malignant neoplasm of skin of upper limb or shoulder NOS |
| Cancer | B06yz | Malignant neoplasm of other specified site of oropharynx NOS |
| Cancer | B137. | Malignant neoplasm of splenic flexure of colon |
| Cancer | B5532 | Malignant neoplasm of sacrococcygeal region |
| Cancer | B2411 | Malignant neoplasm of epicardium |
| Cancer | B18y4 | Malignant neoplasm of parietal peritoneum |
| Cancer | B142. | Malignant neoplasm of anal canal |
| Cancer | B6248 | Leukaemic reticuloend of lymph nodes of multiple sites |
| Cancer | B3150 | Malignant neoplasm of connective and soft tissue of buttock |
| Cancer | B017. | Malignant overlapping lesion of tongue |
| Cancer | B626z | Malignant mast cell tumour NOS |
| Cancer | B116. | Malignant neoplasm of greater curve of stomach unspecified |
| Cancer | B6244 | Leukaemic reticuloend of lymph nodes of axilla and arm |
| Cancer | B6237 | Malignant histiocytosis of spleen |
| Cancer | B622z | Sezary's disease NOS |
| Cancer | B3265 | Malignant melanoma of thumb |
| Cancer | B6011 | Lymphosarcoma of lymph nodes of head, face and neck |
| Cancer | B3121 | Malig neop of connective and soft tissue thigh and upper leg |
| Cancer | B3263 | Malignant melanoma of hand |
| Cancer | B3032 | Malignant neoplasm of clavicle |
| Cancer | B042. | Malignant neoplasm, overlapping lesion of floor of mouth |
| Cancer | B6115 | Hodgkin's granuloma lymph nodes of inguinal region and leg |
| Cancer | B02y. | Malignant neoplasm of other major salivary glands |
| Cancer | B16.. | Malignant neoplasm gallbladder and extrahepatic bile ducts |
| Cancer | B431z | Malignant neoplasm of isthmus of uterine body NOS |
| Cancer | B6108 | Hodgkin's paragranuloma of lymph nodes of multiple sites |
| Cancer | B1z10 | Angiosarcoma of spleen |
| Cancer | B231. | Malignant neoplasm of visceral pleura |
| Cancer | B3009 | Malignant neoplasm of zygomatic bone |
| Cancer | B3065 | Malignant sacral teratoma |
| Cancer | B0710 | Malignant neoplasm of adenoid |
| Cancer | B0020 | Malignant neoplasm of upper lip, buccal aspect |
| Cancer | B62.. | Other malignant neoplasm of lymphoid and histiocytic tissue |
| Cancer | B6148 | Hodgkin's nodular sclerosis of lymph nodes of multiple sites |
| Cancer | B503. | Malignant neoplasm of conjunctiva |
| Cancer | B3003 | Malignant neoplasm of nasal bone |
| Cancer | B6222 | Sezary's disease of intrathoracic lymph nodes |
| Cancer | B31z0 | Kaposi's sarcoma of soft tissue |
| Cancer | B325z | Malignant melanoma of trunk, excluding scrotum, NOS |
| Cancer | B48yz | Malignant neoplasm of other male genital organ NOS |
| Cancer | B341. | Malignant neoplasm of central part of female breast |
| Cancer | B621. | Mycosis fungoides |
| Cancer | ByuA. | [X]Malignant neoplasm of eye, brain and other parts of cent |
| Cancer | B32.. | Malignant melanoma of skin |
| Cancer | B45z. | Malignant neoplasm of female genital organ NOS |
| Cancer | B0623 | Malignant neoplasm of palatopharyngeal arch |
| Cancer | B062z | Malignant neoplasm of tonsillar fossa NOS |
| Cancer | B313z | Malig neop of connective and soft tissue of thorax NOS |
| Cancer | B6268 | Mast cell malignancy of lymph nodes of multiple sites |
| Cancer | B305C | Malignant neoplasm of fifth metacarpal bone |
| Cancer | B24y. | Malig neop of other site of heart, thymus and mediastinum |
| Cancer | B3057 | Malignant neoplasm of carpal bone - hamate |
| Cancer | Byu71 | [X]Malignant neoplasm/other specified female genital organs |
| Cancer | B160. | Malignant neoplasm of gallbladder |
| Cancer | Byu59 | [X]Malignant neoplasm/connective + soft tissue,unspecified |
| Cancer | B3004 | Malignant neoplasm of occipital bone |
| Cancer | B32y. | Malignant melanoma of other specified skin site |
| Cancer | B62y6 | Malignant lymphoma NOS of intrapelvic lymph nodes |
| Cancer | B6z.. | Malignant neoplasm lymphatic or haematopoietic tissue NOS |
| Cancer | B220. | Malignant neoplasm of trachea |
| Cancer | B441. | Malignant neoplasm of fallopian tube |
| Cancer | B41z. | Malignant neoplasm of cervix uteri NOS |
| Cancer | B3054 | Malignant neoplasm of carpal bone - trapezium |
| Cancer | B223z | Malignant neoplasm of middle lobe, bronchus or lung NOS |
| Cancer | B501z | Malignant neoplasm of orbit NOS |
| Cancer | B3102 | Malignant neoplasm of soft tissue of neck |
| Cancer | B307. | Malignant neoplasm of long bones of leg |
| Cancer | B6105 | Hodgkin's paragranuloma lymph nodes inguinal region and leg |
| Cancer | B62yz | Malignant lymphoma NOS |
| Cancer | ByuD7 | [X]Other monocytic leukaemia |
| Cancer | B3080 | Malignant neoplasm of patella |
| Cancer | B35zz | Malignant neoplasm of male breast NOS |
| Cancer | B616z | Hodgkin's disease, lymphocytic depletion NOS |
| Cancer | B1z1z | Malignant neoplasm of spleen NOS |
| Cancer | B2220 | Malignant neoplasm of upper lobe bronchus |
| Cancer | B614z | Hodgkin's disease, nodular sclerosis NOS |
| Cancer | B550. | Malignant neoplasm of head, neck and face |
| Cancer | B305z | Malignant neoplasm of hand bones NOS |
| Cancer | B6301 | Solitary myeloma |
| Cancer | B6273 | Diffuse non-Hodgkin's small cell (diffuse) lymphoma |
| Cancer | B6104 | Hodgkin's paragranuloma of lymph nodes of axilla and arm |
| Cancer | B1111 | Malignant neoplasm of pyloric canal of stomach |
| Cancer | B4Ay. | Malignant neoplasm of other urinary organs |
| Cancer | ByuD0 | [X]Other Hodgkin's disease |
| Cancer | B14.. | Malignant neoplasm of rectum, rectosigmoid junction and anus |
| Cancer | B492. | Malignant neoplasm of lateral wall of urinary bladder |
| Cancer | B4703 | Teratoma of undescended testis |
| Cancer | B6220 | Sezary's disease of unspecified site |
| Cancer | B350. | Malignant neoplasm of nipple and areola of male breast |
| Cancer | B010z | Malignant neoplasm of fixed part of tongue NOS |
| Cancer | B305. | Malignant neoplasm of hand bones |
| Cancer | B3153 | Malig neopl of connective and soft tissue - sacrum or coccyx |
| Cancer | B48y1 | Malignant neoplasm of tunica vaginalis |
| Cancer | B6024 | Burkitt's lymphoma of lymph nodes of axilla and upper limb |
| Cancer | B00zz | Malignant neoplasm of lip, vermilion border NOS |
| Cancer | B3312 | Malignant neoplasm of lower eyelid |
| Cancer | B317. | Malignant neoplasm, overlap lesion connective & soft tissue |
| Cancer | B62z6 | Unspec malig neop lymphoid/histiocytic of intrapelvic nodes |
| Cancer | Byu58 | [X]Mal neoplasm/connective+soft tissue of trunk,unspecified |
| Cancer | B6008 | Reticulosarcoma of lymph nodes of multiple sites |
| Cancer | B3114 | Malignant neoplasm of connective and soft tissue of finger |
| Cancer | B005. | Malignant neoplasm of commissure of lip |
| Cancer | B171. | Malignant neoplasm of body of pancreas |
| Cancer | B3354 | Malignant neoplasm of skin of umbilicus |
| Cancer | B000z | Malignant neoplasm of upper lip, vermilion border NOS |
| Cancer | ByuD3 | [X]Other specified types of non-Hodgkin's lymphoma |
| Cancer | B2231 | Malignant neoplasm of middle lobe of lung |
| Cancer | B3365 | Malignant neoplasm of skin of thumb |
| Cancer | B67yz | Other and unspecified leukaemia NOS |
| Cancer | B6202 | Nodular lymphoma of intrathoracic lymph nodes |
| Cancer | B4100 | Malignant neoplasm of endocervical canal |
| Cancer | B3085 | Malignant neoplasm of lateral cuneiform |
| Cancer | B161. | Malignant neoplasm of extrahepatic bile ducts |
| Cancer | B15z. | Malignant neoplasm of liver and intrahepatic bile ducts NOS |
| Cancer | B315z | Malig neop of connective and soft tissue of pelvis NOS |
| Cancer | B111z | Malignant neoplasm of pylorus of stomach NOS |
| Cancer | B067. | Malignant neoplasm of posterior wall of oropharynx |
| Cancer | B211. | Malignant neoplasm of supraglottis |
| Cancer | B0041 | Malignant neoplasm of lip unspecified, frenulum |
| Cancer | B41yz | Malignant neoplasm of other site of cervix NOS |
| Cancer | B308z | Malignant neoplasm of short bones of leg NOS |
| Cancer | B49y0 | Malignant neoplasm, overlapping lesion of bladder |
| Cancer | B6247 | Leukaemic reticuloendotheliosis of spleen |
| Cancer | B6120 | Hodgkin's sarcoma of unspecified site |
| Cancer | B52z. | Malignant neoplasm of nervous system NOS |
| Cancer | B65y0 | Aleukaemic myeloid leukaemia |
| Cancer | B332z | Malig neop skin of ear and external auricular canal NOS |
| Cancer | B30X. | Malignant neoplasm/bones+articular cartilage/limb,unspfd |
| Cancer | B302. | Malignant neoplasm of vertebral column |
| Cancer | B5100 | Malignant neoplasm of basal ganglia |
| Cancer | B62x2 | Peripheral T-cell lymphoma |
| Cancer | B6018 | Lymphosarcoma of lymph nodes of multiple sites |
| Cancer | B484. | Malignant neoplasm of epididymis |
| Cancer | B6256 | Letterer-Siwe disease of intrapelvic lymph nodes |
| Cancer | B5451 | Malignant neoplasm of aortic body |
| Cancer | B50z. | Malignant neoplasm of eye NOS |
| Cancer | B6152 | Hodgkin's mixed cellularity of intrathoracic lymph nodes |
| Cancer | B31y. | Malig neop connective and soft tissue other specified site |
| Cancer | B3362 | Malignant neoplasm of skin of fore-arm |
| Cancer | B3320 | Malignant neoplasm of skin of auricle (ear) |
| Cancer | B62y5 | Malignant lymphoma NOS of lymph node inguinal region and leg |
| Cancer | B555. | Malignant neoplasm of lower limb NOS |
| Cancer | B6157 | Hodgkin's disease, mixed cellularity of spleen |
| Cancer | B430. | Malignant neoplasm of corpus uteri, excluding isthmus |
| Cancer | B625. | Letterer-Siwe disease |
| Cancer | B3055 | Malignant neoplasm of carpal bone - trapezoid |
| Cancer | B071. | Malignant neoplasm of posterior wall of nasopharynx |
| Cancer | B62x4 | Malignant reticulosis |
| Cancer | B6155 | Hodgkin's mixed cellularity of lymph nodes inguinal and leg |
| Cancer | B3230 | Malignant melanoma of external surface of cheek |
| Cancer | B6151 | Hodgkin's mixed cellularity of lymph nodes head, face, neck |
| Cancer | B621z | Mycosis fungoides NOS |
| Cancer | B163. | Malignant neoplasm, overlapping lesion of biliary tract |
| Cancer | B1801 | Malignant neoplasm of perinephric tissue |
| Cancer | B616. | Hodgkin's disease, lymphocytic depletion |
| Cancer | B214. | Malignant neoplasm, overlapping lesion of larynx |
| Cancer | B627W | Unspecified B-cell non-Hodgkin's lymphoma |
| Cancer | B6252 | Letterer-Siwe disease of intrathoracic lymph nodes |
| Cancer | B6262 | Mast cell malignancy of intrathoracic lymph nodes |
| Cancer | ByuD5 | [X]Other lymphoid leukaemia |
| Cancer | B62z0 | Unspec malig neop lymphoid/histiocytic of unspecified site |
| Cancer | Byu54 | [X]Malignant neoplasm/peripheral nerves of trunk,unspecified |
| Cancer | B18y5 | Malignant neoplasm of pelvic peritoneum |
| Cancer | B25.. | Malig neo, overlapping lesion of heart, mediastinum & pleura |
| Cancer | B30W. | Malignant neoplasm/overlap lesion/bone+articulr cartilage |
| Cancer | B62x5 | Malignant immunoproliferative small intestinal disease |
| Cancer | B3253 | Malignant melanoma of groin |
| Cancer | B670. | Acute erythraemia and erythroleukaemia |
| Cancer | B64y1 | Prolymphocytic leukaemia |
| Cancer | B061. | Malignant neoplasm of tonsillar fossa |
| Cancer | B335z | Malignant neoplasm of skin of trunk, excluding scrotum, NOS |
| Cancer | B13.. | Malignant neoplasm of colon |
| Cancer | B545z | Malignant neoplasm of aortic body or paraganglia NOS |
| Cancer | B310z | Malig neop connective and soft tissue head, face, neck NOS |
| Cancer | B05z. | Malignant neoplasm of mouth NOS |
| Cancer | B3332 | Malignant neoplasm of skin of eyebrow |
| Cancer | B642. | Subacute lymphoid leukaemia |
| Cancer | B5151 | Malignant neoplasm of floor of cerebral ventricle |
| Cancer | B62x1 | Lymphoepithelioid lymphoma |
| Cancer | B121. | Malignant neoplasm of jejunum |
| Cancer | B6027 | Burkitt's lymphoma of spleen |
| Cancer | B3008 | Malignant neoplasm of temporal bone |
| Cancer | B342. | Malignant neoplasm of upper-inner quadrant of female breast |
| Cancer | B324z | Malignant melanoma of scalp and neck NOS |
| Cancer | B18y1 | Malignant neoplasm of mesocaecum |
| Cancer | B6110 | Hodgkin's granuloma of unspecified site |
| Cancer | B322z | Malignant melanoma of ear and external auricular canal NOS |
| Cancer | B013. | Malignant neoplasm of ventral surface of tongue |
| Cancer | B485. | Malignant neoplasm of spermatic cord |
| Cancer | B103. | Malignant neoplasm of upper third of oesophagus |
| Cancer | B3333 | Malignant neoplasm of skin of forehead |
| Cancer | ZV103 | [V]Personal history of malignant neoplasm of breast |
| Cancer | B222z | Malignant neoplasm of upper lobe, bronchus or lung NOS |
| Cancer | B653z | Myeloid sarcoma NOS |
| Cancer | B0z2. | Malignant neoplasm of laryngopharynx |
| Cancer | B3... | Malig neop of bone, connective tissue, skin and breast |
| Cancer | Byu7. | [X]Malignant neoplasm of female genital organs |
| Cancer | B615. | Hodgkin's disease, mixed cellularity |
| Cancer | B61zz | Hodgkin's disease NOS |
| Cancer | B3277 | Malignant melanoma of foot |
| Cancer | B2130 | Malignant neoplasm of arytenoid cartilage |
| Cancer | B61z. | Hodgkin's disease NOS |
| Cancer | B4700 | Malignant neoplasm of ectopic testis |
| Cancer | B631. | Plasma cell leukaemia |
| Cancer | B65.. | Myeloid leukaemia |
| Cancer | B312. | Malig neop of connective and soft tissue of hip and leg |
| Cancer | B3401 | Malignant neoplasm of areola of female breast |
| Cancer | B4A11 | Malignant neoplasm of ureteropelvic junction |
| Cancer | B600z | Reticulosarcoma NOS |
| Cancer | B11y0 | Malignant neoplasm of anterior wall of stomach NEC |
| Cancer | B620. | Nodular lymphoma (Brill - Symmers disease) |
| Cancer | B323. | Malignant melanoma of other and unspecified parts of face |
| Cancer | B05.. | Malignant neoplasm of other and unspecified parts of mouth |
| Cancer | B487. | Malignant neoplasm, overlapping lesion of penis |
| Cancer | B554. | Malignant neoplasm of upper limb NOS |
| Cancer | B336. | Malignant neoplasm of skin of upper limb and shoulder |
| Cancer | B350z | Malignant neoplasm of nipple or areola of male breast NOS |
| Cancer | B3378 | Malignant neoplasm of skin of toe |
| Cancer | B138. | Malignant neoplasm, overlapping lesion of colon |
| Cancer | B49.. | Malignant neoplasm of urinary bladder |
| Cancer | B440. | Malignant neoplasm of ovary |
| Cancer | ZV105 | [V]Personal history of malignant neoplasm of urinary organ |
| Cancer | B301. | Malignant neoplasm of mandible |
| Cancer | B6156 | Hodgkin's mixed cellularity of intrapelvic lymph nodes |
| Cancer | B010. | Malignant neoplasm of base of tongue |
| Cancer | B064z | Malignant neoplasm of anterior epiglottis NOS |
| Cancer | B6225 | Sezary's disease of lymph nodes of inguinal region and leg |
| Cancer | B1501 | Hepatoblastoma of liver |
| Cancer | Byu41 | [X]Malignant melanoma of skin, unspecified |
| Cancer | B48y0 | Malignant neoplasm of seminal vesicle |
| Cancer | B65yz | Other myeloid leukaemia NOS |
| Cancer | B6000 | Reticulosarcoma of unspecified site |
| Cancer | B496. | Malignant neoplasm of ureteric orifice |
| Cancer | B51y2 | Malignant neoplasm, overlapping lesion of brain |
| Cancer | B6224 | Sezary's disease of lymph nodes of axilla and upper limb |
| Cancer | B011. | Malignant neoplasm of dorsal surface of tongue |
| Cancer | B5171 | Malignant neoplasm of medulla oblongata |
| Cancer | B6128 | Hodgkin's sarcoma of lymph nodes of multiple sites |
| Cancer | B056. | Malignant neoplasm of retromolar area |
| Cancer | B305A | Malignant neoplasm of third metacarpal bone |
| Cancer | B060z | Malignant neoplasm tonsil NOS |
| Cancer | B442. | Malignant neoplasm of broad ligament |
| Cancer | B614. | Hodgkin's disease, nodular sclerosis |
| Cancer | B2241 | Malignant neoplasm of lower lobe of lung |
| Cancer | B6242 | Leukaemic reticuloendotheliosis of intrathoracic lymph nodes |
| Cancer | B6125 | Hodgkin's sarcoma of lymph nodes of inguinal region and leg |
| Cancer | B322. | Malignant melanoma of ear and external auricular canal |
| Cancer | B6241 | Leukaemic reticuloend of lymph nodes of head, face and neck |
| Cancer | B3110 | Malignant neoplasm of connective and soft tissue of shoulder |
| Cancer | B35z. | Malignant neoplasm of other site of male breast |
| Cancer | ZV106 | [V]Personal history of leukaemia |
| Cancer | B05z0 | Kaposi's sarcoma of palate |
| Cancer | B6238 | Malignant histiocytosis of lymph nodes of multiple sites |
| Cancer | B6145 | Hodgkin's nodular sclerosis of inguinal region and leg |
| Cancer | B66yz | Other monocytic leukaemia NOS |
| Cancer | B1611 | Malignant neoplasm of hepatic duct |
| Cancer | B450z | Malignant neoplasm of vagina NOS |
| Cancer | B6270 | Follicular non-Hodgkin's small cleaved cell lymphoma |
| Cancer | B524X | Malignant neoplasm/peripheral nerves of trunk,unspecified |
| Cancer | B35z0 | Malignant neoplasm of ectopic site of male breast |
| Cancer | B04z. | Malignant neoplasm of floor of mouth NOS |
| Cancer | B3321 | Malignant neoplasm of skin of external auditory meatus |
| Cancer | B01.. | Malignant neoplasm of tongue |
| Cancer | B5003 | Malignant neoplasm of sclera |
| Cancer | B02.. | Malignant neoplasm of major salivary glands |
| Cancer | B67y. | Other and unspecified leukaemia |
| Cancer | B5241 | Malignant neoplasm of peripheral nerve,upp limb,incl should |
| Cancer | B18z. | Malignant neoplasm of retroperitoneum and peritoneum NOS |
| Cancer | B5420 | Malignant neoplasm of pituitary gland |
| Cancer | Byu5. | [X]Malignant neoplasm of mesothelial and soft tissue |
| Cancer | B6204 | Nodular lymphoma of lymph nodes of axilla and upper limb |
| Cancer | B545. | Malignant neoplasm of aortic body and other paraganglia |
| Cancer | Byu.. | [X]Additional neoplasm classification terms |
| Cancer | B300z | Malignant neoplasm of bones of skull and face NOS |
| Cancer | B662. | Subacute monocytic leukaemia |
| Cancer | B6114 | Hodgkin's granuloma of lymph nodes of axilla and upper limb |
| Cancer | B6161 | Hodgkin's lymphocytic depletion of head, face and neck |
| Cancer | B6021 | Burkitt's lymphoma of lymph nodes of head, face and neck |
| Cancer | B1z11 | Fibrosarcoma of spleen |
| Cancer | B3232 | Malignant melanoma of eyebrow |
| Cancer | B62y8 | Malignant lymphoma NOS of lymph nodes of multiple sites |
| Cancer | B172. | Malignant neoplasm of tail of pancreas |
| Cancer | B62z1 | Unspec malig neop lymphoid/histiocytic lymph node head/neck |
| Cancer | B3252 | Malignant melanoma of buttock |
| Cancer | B6163 | Hodgkin's lymphocytic depletion intra-abdominal lymph nodes |
| Cancer | B08y. | Malignant neoplasm of other specified hypopharyngeal site |
| Cancer | B6207 | Nodular lymphoma of spleen |
| Cancer | B6250 | Letterer-Siwe disease of unspecified sites |
| Cancer | B3264 | Malignant melanoma of finger |
| Cancer | B6y.. | Malignant neoplasm lymphatic or haematopoietic tissue OS |
| Cancer | ByuA1 | [X]Malignant neoplasm/central nervous system, unspecified |
| Cancer | B074. | Malignant neoplasm, overlapping lesion of nasopharynx |
| Cancer | B0030 | Malignant neoplasm of lower lip, buccal aspect |
| Cancer | B182. | Overlapping malign lesion of retroperitoneum and peritoneum |
| Cancer | B300. | Malignant neoplasm of bones of skull and face |
| Cancer | Byu51 | [X]Mesothelioma, unspecified |
| Cancer | Byu40 | [X]Malignant melanoma of other+unspecified parts of face |
| Cancer | B6251 | Letterer-Siwe disease of lymph nodes of head, face and neck |
| Cancer | B4501 | Malignant neoplasm of vaginal vault |
| Cancer | B6206 | Nodular lymphoma of intrapelvic lymph nodes |
| Cancer | B18y. | Malignant neoplasm of specified parts of peritoneum |
| Cancer | B1802 | Malignant neoplasm of retrocaecal tissue |
| Cancer | B0042 | Malignant neoplasm of lip unspecified, mucosa |
| Cancer | B2zy. | Malignant neoplasm of other site of respiratory tract |
| Cancer | B612z | Hodgkin's sarcoma NOS |
| Cancer | B175. | Malignant neoplasm, overlapping lesion of pancreas |
| Cancer | B0021 | Malignant neoplasm of upper lip, frenulum |
| Cancer | B64.. | Lymphoid leukaemia |
| Cancer | ByuD4 | [X]Other malignant immunoproliferative diseases |
| Cancer | B3y.. | Malig neop of bone, connective tissue, skin and breast OS |
| Cancer | B54.. | Malig neop of other endocrine glands and related structures |
| Cancer | B10.. | Malignant neoplasm of oesophagus |
| Cancer | B6138 | Hodgkin's, lymphocytic-histiocytic pred of multiple sites |
| Cancer | B524. | Malig neopl peripheral nerves and autonomic nervous system |
| Cancer | B5505 | Malignant neoplasm of supraclavicular fossa NOS |
| Cancer | B610. | Hodgkin's paragranuloma |
| Cancer | B325. | Malignant melanoma of trunk (excluding scrotum) |
| Cancer | B6118 | Hodgkin's granuloma of lymph nodes of multiple sites |
| Cancer | B3377 | Malignant neoplasm of skin of foot |
| Cancer | B6107 | Hodgkin's paragranuloma of spleen |
| Cancer | B070. | Malignant neoplasm of roof of nasopharynx |
| Cancer | B4z.. | Malignant neoplasm of genitourinary organ NOS |
| Cancer | B3262 | Malignant melanoma of fore-arm |
| Cancer | B14y. | Malig neop other site rectum, rectosigmoid junction and anus |
| Cancer | B60z. | Reticulosarcoma or lymphosarcoma NOS |
| Cancer | Byu3. | [X]Malignant neoplasm of bone and articular cartilage |
| Cancer | B65z. | Myeloid leukaemia NOS |
| Cancer | B5102 | Malignant neoplasm of corpus striatum |
| Cancer | B4303 | Malignant neoplasm of myometrium of corpus uteri |
| Cancer | B2z.. | Malig neop other/ill-defined sites resp/intrathoracic organs |
| Cancer | B3255 | Malignant melanoma of perineum |
| Cancer | B5150 | Malignant neoplasm of choroid plexus |
| Cancer | B22z. | Malignant neoplasm of bronchus or lung NOS |
| Cancer | B4701 | Malignant neoplasm of retained testis |
| Cancer | B3241 | Malignant melanoma of neck |
| Cancer | B611. | Hodgkin's granuloma |
| Cancer | B3034 | Malignant neoplasm of costo-vertebral joint |
| Cancer | B6266 | Mast cell malignancy of intrapelvic lymph nodes |
| Cancer | B1514 | Malignant neoplasm of intrahepatic gall duct |
| Cancer | B1513 | Malignant neoplasm of intrahepatic canaliculi |
| Cancer | B55y1 | Malignant neoplasm of trunk NOS |
| Cancer | B33X. | Malignant neoplasm overlapping lesion of skin |
| Cancer | B3400 | Malignant neoplasm of nipple of female breast |
| Cancer | B5001 | Malignant neoplasm of iris |
| Cancer | B6... | Malignant neoplasm of lymphatic and haemopoietic tissue |
| Cancer | B102. | Malignant neoplasm of abdominal oesophagus |
| Cancer | B6231 | Malignant histiocytosis of lymph nodes head, face and neck |
| Cancer | B326z | Malignant melanoma of upper limb or shoulder NOS |
| Cancer | B21y. | Malignant neoplasm of larynx, other specified site |
| Cancer | B61z8 | Hodgkin's disease NOS of lymph nodes of multiple sites |
| Cancer | B2z0. | Malig neop of upper respiratory tract, part unspecified |
| Cancer | B006. | Malignant neoplasm of overlapping lesion of lip |
| Cancer | B023. | Malignant neoplasm, overlapping lesion of major saliv gland |
| Cancer | Byu6. | [X]Malignant neoplasm of breast |
| Cancer | B613. | Hodgkin's disease, lymphocytic-histiocytic predominance |
| Cancer | B309. | Malignant neoplasm, overlap les bone and artic cart of limbs |
| Cancer | B6160 | Hodgkin's lymphocytic depletion of unspecified site |
| Cancer | B303z | Malignant neoplasm of rib, sternum and clavicle NOS |
| Cancer | B486. | Malignant neoplasm of scrotum |
| Cancer | B308. | Malignant neoplasm of short bones of leg |
| Cancer | B3089 | Malignant neoplasm of second metatarsal bone |
| Cancer | B3131 | Malignant neoplasm of diaphragm |
| Cancer | B5401 | Malignant neoplasm of adrenal medulla |
| Cancer | B3064 | Malignant neoplasm of coccygeal vertebra |
| Cancer | B671. | Chronic erythraemia |
| Cancer | B123. | Malignant neoplasm of Meckel's diverticulum |
| Cancer | B002. | Malignant neoplasm of upper lip, inner aspect |
| Cancer | B343. | Malignant neoplasm of lower-inner quadrant of female breast |
| Cancer | B6146 | Hodgkin's nodular sclerosis of intrapelvic lymph nodes |
| Cancer | B4A1z | Malignant neoplasm of renal pelvis NOS |
| Cancer | B113. | Malignant neoplasm of fundus of stomach |
| Cancer | B3103 | Malignant neoplasm of cartilage of ear |
| Cancer | B68z. | Leukaemia NOS |
| Cancer | B6260 | Mast cell malignancy of unspecified site |
| Cancer | B3272 | Malignant melanoma of knee |
| Cancer | B4... | Malignant neoplasm of genitourinary organ |
| Cancer | B31.. | Malignant neoplasm of connective and other soft tissue |
| Cancer | B00z0 | Malignant neoplasm of lip, unspecified, external |
| Cancer | B34.. | Malignant neoplasm of female breast |
| Cancer | B62y7 | Malignant lymphoma NOS of spleen |
| Cancer | B3372 | Malignant neoplasm of skin of knee |
| Cancer | B016. | Malignant neoplasm of lingual tonsil |
| Cancer | B517z | Malignant neoplasm of brain stem NOS |
| Cancer | B16z. | Malignant neoplasm gallbladder/extrahepatic bile ducts NOS |
| Cancer | B4A.. | Malig neop of kidney and other unspecified urinary organs |
| Cancer | B313. | Malignant neoplasm of connective and soft tissue of thorax |
| Cancer | B013z | Malignant neoplasm of ventral tongue surface NOS |
| Cancer | B6245 | Leukaemic reticuloend of lymph nodes inguinal region and leg |
| Cancer | B3040 | Malignant neoplasm of scapula |
| Cancer | B5242 | Malignant neoplasm of peripheral nerve of low limb, incl hip |
| Cancer | B508. | Malignant neoplasm, overlapping lesion of eye and adnexa |
| Cancer | B62z8 | Unspec malig neop lymphoid/histiocytic of multiple sites |
| Cancer | B6253 | Letterer-Siwe disease of intra-abdominal lymph nodes |
| Cancer | B07y. | Malignant neoplasm of other specified site of nasopharynx |
| Cancer | B494. | Malignant neoplasm of posterior wall of urinary bladder |
| Cancer | B0043 | Malignant neoplasm of lip, oral aspect |
| Cancer | B66z. | Monocytic leukaemia NOS |
| Cancer | B6001 | Reticulosarcoma of lymph nodes of head, face and neck |
| Cancer | B1502 | Primary angiosarcoma of liver |
| Cancer | B6510 | Chronic eosinophilic leukaemia |
| Cancer | B17yz | Malignant neoplasm of specified site of pancreas NOS |
| Cancer | Byu82 | [X]Malignant neoplasm of male genital organ, unspecified |
| Cancer | B1510 | Malignant neoplasm of interlobular bile ducts |
| Cancer | B62y3 | Malignant lymphoma NOS of intra-abdominal lymph nodes |
| Cancer | B30.. | Malignant neoplasm of bone and articular cartilage |
| Cancer | B05y. | Malignant neoplasm of other specified mouth parts |
| Cancer | B3361 | Malignant neoplasm of skin of upper arm |
| Cancer | B150z | Primary malignant neoplasm of liver NOS |
| Cancer | B3340 | Malignant neoplasm of scalp |
| Cancer | B61z6 | Hodgkin's disease NOS of intrapelvic lymph nodes |
| Cancer | B524W | Mal neoplasm/periph nerves+autonomic nervous system,unspc |
| Cancer | B0z1. | Malignant neoplasm of Waldeyer's ring |
| Cancer | B2221 | Malignant neoplasm of upper lobe of lung |
| Cancer | B174. | Malignant neoplasm of Islets of Langerhans |
| Cancer | B17.. | Malignant neoplasm of pancreas |
| Cancer | B18y7 | Malignant neoplasm of mesentery |
| Cancer | B5101 | Malignant neoplasm of cerebral cortex |
| Cancer | B1zz. | Malignant neoplasm of digestive tract and peritoneum NOS |
| Cancer | B6117 | Hodgkin's granuloma of spleen |
| Cancer | B506. | Malignant neoplasm of choroid |
| Cancer | B2210 | Malignant neoplasm of carina of bronchus |
| Cancer | B22.. | Malignant neoplasm of trachea, bronchus and lung |
| Cancer | B627. | Non - Hodgkin's lymphoma |
| Cancer | B5... | Malignant neoplasm of other and unspecified sites |
| Cancer | B5502 | Malignant neoplasm of nose NOS |
| Cancer | B5531 | Malignant neoplasm of presacral region |
| Cancer | B3111 | Malignant neoplasm of connective and soft tissue, upper arm |
| Cancer | B6257 | Letterer-Siwe disease of spleen |
| Cancer | B516. | Malignant neoplasm of cerebellum |
| Cancer | B302z | Malignant neoplasm of vertebral column NOS |
| Cancer | B62x3 | Malignant reticuloendotheliosis |
| Cancer | B3z.. | Malig neop of bone, connective tissue, skin and breast NOS |
| Cancer | B6228 | Sezary's disease of lymph nodes of multiple sites |
| Cancer | B3360 | Malignant neoplasm of skin of shoulder |
| Cancer | B61z5 | Hodgkin's disease NOS of lymph nodes inguinal region and leg |
| Cancer | B161z | Malignant neoplasm of extrahepatic bile ducts NOS |
| Cancer | B53.. | Malignant neoplasm of thyroid gland |
| Cancer | B62x. | Malignant lymphoma otherwise specified |
| Cancer | B122. | Malignant neoplasm of ileum |
| Cancer | B26.. | Malignant neoplasm, overlap lesion of resp & intrathor orgs |
| Cancer | B4A3. | Malignant neoplasm of urethra |
| Cancer | B62z4 | Unspec malig neop lymphoid/histiocytic lymph node axilla/arm |
| Cancer | ByuD2 | [X]Other types of diffuse non-Hodgkin's lymphoma |
| Cancer | B523. | Malignant neoplasm of spinal meninges |
| Cancer | B152. | Malignant neoplasm of liver unspecified |
| Cancer | B18yz | Malignant neoplasm of specified parts of peritoneum NOS |
| Cancer | B3276 | Malignant melanoma of heel |
| Cancer | B306. | Malignant neoplasm of pelvic bones, sacrum and coccyx |
| Cancer | B49y. | Malignant neoplasm of other site of urinary bladder |
| Cancer | B00y. | Malignant neoplasm of other sites of lip |
| Cancer | B55y0 | Malignant neoplasm of back NOS |
| Cancer | B3352 | Malignant neoplasm of skin of breast |
| Cancer | B13z. | Malignant neoplasm of colon NOS |
| Cancer | B3123 | Malig neop of connective and soft tissue of lower leg |
| Cancer | B071z | Malignant neoplasm of posterior wall of nasopharynx NOS |
| Cancer | B072z | Malignant neoplasm of lateral wall of nasopharynx NOS |
| Cancer | B0640 | Malignant neoplasm of epiglottis, free border |
| Cancer | B652. | Subacute myeloid leukaemia |
| Cancer | B03.. | Malignant neoplasm of gum |
| Cancer | B6166 | Hodgkin's lymphocytic depletion of intrapelvic lymph nodes |
| Cancer | Byu31 | [X]Malignant neoplasm/bones+articular cartilage/limb,unspfd |
| Cancer | B203. | Malignant neoplasm of ethmoid sinus |
| Cancer | B30z. | Malignant neoplasm of bone and articular cartilage NOS |
| Cancer | B215. | Malignant neoplasm of epiglottis NOS |
| Cancer | B6212 | Mycosis fungoides of intrathoracic lymph nodes |
| Cancer | B110z | Malignant neoplasm of cardia of stomach NOS |
| Cancer | B003z | Malignant neoplasm of lower lip, inner aspect NOS |
| Cancer | B6221 | Sezary's disease of lymph nodes of head, face and neck |
| Cancer | B5000 | Malignant neoplasm of ciliary body |
| Cancer | B6217 | Mycosis fungoides of spleen |
| Cancer | B520. | Malignant neoplasm of cranial nerves |
| Cancer | B3261 | Malignant melanoma of upper arm |
| Cancer | B3374 | Malignant neoplasm of skin of lower leg |
| Cancer | B540. | Malignant neoplasm of adrenal gland |
| Cancer | B62y0 | Malignant lymphoma NOS of unspecified site |
| Cancer | B311. | Malig neop connective and soft tissue upper limb/shoulder |
| Cancer | B084. | Malignant neoplasm, overlapping lesion of hypopharynx |
| Cancer | Byu13 | [X]Malignant neoplsm/ill-defin sites within digestive system |
| Cancer | B513. | Malignant neoplasm of parietal lobe |
| Cancer | B5246 | Malignant neoplasm,overlap lesion periph nerve & auton ns |
| Cancer | B12y. | Malignant neoplasm of other specified site small intestine |
| Cancer | B6265 | Mast cell malignancy of lymph nodes inguinal region and leg |
| Cancer | B6275 | Diffuse non-Hodgkin mixed sml & lge cell (diffuse) lymphoma |
| Cancer | B2012 | Malignant neoplasm of tympanic antrum |
| Cancer | B6015 | Lymphosarcoma of lymph nodes of inguinal region and leg |
| Cancer | B43z. | Malignant neoplasm of body of uterus NOS |
| Cancer | B51yz | Malignant neoplasm of other part of brain NOS |
| Cancer | B6014 | Lymphosarcoma of lymph nodes of axilla and upper limb |
| Cancer | B450. | Malignant neoplasm of vagina |
| Cancer | B055. | Malignant neoplasm of palate unspecified |
| Cancer | B063. | Malignant neoplasm of vallecula |
| Cancer | B653. | Myeloid sarcoma |
| Cancer | Byu20 | [X]Malignant neoplasm of bronchus or lung, unspecified |
| Cancer | B33z. | Malignant neoplasm of skin NOS |
| Cancer | B4300 | Malignant neoplasm of cornu of corpus uteri |
| Cancer | B6132 | Hodgkin's, lymphocytic-histiocytic pred intrathoracic nodes |
| Cancer | B5452 | Malignant neoplasm of coccygeal body |
| Cancer | B6016 | Lymphosarcoma of intrapelvic lymph nodes |
| Cancer | B601z | Lymphosarcoma NOS |
| Cancer | B410. | Malignant neoplasm of endocervix |
| Cancer | B3233 | Malignant melanoma of forehead |
| Cancer | B00z. | Malignant neoplasm of vermilion border of lip unspecified |
| Cancer | B02z. | Malignant neoplasm of major salivary gland NOS |
| Cancer | B3001 | Malignant neoplasm of frontal bone |
| Cancer | B204. | Malignant neoplasm of frontal sinus |
| Cancer | B6216 | Mycosis fungoides of intrapelvic lymph nodes |
| Cancer | B6122 | Hodgkin's sarcoma of intrathoracic lymph nodes |
| Cancer | ByuE. | [X]Malignant neoplasms/independent (primary) multiple sites |
| Cancer | B62z. | Malignant neoplasms of lymphoid and histiocytic tissue NOS |
| Cancer | B602. | Burkitt's lymphoma |
| Cancer | B5200 | Malignant neoplasm of olfactory bulb |
| Cancer | B67.. | Other specified leukaemia |
| Cancer | B3273 | Malignant melanoma of popliteal fossa area |
| Cancer | B52.. | Malig neop of other and unspecified parts of nervous system |
| Cancer | B10z. | Malignant neoplasm of oesophagus NOS |
| Cancer | B5002 | Malignant neoplasm of crystalline lens |
| Cancer | B62z5 | Unspec malig neop lymphoid/histiocytic nodes inguinal/leg |
| Cancer | B10y. | Malignant neoplasm of other specified part of oesophagus |
| Cancer | ZV100 | [V]Personal history of malig neop of gastrointestinal tract |
| Cancer | B62xX | Oth and unspecif peripheral & cutaneous T-cell lymphomas |
| Cancer | B6154 | Hodgkin's mixed cellularity of lymph nodes of axilla and arm |
| Cancer | B6211 | Mycosis fungoides of the lymph nodes of head, face and neck |
| Cancer | B64y. | Other lymphoid leukaemia |
| Cancer | B550z | Malignant neoplasm of head, neck and face NOS |
| Cancer | B55z. | Malignant neoplasm of other and ill defined site NOS |
| Cancer | B44y. | Malignant neoplasm of other site of uterine adnexa |
| Cancer | B065. | Malignant neoplasm of junctional region of epiglottis |
| Cancer | ByuA0 | [X]Malignant neoplasm/other and unspecified cranial nerves |
| Cancer | B6147 | Hodgkin's disease, nodular sclerosis of spleen |
| Cancer | B54X. | Malignant neoplasm-pluriglandular involvement,unspecified |
| Cancer | B515. | Malignant neoplasm of cerebral ventricles |
| Cancer | B6254 | Letterer-Siwe disease of lymph nodes of axilla and arm |
| Cancer | ByuA2 | [X]Malignant neoplasm of meninges, unspecified |
| Cancer | Byu25 | [X]Malignant neoplasm of mediastinum, part unspecified |
| Cancer | B613z | Hodgkin's, lymphocytic-histiocytic predominance NOS |
| Cancer | B330. | Malignant neoplasm of skin of lip |
| Cancer | B515z | Malignant neoplasm of cerebral ventricle NOS |
| Cancer | B2002 | Malignant neoplasm of septum of nose |
| Cancer | B1612 | Malignant neoplasm of common bile duct |
| Cancer | Byu52 | [X]Kaposi's sarcoma of multiple organs |
| Cancer | B0130 | Malignant neoplasm of anterior 2/3 of tongue ventral surface |
| Cancer | B01z. | Malignant neoplasm of tongue NOS |
| Cancer | B2200 | Malignant neoplasm of cartilage of trachea |
| Cancer | B312z | Malig neop connective and soft tissue hip and leg NOS |
| Cancer | B3357 | Malignant neoplasm of skin of back |
| Cancer | B45y0 | Malignant neoplasm of overlapping lesion of vulva |
| Cancer | B031. | Malignant neoplasm of lower gum |
| Cancer | B625z | Letterer-Siwe disease NOS |
| Cancer | B54z. | Malig neop of endocrine gland or related structure NOS |
| Cancer | B4y.. | Malignant neoplasm of genitourinary organ OS |
| Cancer | B320. | Malignant melanoma of lip |
| Cancer | B08z. | Malignant neoplasm of hypopharynx NOS |
| Cancer | B213z | Malignant neoplasm of laryngeal cartilage NOS |
| Cancer | B303. | Malignant neoplasm of ribs, sternum and clavicle |
| Cancer | B324. | Malignant melanoma of scalp and neck |
| Cancer | B690. | Acute myelomonocytic leukaemia |
| Cancer | B240. | Malignant neoplasm of thymus |
| Cancer | B54y. | Malignant neoplasm of other specified endocrine gland |
| Cancer | B181. | Mesothelioma of peritoneum |
| Cancer | B224. | Malignant neoplasm of lower lobe, bronchus or lung |
| Cancer | B6264 | Mast cell malignancy of lymph nodes of axilla and upper limb |
| Cancer | B3274 | Malignant melanoma of lower leg |
| Cancer | B47z. | Malignant neoplasm of testis NOS |
| Cancer | B6136 | Hodgkin's, lymphocytic-histiocytic pred intrapelvic nodes |
| Cancer | B6y0. | Myeloproliferative disorder |
| Cancer | B223. | Malignant neoplasm of middle lobe, bronchus or lung |
| Cancer | B326. | Malignant melanoma of upper limb and shoulder |
| Cancer | B630. | Multiple myeloma |
| Cancer | B523z | Malignant neoplasm of spinal meninges NOS |
| Cancer | B525. | Malignant neoplasm of cauda equina |
| Cancer | B51y0 | Malignant neoplasm of corpus callosum |
| Cancer | B65y. | Other myeloid leukaemia |
| Cancer | B3088 | Malignant neoplasm of first metatarsal bone |
| Cancer | B001. | Malignant neoplasm of lower lip, vermilion border |
| Cancer | Byu2. | [X]Malignant neoplasm of respiratory and intrathoracic orga |
| Cancer | B6150 | Hodgkin's disease, mixed cellularity of unspecified site |
| Cancer | B4A0. | Malignant neoplasm of kidney parenchyma |
| Cancer | B6278 | Diffuse non-Hodgkin's lymphoma undifferentiated (diffuse) |
| Cancer | B62x0 | T-zone lymphoma |
| Cancer | B3258 | Malignant melanoma of chest wall |
| Cancer | B130. | Malignant neoplasm of hepatic flexure of colon |
| Cancer | B60y. | Other specified reticulosarcoma or lymphosarcoma |
| Cancer | B3322 | Malignant neoplasm of pinna NEC |
| Cancer | B412. | Malignant neoplasm, overlapping lesion of cervix uteri |
| Cancer | B5510 | Malignant neoplasm of axilla NOS |
| Cancer | B520z | Malignant neoplasm of cranial nerves NOS |
| Cancer | B112. | Malignant neoplasm of pyloric antrum of stomach |
| Cancer | ZV10z | [V]Personal history of unspecified malignant neoplasm |
| Cancer | B497. | Malignant neoplasm of urachus |
| Cancer | B6106 | Hodgkin's paragranuloma of intrapelvic lymph nodes |
| Cancer | B213. | Malignant neoplasm of laryngeal cartilage |
| Cancer | B675. | Acute myelofibrosis |
| Cancer | B6007 | Reticulosarcoma of spleen |
| Cancer | B24z. | Malignant neoplasm of heart, thymus and mediastinum NOS |
| Cancer | B2zz. | Malignant neoplasm of respiratory tract NOS |
| Cancer | B6012 | Lymphosarcoma of intrathoracic lymph nodes |
| Cancer | B0011 | Malignant neoplasm of lower lip, lipstick area |
| Cancer | B34y. | Malignant neoplasm of other site of female breast |
| Cancer | B6246 | Leukaemic reticuloendotheliosis of intrapelvic lymph nodes |
| Cancer | B4A10 | Malignant neoplasm of renal calyces |
| Cancer | B51y. | Malignant neoplasm of other parts of brain |
| Cancer | B6135 | Hodgkin's, lymphocytic-histiocytic pred inguinal and leg |
| Cancer | B327z | Malignant melanoma of lower limb or hip NOS |
| Cancer | B3124 | Malignant neoplasm of connective and soft tissue of foot |
| Cancer | B61z3 | Hodgkin's disease NOS of intra-abdominal lymph nodes |
| Cancer | B502. | Malignant neoplasm of lacrimal gland |
| Cancer | B0zz. | Malignant neoplasm of lip, oral cavity and pharynx NOS |
| Cancer | B041. | Malignant neoplasm of lateral portion of floor of mouth |
| Cancer | B1... | Malignant neoplasm of digestive organs and peritoneum |
| Cancer | B6276 | Diffuse non-Hodgkin's immunoblastic (diffuse) lymphoma |
| Cancer | Byu56 | [X]Mal neoplasm/periph nerves+autonomic nervous system,unspc |
| Cancer | B6144 | Hodgkin's nodular sclerosis of lymph nodes of axilla and arm |
| Cancer | B337z | Malignant neoplasm of skin of lower limb or hip NOS |
| Cancer | B327. | Malignant melanoma of lower limb and hip |
| Cancer | B6y1. | Myelosclerosis with myeloid metaplasia |
| Cancer | B200z | Malignant neoplasm of nasal cavities NOS |
| Cancer | B661. | Chronic monocytic leukaemia |
| Cancer | B1z1. | Malignant neoplasm of spleen NEC |
| Cancer | B522. | Malignant neoplasm of spinal cord |
| Cancer | B62y1 | Malignant lymphoma NOS of lymph nodes of head, face and neck |
| Cancer | B6013 | Lymphosarcoma of intra-abdominal lymph nodes |
| Cancer | B6234 | Malignant histiocytosis of lymph nodes of axilla and arm |
| Cancer | B2412 | Malignant neoplasm of myocardium |
| Cancer | B12z. | Malignant neoplasm of small intestine NOS |
| Cancer | Byu55 | [X]Mal neoplasm/overlap les/periph nerv+autonomic nerv systm |
| Cancer | B012. | Malignant neoplasm of tongue, tip and lateral border |
| Cancer | ByuD6 | [X]Other myeloid leukaemia |
| Cancer | B11.. | Malignant neoplasm of stomach |
| Cancer | Byu4. | [X]Melanoma and other malignant neoplasms of skin |
| Cancer | B602z | Burkitt's lymphoma NOS |
| Cancer | B314. | Malignant neoplasm of connective and soft tissue of abdomen |
| Cancer | B3356 | Malignant neoplasm of skin of perineum |
| Cancer | B3000 | Malignant neoplasm of ethmoid bone |
| Cancer | B30z0 | Osteosarcoma |
| Cancer | B6168 | Hodgkin's lymphocytic depletion lymph nodes multiple sites |
| Cancer | B2003 | Malignant neoplasm of vestibule of nose |
| Cancer | B004. | Malignant neoplasm of lip unspecified, inner aspect |
| Cancer | B500. | Malig neop eyeball excl conjunctiva, cornea, retina, choroid |
| Cancer | B59zX | Kaposi's sarcoma, unspecified |
| Cancer | B054. | Malignant neoplasm of uvula |
| Cancer | B5173 | Malignant neoplasm of pons |
| Cancer | B3060 | Malignant neoplasm of ilium |
| Cancer | B40.. | Malignant neoplasm of uterus, part unspecified |
| Cancer | B3113 | Malignant neoplasm of connective and soft tissue of hand |
| Cancer | B18y6 | Malignant neoplasm of the pouch of Douglas |
| Cancer | B106. | Malignant neoplasm, overlapping lesion of oesophagus |
| Cancer | B3257 | Malignant melanoma of back |
| Cancer | B18y3 | Malignant neoplasm of omentum |
| Cancer | B073. | Malignant neoplasm of anterior wall of nasopharynx |
| Cancer | B0... | Malignant neoplasm of lip, oral cavity and pharynx |
| Cancer | B133. | Malignant neoplasm of sigmoid colon |
| Cancer | B41y1 | Malignant neoplasm of squamocolumnar junction of cervix |
| Cancer | B00.. | Malignant neoplasm of lip |
| Cancer | ByuDF | [X]Non-Hodgkin's lymphoma, unspecified type |
| Cancer | B627C | Follicular non-Hodgkin's lymphoma |
| Cancer | B6165 | Hodgkin's lymphocytic depletion lymph nodes inguinal and leg |
| Cancer | B4710 | Seminoma of descended testis |
| Cancer | B1zy. | Malignant neoplasm other spec digestive tract and peritoneum |
| Cancer | B47.. | Malignant neoplasm of testis |
| Cancer | B3035 | Malignant neoplasm of xiphoid process |
| Cancer | B0550 | Malignant neoplasm of junction of hard and soft palate |
| Cancer | B44.. | Malignant neoplasm of ovary and other uterine adnexa |
| Cancer | ZV102 | [V]Personal history of malig neop other intrathoracic organ |
| Cancer | B514. | Malignant neoplasm of occipital lobe |
| Cancer | B493. | Malignant neoplasm of anterior wall of urinary bladder |
| Cancer | B020. | Malignant neoplasm of parotid gland |
| Cancer | B504. | Malignant neoplasm of cornea |
| Cancer | B143. | Malignant neoplasm of anus unspecified |
| Cancer | B6158 | Hodgkin's mixed cellularity of lymph nodes of multiple sites |
| Cancer | B3250 | Malignant melanoma of axilla |
| Cancer | B1420 | Malignant neoplasm of cloacogenic zone |
| Cancer | B5232 | Malignant neoplasm of spinal pia mater |
| Cancer | B002z | Malignant neoplasm of upper lip, inner aspect NOS |
| Cancer | B6164 | Hodgkin's lymphocytic depletion lymph nodes axilla and arm |
| Cancer | B221z | Malignant neoplasm of main bronchus NOS |
| Cancer | B3112 | Malignant neoplasm of connective and soft tissue of fore-arm |
| Cancer | B55.. | Malignant neoplasm of other and ill-defined sites |
| Cancer | B340. | Malignant neoplasm of nipple and areola of female breast |
| Cancer | B335A | Malignant neoplasm of skin of scapular region |
| Cancer | B6243 | Leukaemic reticuloend of intra-abdominal lymph nodes |
| Cancer | B062. | Malignant neoplasm of tonsillar pillar |
| Cancer | B6003 | Reticulosarcoma of intra-abdominal lymph nodes |
| Cancer | B624z | Leukaemic reticuloendotheliosis NOS |
| Cancer | B337. | Malignant neoplasm of skin of lower limb and hip |
| Cancer | B3070 | Malignant neoplasm of femur |
| Cancer | B339. | Dermatofibrosarcoma protuberans |
| Cancer | B0111 | Malignant neoplasm of midline of tongue |
| Cancer | B3373 | Malignant neoplasm of skin of popliteal fossa area |
| Cancer | B3240 | Malignant melanoma of scalp |
| Cancer | B443. | Malignant neoplasm of parametrium |
| Cancer | B3235 | Malignant melanoma of temple |
| Cancer | ByuD9 | [X]Other leukaemia of unspecified cell type |
| Cancer | B627X | Diffuse non-Hodgkin's lymphoma, unspecified |
| Cancer | B5243 | Malignant neoplasm of peripheral nerve of thorax |
| Cancer | B3006 | Malignant neoplasm of parietal bone |
| Cancer | B5172 | Malignant neoplasm of midbrain |
| Cancer | B21z. | Malignant neoplasm of larynx NOS |
| Cancer | B6272 | Follicular non-Hodgkin's large cell lymphoma |
| Cancer | B007. | Malignant neoplasm of lip, unspecified |
| Cancer | B470z | Malignant neoplasm of undescended testis NOS |
| Cancer | B224z | Malignant neoplasm of lower lobe, bronchus or lung NOS |
| Cancer | B3330 | Malignant neoplasm of skin of cheek, external |
| Cancer | B347. | Malignant neoplasm, overlapping lesion of breast |
| Cancer | B33.. | Other malignant neoplasm of skin |
| Cancer | B3270 | Malignant melanoma of hip |
| Cancer | B6002 | Reticulosarcoma of intrathoracic lymph nodes |
| Cancer | Byu1. | [X]Malignant neoplasm of digestive organs |
| Cancer | ByuA3 | [X]Malig neopl, overlap lesion brain & other part of CNS |
| Cancer | B151z | Malignant neoplasm of intrahepatic bile ducts NOS |
| Cancer | B673. | Mast cell leukaemia |
| Cancer | B43.. | Malignant neoplasm of body of uterus |
| Cancer | B100. | Malignant neoplasm of cervical oesophagus |
| Cancer | B1503 | Hepatocellular carcinoma |
| Cancer | B6233 | Malignant histiocytosis of intra-abdominal lymph nodes |
| Cancer | B4310 | Malignant neoplasm of lower uterine segment |
| Cancer | B612. | Hodgkin's sarcoma |
| Cancer | B334. | Malignant neoplasm of scalp and skin of neck |
| Cancer | B63z. | Immunoproliferative neoplasm or myeloma NOS |
| Cancer | B6130 | Hodgkin's, lymphocytic-histiocytic predominance unspec site |
| Cancer | B52X. | Malignant neoplasm of meninges, unspecified |
| Cancer | B41y. | Malignant neoplasm of other site of cervix |
| Cancer | B49z. | Malignant neoplasm of urinary bladder NOS |
| Cancer | B5202 | Malignant neoplasm of acoustic nerve |
| Cancer | B3310 | Malignant neoplasm of canthus |
| Cancer | B132. | Malignant neoplasm of descending colon |
| Cancer | B333z | Malignant neoplasm skin other and unspec part of face NOS |
| Cancer | B34yz | Malignant neoplasm of other site of female breast NOS |
| Cancer | B482. | Malignant neoplasm of body of penis |
| Cancer | Byu11 | [X]Other specified carcinomas of liver |
| Cancer | B06y. | Malignant neoplasm of oropharynx, other specified sites |
| Cancer | B3251 | Malignant melanoma of breast |
| Cancer | B66y. | Other monocytic leukaemia |
| Cancer | B03z. | Malignant neoplasm of gum NOS |
| Cancer | B205. | Malignant neoplasm of sphenoidal sinus |
| Cancer | B34z. | Malignant neoplasm of female breast NOS |
| Cancer | B500z | Malignant neoplasm of eyeball NOS |
| Cancer | B63.. | Multiple myeloma and immunoproliferative neoplasms |
| Cancer | Byu81 | [X]Malignant neoplasm/overlapping lesion/male genital organs |
| Cancer | B5504 | Malignant neoplasm of neck NOS |
| Cancer | B117. | Malignant neoplasm, overlapping lesion of stomach |
| Cancer | B230. | Malignant neoplasm of parietal pleura |
| Cancer | B6140 | Hodgkin's disease, nodular sclerosis of unspecified site |
| Cancer | B512. | Malignant neoplasm of temporal lobe |
| Cancer | B3056 | Malignant neoplasm of carpal bone - capitate |
| Cancer | B431. | Malignant neoplasm of isthmus of uterine body |
| Cancer | B6300 | Malignant plasma cell neoplasm, extramedullary plasmacytoma |
| Cancer | B1500 | Primary carcinoma of liver |
| Cancer | B483. | Malignant neoplasm of penis, part unspecified |
| Cancer | B1101 | Malignant neoplasm of cardio-oesophageal junction of stomach |
| Cancer | B052. | Malignant neoplasm of hard palate |
| Cancer | B651. | Chronic myeloid leukaemia |
| Cancer | B623z | Malignant histiocytosis NOS |
| Cancer | B6531 | Granulocytic sarcoma |
| Cancer | B452. | Malignant neoplasm of labia minora |
| Cancer | B35.. | Malignant neoplasm of male breast |
| Cancer | B3254 | Malignant melanoma of perianal skin |
| Cancer | B641. | Chronic lymphoid leukaemia |
| Cancer | B3059 | Malignant neoplasm of second metacarpal bone |
| Cancer | B626. | Malignant mast cell tumours |
| Cancer | B0023 | Malignant neoplasm of upper lip, oral aspect |
| Cancer | B64y2 | Adult T-cell leukaemia |
| Cancer | B300C | Malignant neoplasm of vomer |
| Cancer | B3120 | Malignant neoplasm of connective and soft tissue of hip |
| Cancer | B23z. | Malignant neoplasm of pleura NOS |
| Cancer | B66.. | Monocytic leukaemia |
| Cancer | B61z0 | Hodgkin's disease NOS, unspecified site |
| Cancer | B6162 | Hodgkin's lymphocytic depletion of intrathoracic lymph nodes |
| Cancer | B3341 | Malignant neoplasm of skin of neck |
| Cancer | B2201 | Malignant neoplasm of mucosa of trachea |
| Cancer | B6235 | Malignant histiocytosis of lymph nodes inguinal and leg |
| Cancer | B5530 | Malignant neoplasm of inguinal region NOS |
| Cancer | B173. | Malignant neoplasm of pancreatic duct |
| Cancer | Byu90 | [X]Malignant neoplasm of urinary organ, unspecified |
| Cancer | B6205 | Nodular lymphoma of lymph nodes of inguinal region and leg |
| Cancer | B5120 | Malignant neoplasm of hippocampus |
| Cancer | B3071 | Malignant neoplasm of fibula |
| Cancer | B6111 | Hodgkin's granuloma of lymph nodes of head, face and neck |
| Cancer | B521z | Malignant neoplasm of cerebral meninges NOS |
| Cancer | B68y. | Other leukaemia of unspecified cell type |
| Cancer | B20y. | Malig neop other site nasal cavity, middle ear and sinuses |
| Cancer | B0031 | Malignant neoplasm of lower lip, frenulum |
| Cancer | B0731 | Malignant neoplasm of nasopharyngeal soft palate surface |
| Cancer | B2410 | Malignant neoplasm of endocardium |
| Cancer | B11y. | Malignant neoplasm of other specified site of stomach |
| Cancer | B08.. | Malignant neoplasm of hypopharynx |
| Cancer | B32y0 | Overlapping malignant melanoma of skin |
| Cancer | B32z. | Malignant melanoma of skin NOS |
| Cancer | B650. | Acute myeloid leukaemia |
| Cancer | B651z | Chronic myeloid leukaemia NOS |
| Cancer | B1100 | Malignant neoplasm of cardiac orifice of stomach |
| Cancer | B2240 | Malignant neoplasm of lower lobe bronchus |
| Cancer | B3353 | Malignant neoplasm of skin of abdominal wall |
| Cancer | ByuDC | [X]Diffuse non-Hodgkin's lymphoma, unspecified |
| Cancer | Byu5A | [X]Malignant neoplasm overlapping lesion of skin |
| Cancer | Byu32 | [X]Malignant neoplasm/overlap lesion/bone+articulr cartilage |
| Cancer | B3105 | Malignant neoplasm soft tissues of cervical spine |
| Cancer | B18y2 | Malignant neoplasm of mesorectum |
| Cancer | Byu21 | [X]Malignant neoplasm/overlap lesion/heart,mediastinm+pleura |
| Cancer | B310. | Malig neop of connective and soft tissue head, face and neck |
| Cancer | B01y. | Malignant neoplasm of other sites of tongue |
| Cancer | B3334 | Malignant neoplasm of skin of nose (external) |
| Cancer | B07.. | Malignant neoplasm of nasopharynx |
| Cancer | B23y. | Malignant neoplasm of other specified pleura |
| Cancer | B420. | Choriocarcinoma |
| Cancer | B674. | Acute panmyelosis |
| Cancer | B6214 | Mycosis fungoides of lymph nodes of axilla and upper limb |
| Cancer | B6267 | Mast cell malignancy of spleen |
| Cancer | ByuDE | [X]Unspecified B-cell non-Hodgkin's lymphoma |
| Cancer | B140. | Malignant neoplasm of rectosigmoid junction |
| Cancer | B1511 | Malignant neoplasm of interlobular biliary canals |
| Cancer | B491. | Malignant neoplasm of dome of urinary bladder |
| Cancer | B0641 | Malignant neoplasm of glossoepiglottic fold |
| Cancer | B510z | Malignant neoplasm of cerebrum NOS |
| Cancer | ZV107 | [V]Personal history other lymphatic/haematopoietic neoplasm |
| Cancer | B5512 | Malignant neoplasm of intrathoracic site NOS |
| Cancer | B6210 | Mycosis fungoides of unspecified site |
| Cancer | B4A2. | Malignant neoplasm of ureter |
| Cancer | B6112 | Hodgkin's granuloma of intrathoracic lymph nodes |
| Cancer | B6102 | Hodgkin's paragranuloma of intrathoracic lymph nodes |
| Cancer | B451z | Malignant neoplasm of labia majora NOS |
| Cancer | B623. | Malignant histiocytosis |
| Cancer | B4702 | Seminoma of undescended testis |
| Cancer | B5105 | Malignant neoplasm of thalamus |
| Cancer | B03y. | Malignant neoplasm of other sites of gum |
| Cancer | B6274 | Diffuse non-Hodgkin's small cleaved cell (diffuse) lymphoma |
| Cancer | B3101 | Malignant neoplasm of soft tissue of face |
| Cancer | B3379 | Malignant neoplasm of skin of great toe |
| Cancer | B62y2 | Malignant lymphoma NOS of intrathoracic lymph nodes |
| Cancer | B672. | Megakaryocytic leukaemia |
| Cancer | B6131 | Hodgkin's, lymphocytic-histiocytic pred of head, face, neck |
| Cancer | B444. | Malignant neoplasm of round ligament |
| Cancer | B120. | Malignant neoplasm of duodenum |
| Cancer | Byu53 | [X]Kaposi's sarcoma, unspecified |
| Cancer | B6116 | Hodgkin's granuloma of intrapelvic lymph nodes |
| Cancer | B15.. | Malignant neoplasm of liver and intrahepatic bile ducts |
| Cancer | B501. | Malignant neoplasm of orbit |
| Cancer | B6261 | Mast cell malignancy of lymph nodes of head, face and neck |
| Cancer | B0001 | Malignant neoplasm of upper lip, lipstick area |
| Cancer | B5010 | Malignant neoplasm of connective tissue of orbit |
| Cancer | B2230 | Malignant neoplasm of middle lobe bronchus |
| Cancer | B691. | Chronic myelomonocytic leukaemia |
| Cancer | B1110 | Malignant neoplasm of prepylorus of stomach |
| Cancer | B481. | Malignant neoplasm of glans penis |
| Cancer | Byu24 | [X]Malignant neoplasm/ill-defined sites within resp system |
| Cancer | B6133 | Hodgkin's, lymphocytic-histiocytic pred intra-abdominal node |
| Cancer | B553. | Malignant neoplasm of pelvis |
| Cancer | B.... | Neoplasms |
| Cancer | B3350 | Malignant neoplasm of skin of axillary fold |
| Cancer | B511. | Malignant neoplasm of frontal lobe |
| Cancer | B232. | Mesothelioma of pleura |
| Cancer | B3152 | Malignant neoplasm of connective and soft tissue of perineum |
| Cancer | B0033 | Malignant neoplasm of lower lip, oral aspect |
| Cancer | B030. | Malignant neoplasm of upper gum |
| Cancer | B3231 | Malignant melanoma of chin |
| Cancer | B540z | Malignant neoplasm of adrenal gland NOS |
| Cancer | B3271 | Malignant melanoma of thigh |
| Cancer | B0720 | Malignant neoplasm of pharyngeal recess |
| Cancer | Byu70 | [X]Malignant neoplasm of uterine adnexa, unspecified |
| Cancer | B308C | Malignant neoplasm of fifth metatarsal bone |
| Cancer | B4Ay0 | Malignant neoplasm of overlapping lesion of urinary organs |
| Cancer | ByuE0 | [X]Malignant neoplasms/independent(primary)multiple sites |
| Cancer | B6213 | Mycosis fungoides of intra-abdominal lymph nodes |
| Cancer | B543. | Malignant neoplasm of pineal gland |
| Cancer | B6113 | Hodgkin's granuloma of intra-abdominal lymph nodes |
| Cancer | B5230 | Malignant neoplasm of spinal dura mater |
| Cancer | B3100 | Malignant neoplasm of soft tissue of head |
| Cancer | B3375 | Malignant neoplasm of skin of ankle |
| Cancer | B11yz | Malignant neoplasm of other specified site of stomach NOS |
| Cancer | B136. | Malignant neoplasm of ascending colon |
| Cancer | B50y. | Malignant neoplasm of other specified site of eye |
| Cancer | B3063 | Malignant neoplasm of sacral vertebra |
| Cancer | B46.. | Malignant neoplasm of prostate |
| Cancer | B134. | Malignant neoplasm of caecum |
| Cancer | B3043 | Malignant neoplasm of radius |
| Cancer | B62z7 | Unspec malig neop lymphoid/histiocytic of spleen |
| Cancer | Byu8. | [X]Malignant neoplasm of male genital organs |
| Cancer | B17z. | Malignant neoplasm of pancreas NOS |
| Cancer | B141. | Malignant neoplasm of rectum |
| Cancer | B5104 | Malignant neoplasm of hypothalamus |
| Cancer | B62zz | Lymphoid and histiocytic malignancy NOS |
| Cancer | B3084 | Malignant neoplasm of intermediate cuneiform |
| Cancer | B18.. | Malignant neoplasm of retroperitoneum and peritoneum |
| Cancer | B314z | Malig neop of connective and soft tissue of abdomen NOS |
| Cancer | B11z. | Malignant neoplasm of stomach NOS |
| Cancer | B111. | Malignant neoplasm of pylorus of stomach |
| Cancer | B5500 | Malignant neoplasm of head NOS |
| Cancer | B48.. | Malignant neoplasm of penis and other male genital organs |
| Cancer | B6023 | Burkitt's lymphoma of intra-abdominal lymph nodes |
| Cancer | B3086 | Malignant neoplasm of cuboid |
| Cancer | B3358 | Malignant neoplasm of skin of buttock |
| Cancer | B306z | Malignant neoplasm of pelvis, sacrum or coccyx NOS |
| Cancer | B06y0 | Malignant neoplasm of branchial cleft |
| Cancer | B0602 | Malignant neoplasm of overlapping lesion of tonsil |
| Cancer | B241. | Malignant neoplasm of heart |
| Cancer | B681. | Chronic leukaemia NOS |
| Cancer | B2132 | Malignant neoplasm of cuneiform cartilage |
| Cancer | B3363 | Malignant neoplasm of skin of hand |
| Cancer | B3371 | Malignant neoplasm of skin of thigh |
| Cancer | B3275 | Malignant melanoma of ankle |
| Cancer | B0z0. | Malignant neoplasm of pharynx unspecified |
| Cancer | B553z | Malignant neoplasm of pelvis NOS |
| Cancer | B345. | Malignant neoplasm of lower-outer quadrant of female breast |
| Cancer | Byu42 | [X]Oth malignant neoplasm/skin of oth+unspecfd parts of face |
| Cancer | B2414 | Mesothelioma of pericardium |
| Cancer | B64y0 | Aleukaemic lymphoid leukaemia |
| Cancer | B073z | Malignant neoplasm of anterior wall of nasopharynx NOS |
| Cancer | B2131 | Malignant neoplasm of cricoid cartilage |
| Cancer | B0131 | Malignant neoplasm of frenulum linguae |
| Cancer | B66y0 | Aleukaemic monocytic leukaemia |
| Cancer | B4500 | Malignant neoplasm of Gartner's duct |
| Cancer | B0z.. | Malig neop other/ill-defined sites lip, oral cavity, pharynx |
| Cancer | ByuC0 | [X]Malignant neoplasm of other specified sites |
| Cancer | B3041 | Malignant neoplasm of acromion |
| Cancer | B3042 | Malignant neoplasm of humerus |
| Cancer | B451. | Malignant neoplasm of labia majora |
| Cancer | B321. | Malignant melanoma of eyelid including canthus |
| Cancer | B201. | Malig neop auditory tube, middle ear and mastoid air cells |
| Cancer | B2413 | Malignant neoplasm of pericardium |
| Cancer | B3053 | Malignant neoplasm of carpal bone - pisiform |
| Cancer | B3007 | Malignant neoplasm of sphenoid bone |
| Cancer | B305D | Malignant neoplasm of phalanges of hand |
| Cancer | B00z1 | Malignant neoplasm of lip, unspecified, lipstick area |
| Cancer | B0621 | Malignant neoplasm of glossopalatine fold |
| Cancer | B521. | Malignant neoplasm of cerebral meninges |
| Cancer | B6134 | Hodgkin's, lymphocytic-histiocytic pred axilla and arm |
| Cancer | B6200 | Nodular lymphoma of unspecified site |
| Cancer | B3072 | Malignant neoplasm of tibia |
| Cancer | B083. | Malignant neoplasm of posterior pharynx |
| Cancer | B226. | Mesothelioma |
| Cancer | B507. | Malignant neoplasm of lacrimal duct |
| Cancer | Byu50 | [X]Mesothelioma of other sites |
| Cancer | B3083 | Malignant neoplasm of medial cuneiform |
| Cancer | B6512 | Chronic neutrophilic leukaemia |
| Cancer | B332. | Malignant neoplasm skin of ear and external auricular canal |
| Cancer | B3052 | Malignant neoplasm of carpal bone - triquetrum |
| Cancer | B4Az. | Malignant neoplasm of kidney or urinary organs NOS |
| Cancer | B3370 | Malignant neoplasm of skin of hip |
| Cancer | B135. | Malignant neoplasm of appendix |
| Cancer | B072. | Malignant neoplasm of lateral wall of nasopharynx |
| Cancer | B6017 | Lymphosarcoma of spleen |
| Cancer | B206. | Malignant neoplasm, overlapping lesion of accessory sinuses |
| Cancer | B6230 | Malignant histiocytosis of unspecified site |
| Cancer | B4711 | Teratoma of descended testis |
| Cancer | B323z | Malignant melanoma of face NOS |
| Cancer | B053. | Malignant neoplasm of soft palate |
| Cancer | ByuDA | [X]Oth spcf mal neoplsm/lymphoid,haematopoietic+rltd tissue |
| Cancer | B0511 | Malignant neoplasm of lower buccal sulcus |
| Cancer | B3151 | Malig neop of connective and soft tissue of inguinal region |
| Cancer | B0000 | Malignant neoplasm of upper lip, external |
| Cancer | B3002 | Malignant neoplasm of malar bone |
| Cancer | B3044 | Malignant neoplasm of ulna |
| Cancer | B5103 | Malignant neoplasm of globus pallidus |
| Cancer | B114. | Malignant neoplasm of body of stomach |
| Cancer | Byu80 | [X]Malignant neoplasm/other specified male genital organs |
| Cancer | B340z | Malignant neoplasm of nipple or areola of female breast NOS |
| Cancer | B507z | Malignant neoplasm of lacrimal duct NOS |
| Cancer | B243. | Malignant neoplasm of posterior mediastinum |
| Cancer | B131. | Malignant neoplasm of transverse colon |
| Cancer | Byu23 | [X]Malignant neopl/overlapping les/resp+intrathoracic organs |
| Cancer | B17y0 | Malignant neoplasm of ectopic pancreatic tissue |
| Cancer | B124. | Malignant neoplasm, overlapping lesion of small intestine |
| Cancer | B081. | Malignant neoplasm of pyriform sinus |
| Cancer | B5212 | Malignant neoplasm of cerebral pia mater |
| Cancer | B1800 | Malignant neoplasm of periadrenal tissue |
| Cancer | B3126 | Malig neop of connective and soft tissue of great toe |
| Cancer | B24.. | Malignant neoplasm of thymus, heart and mediastinum |
| Cancer | Byu10 | [X]Other sarcomas of the liver |
| Cancer | B0721 | Malignant neoplasm of opening of auditory tube |
| Cancer | B6215 | Mycosis fungoides of lymph nodes of inguinal region and leg |
| Cancer | B3335 | Malignant neoplasm of skin of temple |
| Cancer | B5400 | Malignant neoplasm of adrenal cortex |
| Cancer | B151. | Malignant neoplasm of intrahepatic bile ducts |
| Cancer | Byu22 | [X]Malignant neoplasm/upper resp tract, part unspecified |
| Cancer | B051z | Malignant neoplasm of vestibule of mouth NOS |
| Cancer | B6271 | Follicular non-Hodg mixed sml cleavd & lge cell lymphoma |
| Cancer | B3256 | Malignant melanoma of umbilicus |
| Cancer | B0032 | Malignant neoplasm of lower lip, mucosa |
| Cancer | B6124 | Hodgkin's sarcoma of lymph nodes of axilla and upper limb |
| Cancer | B0100 | Malignant neoplasm of base of tongue dorsal surface |
| Cancer | B69.. | Myelomonocytic leukaemia |
| Dementia | Eu06z | [X]Unspec organ personality behav disorder brain dam dysfunc |
| Dementia | E0041 | Arteriosclerotic dementia with delirium |
| Dementia | Eu041 | [X]Delirium superimposed on dementia |
| Dementia | Eu012 | [X]Subcortical vascular dementia |
| Dementia | E00.. | Senile and presenile organic psychotic conditions |
| Dementia | E00y. | Other senile and presenile organic psychoses |
| Dementia | Eu02y | [X]Dementia in other specified diseases classif elsewhere |
| Dementia | E0021 | Senile dementia with depression |
| Dementia | E0012 | Presenile dementia with paranoia |
| Dementia | Eu020 | [X]Dementia in Pick's disease |
| Dementia | Eu01. | [X]Vascular dementia |
| Dementia | Eu052 | [X]Organic delusional [schizophrenia-like] disorder |
| Dementia | E0043 | Arteriosclerotic dementia with depression |
| Dementia | Eu00z | [X]Dementia in Alzheimer's disease, unspecified |
| Dementia | Eu00. | [X]Dementia in Alzheimer's disease |
| Dementia | Eu04z | [X]Delirium, unspecified |
| Dementia | Eu001 | [X]Dementia in Alzheimer's disease with late onset |
| Dementia | E003. | Senile dementia with delirium |
| Dementia | Eu058 | [X]Cognitive communication disorder |
| Dementia | E0013 | Presenile dementia with depression |
| Dementia | Eu056 | [X]Organic emotionally labile [asthenic] disorder |
| Dementia | E002z | Senile dementia with depressive or paranoid features NOS |
| Dementia | E002. | Senile dementia with depressive or paranoid features |
| Dementia | Eu000 | [X]Dementia in Alzheimer's disease with early onset |
| Dementia | Eu01y | [X]Other vascular dementia |
| Dementia | Eu0.. | [X]Organic, including symptomatic, mental disorders |
| Dementia | Eu023 | [X]Dementia in Parkinson's disease |
| Dementia | Eu062 | [X]Postconcussional syndrome |
| Dementia | Eu022 | [X]Dementia in Huntington's disease |
| Dementia | Eu05z | [X]Unspec mental disorder brain damag/dysfunction/physcal dr |
| Dementia | E001z | Presenile dementia NOS |
| Dementia | Eu04y | [X]Other delirium |
| Dementia | E0020 | Senile dementia with paranoia |
| Dementia | E0040 | Uncomplicated arteriosclerotic dementia |
| Dementia | Eu0z. | [X]Unspecified organic or symptomatic mental disorder |
| Dementia | Eu040 | [X]Delirium not superimposed on dementia, so described |
| Dementia | Eu025 | [X]Lewy body dementia |
| Dementia | E001. | Presenile dementia |
| Dementia | E0010 | Uncomplicated presenile dementia |
| Dementia | E004z | Arteriosclerotic dementia NOS |
| Dementia | Eu060 | [X]Organic personality disorder |
| Dementia | Eu024 | [X]Dementia in human immunodef virus [HIV] disease |
| Dementia | Eu055 | [X]Organic dissociative disorder |
| Dementia | Eu021 | [X]Dementia in Creutzfeldt-Jakob disease |
| Dementia | E0042 | Arteriosclerotic dementia with paranoia |
| Dementia | Eu050 | [X]Organic hallucinosis |
| Dementia | Eu051 | [X]Organic catatonic disorder |
| Dementia | E0011 | Presenile dementia with delirium |
| Dementia | Eu010 | [X]Vascular dementia of acute onset |
| Dementia | Eu057 | [X]Mild cognitive disorder |
| Dementia | Eu03. | [X]Organic amnesic synd not induced alc/oth psychoact subs |
| Dementia | Eu06. | [X]Personality and behav disorder brain dis dam and dysfunct |
| Dementia | Eu011 | [X]Multi-infarct dementia |
| Dementia | Eu02. | [X]Dementia in other diseases classified elsewhere |
| Dementia | Eu02z | [X] Unspecified dementia |
| Dementia | Eu061 | [X]Postencephalitic syndrome |
| Dementia | Eu06y | [X]Oth organ personality behav disorders brain dis dam dysf |
| Dementia | Eu05. | [X]Oth mental disorder brain damag/dysfunction/physical disr |
| Dementia | Eu04. | [X]Delirium, not induced by alcohol+other psychoactive subs |
| Dementia | Eu002 | [X]Dementia in Alzheimer's dis, atypical or mixed type |
| Dementia | E004. | Arteriosclerotic dementia |
| Dementia | Eu05y | [X]Oth sp mental disord brain damag/dysfunction/physcal disd |
| Dementia | Eu053 | [X]Organic mood [affective] disorders |
| Dementia | E000. | Uncomplicated senile dementia |
| Dementia | E00z. | Senile or presenile psychoses NOS |
| Dementia | Eu054 | [X]Organic anxiety disorder |
| Dementia | Eu01z | [X]Vascular dementia, unspecified |
| Dementia | Eu013 | [X]Mixed cortical and subcortical vascular dementia |
| Diabetes | C1094 | Non-insulin dependent diabetes mellitus with ulcer |
| Diabetes | C10EN | Type 1 diabetes mellitus with ketoacidotic coma |
| Diabetes | 66AJ2 | Loss of hypoglycaemic warning |
| Diabetes | C10E9 | Type 1 diabetes mellitus maturity onset |
| Diabetes | C10F0 | Type 2 diabetes mellitus with renal complications |
| Diabetes | C10y0 | Diabetes mellitus, juvenile, + other specified manifestation |
| Diabetes | C105. | Diabetes mellitus with ophthalmic manifestation |
| Diabetes | C1083 | Insulin dependent diabetes mellitus with multiple complicatn |
| Diabetes | C10EK | Type 1 diabetes mellitus with persistent proteinuria |
| Diabetes | 66AJ4 | Hypoglycaemic warning absent |
| Diabetes | C1097 | Non-insulin dependent diabetes mellitus - poor control |
| Diabetes | C1080 | Insulin-dependent diabetes mellitus with renal complications |
| Diabetes | 66AJ1 | Brittle diabetes |
| Diabetes | C103. | Diabetes mellitus with ketoacidotic coma |
| Diabetes | C10E1 | Type 1 diabetes mellitus with ophthalmic complications |
| Diabetes | C1050 | Diabetes mellitus, juvenile type, + ophthalmic manifestation |
| Diabetes | C1096 | Non-insulin-dependent diabetes mellitus with retinopathy |
| Diabetes | C1089 | Insulin dependent diabetes maturity onset |
| Diabetes | C10A. | Malnutrition-related diabetes mellitus |
| Diabetes | C10L. | Fibrocalculous pancreatopathy |
| Diabetes | C10z. | Diabetes mellitus with unspecified complication |
| Diabetes | C107. | Diabetes mellitus with peripheral circulatory disorder |
| Diabetes | C10E5 | Type 1 diabetes mellitus with ulcer |
| Diabetes | C10yz | Diabetes mellitus NOS with other specified manifestation |
| Diabetes | C1098 | Reaven's syndrome |
| Diabetes | C10N0 | Secondary diabetes mellitus without complication |
| Diabetes | C1091 | Non-insulin-dependent diabetes mellitus with ophthalm comps |
| Diabetes | C10FF | Type 2 diabetes mellitus with peripheral angiopathy |
| Diabetes | C10D. | Diabetes mellitus autosomal dominant type 2 |
| Diabetes | C1041 | Diabetes mellitus, adult onset, with renal manifestation |
| Diabetes | C10EP | Type 1 diabetes mellitus with exudative maculopathy |
| Diabetes | C10G0 | Secondary pancreatic diabetes mellitus without complication |
| Diabetes | C10EC | Type 1 diabetes mellitus with polyneuropathy |
| Diabetes | C10EM | Type 1 diabetes mellitus with ketoacidosis |
| Diabetes | C108. | Insulin dependent diabetes mellitus |
| Diabetes | C109A | Non-insulin dependent diabetes mellitus with mononeuropathy |
| Diabetes | C10zz | Diabetes mellitus NOS with unspecified complication |
| Diabetes | C104z | Diabetes mellitus with nephropathy NOS |
| Diabetes | C103z | Diabetes mellitus NOS with ketoacidotic coma |
| Diabetes | C10EQ | Type 1 diabetes mellitus with gastroparesis |
| Diabetes | C1070 | Diabetes mellitus, juvenile +peripheral circulatory disorder |
| Diabetes | C10A1 | Malnutrition-related diabetes mellitus with ketoacidosis |
| Diabetes | C10F6 | Type 2 diabetes mellitus with retinopathy |
| Diabetes | C10E3 | Type 1 diabetes mellitus with multiple complications |
| Diabetes | C10N. | Secondary diabetes mellitus |
| Diabetes | F3721 | Chronic painful diabetic neuropathy |
| Diabetes | C10A5 | Malnutritn-relat diabetes melitus wth periph circul complctn |
| Diabetes | C10M. | Lipoatrophic diabetes mellitus |
| Diabetes | C1087 | Insulin dependent diabetes mellitus with retinopathy |
| Diabetes | C107y | Other specified diabetes mellitus with periph circ comps |
| Diabetes | C108D | Insulin dependent diabetes mellitus with nephropathy |
| Diabetes | C10FB | Type 2 diabetes mellitus with polyneuropathy |
| Diabetes | C10FK | Hyperosmolar non-ketotic state in type 2 diabetes mellitus |
| Diabetes | C10FG | Type 2 diabetes mellitus with arthropathy |
| Diabetes | C10F7 | Type 2 diabetes mellitus - poor control |
| Diabetes | C10P1 | Type II diabetes mellitus in remission |
| Diabetes | C10H. | Diabetes mellitus induced by non-steroid drugs |
| Diabetes | C1031 | Diabetes mellitus, adult onset, with ketoacidotic coma |
| Diabetes | C1051 | Diabetes mellitus, adult onset, + ophthalmic manifestation |
| Diabetes | C109J | Insulin treated Type 2 diabetes mellitus |
| Diabetes | C10EF | Type 1 diabetes mellitus with diabetic cataract |
| Diabetes | C108B | Insulin dependent diabetes mellitus with mononeuropathy |
| Diabetes | C10FH | Type 2 diabetes mellitus with neuropathic arthropathy |
| Diabetes | C10z0 | Diabetes mellitus, juvenile type, + unspecified complication |
| Diabetes | C1095 | Non-insulin dependent diabetes mellitus with gangrene |
| Diabetes | C104. | Diabetes mellitus with renal manifestation |
| Diabetes | 66AJ. | Diabetic - poor control |
| Diabetes | C1090 | Non-insulin-dependent diabetes mellitus with renal comps |
| Diabetes | C1071 | Diabetes mellitus, adult, + peripheral circulatory disorder |
| Diabetes | C1086 | Insulin dependent diabetes mellitus with gangrene |
| Diabetes | C1085 | Insulin dependent diabetes mellitus with ulcer |
| Diabetes | C10y. | Diabetes mellitus with other specified manifestation |
| Diabetes | C100. | Diabetes mellitus with no mention of complication |
| Diabetes | C101z | Diabetes mellitus NOS with ketoacidosis |
| Diabetes | C108F | Insulin dependent diabetes mellitus with diabetic cataract |
| Diabetes | C101y | Other specified diabetes mellitus with ketoacidosis |
| Diabetes | C10z1 | Diabetes mellitus, adult onset, + unspecified complication |
| Diabetes | C10B. | Diabetes mellitus induced by steroids |
| Diabetes | C1040 | Diabetes mellitus, juvenile type, with renal manifestation |
| Diabetes | C10A3 | Malnutrit-related diabetes mellitus wth ophthalmic complicat |
| Diabetes | 66AJ3 | Recurrent severe hypos |
| Diabetes | C10F. | Type 2 diabetes mellitus |
| Diabetes | C10E6 | Type 1 diabetes mellitus with gangrene |
| Diabetes | C109H | Non-insulin dependent d m with neuropathic arthropathy |
| Diabetes | C108H | Insulin dependent diabetes mellitus with arthropathy |
| Diabetes | C1088 | Insulin dependent diabetes mellitus - poor control |
| Diabetes | C10EJ | Type 1 diabetes mellitus with neuropathic arthropathy |
| Diabetes | C100z | Diabetes mellitus NOS with no mention of complication |
| Diabetes | C10F3 | Type 2 diabetes mellitus with multiple complications |
| Diabetes | C109K | Hyperosmolar non-ketotic state in type 2 diabetes mellitus |
| Diabetes | C10E0 | Type 1 diabetes mellitus with renal complications |
| Diabetes | C10E. | Type 1 diabetes mellitus |
| Diabetes | C10zy | Other specified diabetes mellitus with unspecified comps |
| Diabetes | C109F | Non-insulin-dependent d m with peripheral angiopath |
| Diabetes | C10FM | Type 2 diabetes mellitus with persistent microalbuminuria |
| Diabetes | C10FC | Type 2 diabetes mellitus with nephropathy |
| Diabetes | 66AJ0 | Chronic hyperglycaemia |
| Diabetes | C1081 | Insulin-dependent diabetes mellitus with ophthalmic comps |
| Diabetes | C10FN | Type 2 diabetes mellitus with ketoacidosis |
| Diabetes | C10A0 | Malnutrition-related diabetes mellitus with coma |
| Diabetes | C10A2 | Malnutrition-related diabetes mellitus with renal complicatn |
| Diabetes | C10F1 | Type 2 diabetes mellitus with ophthalmic complications |
| Diabetes | F3722 | Asymptomatic diabetic neuropathy |
| Diabetes | C10F9 | Type 2 diabetes mellitus without complication |
| Diabetes | C1082 | Insulin-dependent diabetes mellitus with neurological comps |
| Diabetes | C10yy | Other specified diabetes mellitus with other spec comps |
| Diabetes | C109E | Non-insulin depend diabetes mellitus with diabetic cataract |
| Diabetes | C10P. | Diabetes mellitus in remission |
| Diabetes | C10E8 | Type 1 diabetes mellitus - poor control |
| Diabetes | C108G | Insulin dependent diab mell with peripheral angiopathy |
| Diabetes | C10E4 | Unstable type 1 diabetes mellitus |
| Diabetes | C10K. | Type A insulin resistance |
| Diabetes | C1092 | Non-insulin-dependent diabetes mellitus with neuro comps |
| Diabetes | C10EG | Type 1 diabetes mellitus with peripheral angiopathy |
| Diabetes | C1010 | Diabetes mellitus, juvenile type, with ketoacidosis |
| Diabetes | C10G. | Secondary pancreatic diabetes mellitus |
| Diabetes | C109B | Non-insulin dependent diabetes mellitus with polyneuropathy |
| Diabetes | C10FQ | Type 2 diabetes mellitus with exudative maculopathy |
| Diabetes | C10K0 | Type A insulin resistance without complication |
| Diabetes | C10C. | Diabetes mellitus autosomal dominant |
| Diabetes | C109C | Non-insulin dependent diabetes mellitus with nephropathy |
| Diabetes | C10ED | Type 1 diabetes mellitus with nephropathy |
| Diabetes | C10EE | Type 1 diabetes mellitus with hypoglycaemic coma |
| Diabetes | C1001 | Diabetes mellitus, adult onset, no mention of complication |
| Diabetes | C10N1 | Cystic fibrosis related diabetes mellitus |
| Diabetes | C1072 | Diabetes mellitus, adult with gangrene |
| Diabetes | C1099 | Non-insulin-dependent diabetes mellitus without complication |
| Diabetes | F3720 | Acute painful diabetic neuropathy |
| Diabetes | C10EL | Type 1 diabetes mellitus with persistent microalbuminuria |
| Diabetes | C10A6 | Malnutrition-related diabetes mellitus with multiple comps |
| Diabetes | C108J | Insulin dependent diab mell with neuropathic arthropathy |
| Diabetes | C10y1 | Diabetes mellitus, adult, + other specified manifestation |
| Diabetes | C109G | Non-insulin dependent diabetes mellitus with arthropathy |
| Diabetes | C10A4 | Malnutrition-related diabetes mellitus wth neuro complicatns |
| Diabetes | C10B0 | Steroid induced diabetes mellitus without complication |
| Diabetes | C107z | Diabetes mellitus NOS with peripheral circulatory disorder |
| Diabetes | C108E | Insulin dependent diabetes mellitus with hypoglycaemic coma |
| Diabetes | C109D | Non-insulin dependent diabetes mellitus with hypoglyca coma |
| Diabetes | C1011 | Diabetes mellitus, adult onset, with ketoacidosis |
| Diabetes | C10L0 | Fibrocalculous pancreatopathy without complication |
| Diabetes | C10F4 | Type 2 diabetes mellitus with ulcer |
| Diabetes | 1434. | H/O: diabetes mellitus |
| Diabetes | C10FD | Type 2 diabetes mellitus with hypoglycaemic coma |
| Diabetes | C10H0 | DM induced by non-steroid drugs without complication |
| Diabetes | C108z | Unspecified diabetes mellitus with multiple complications |
| Diabetes | C10FR | Type 2 diabetes mellitus with gastroparesis |
| Diabetes | C10A7 | Malnutrition-related diabetes mellitus without complications |
| Diabetes | C314. | Renal glycosuria |
| Diabetes | C105y | Other specified diabetes mellitus with ophthalmic complicatn |
| Diabetes | C1074 | NIDDM with peripheral circulatory disorder |
| Diabetes | C1073 | IDDM with peripheral circulatory disorder |
| Diabetes | C10FJ | Insulin treated Type 2 diabetes mellitus |
| Diabetes | C10FE | Type 2 diabetes mellitus with diabetic cataract |
| Diabetes | C108A | Insulin-dependent diabetes without complication |
| Diabetes | C10F8 | Reaven's syndrome |
| Diabetes | C108y | Other specified diabetes mellitus with multiple comps |
| Diabetes | C10FA | Type 2 diabetes mellitus with mononeuropathy |
| Diabetes | C10FL | Type 2 diabetes mellitus with persistent proteinuria |
| Diabetes | C10E7 | Type 1 diabetes mellitus with retinopathy |
| Diabetes | C10M0 | Lipoatrophic diabetes mellitus without complication |
| Diabetes | C10ER | Latent autoimmune diabetes mellitus in adult |
| Diabetes | C10P0 | Type I diabetes mellitus in remission |
| Diabetes | C104y | Other specified diabetes mellitus with renal complications |
| Diabetes | 66AJz | Diabetic - poor control NOS |
| Diabetes | C1084 | Unstable insulin dependent diabetes mellitus |
| Diabetes | C109. | Non-insulin dependent diabetes mellitus |
| Diabetes | C10FS | Maternally inherited diabetes mellitus |
| Diabetes | C10AW | Malnutrit-related diabetes mellitus with unspec complics |
| Diabetes | C1000 | Diabetes mellitus, juvenile type, no mention of complication |
| Diabetes | F372. | Polyneuropathy in diabetes |
| Diabetes | C10F2 | Type 2 diabetes mellitus with neurological complications |
| Diabetes | C10J. | Insulin autoimmune syndrome |
| Diabetes | C10EA | Type 1 diabetes mellitus without complication |
| Diabetes | C103y | Other specified diabetes mellitus with coma |
| Diabetes | C10F5 | Type 2 diabetes mellitus with gangrene |
| Diabetes | C10FP | Type 2 diabetes mellitus with ketoacidotic coma |
| Diabetes | C1093 | Non-insulin-dependent diabetes mellitus with multiple comps |
| Diabetes | C10EB | Type 1 diabetes mellitus with mononeuropathy |
| Diabetes | C108C | Insulin dependent diabetes mellitus with polyneuropathy |
| Diabetes | C10AX | Malnutrit-relat diabetes mellitus with other spec comps |
| Diabetes | C105z | Diabetes mellitus NOS with ophthalmic manifestation |
| Diabetes | C10EH | Type 1 diabetes mellitus with arthropathy |
| Diabetes | C1030 | Diabetes mellitus, juvenile type, with ketoacidotic coma |
| Diabetes | C10J0 | Insulin autoimmune syndrome without complication |
| Diabetes | C10E2 | Type 1 diabetes mellitus with neurological complications |
| Diabetes | C101. | Diabetes mellitus with ketoacidosis |
| Epilepsy | F254. | Partial epilepsy with impairment of consciousness |
| Epilepsy | F2552 | Somatosensory epilepsy |
| Epilepsy | F2545 | Complex partial epileptic seizure |
| Epilepsy | F2542 | Psychosensory epilepsy |
| Epilepsy | F2516 | Grand mal seizure |
| Epilepsy | F2550 | Jacksonian, focal or motor epilepsy |
| Epilepsy | F25y1 | Gelastic epilepsy |
| Epilepsy | F2553 | Visceral reflex epilepsy |
| Epilepsy | F250y | Other specified generalised nonconvulsive epilepsy |
| Epilepsy | F25H. | Generalised seizure |
| Epilepsy | F2541 | Psychomotor epilepsy |
| Epilepsy | F2501 | Pykno-epilepsy |
| Epilepsy | F2513 | Epileptic seizures - myoclonic |
| Epilepsy | F2540 | Temporal lobe epilepsy |
| Epilepsy | F255. | Partial epilepsy without impairment of consciousness |
| Epilepsy | F250. | Generalised nonconvulsive epilepsy |
| Epilepsy | F251. | Generalised convulsive epilepsy |
| Epilepsy | F256z | Infantile spasms NOS |
| Epilepsy | F25D. | Menstrual epilepsy |
| Epilepsy | F2555 | Unilateral epilepsy |
| Epilepsy | F2502 | Epileptic seizures - atonic |
| Epilepsy | F25z. | Epilepsy NOS |
| Epilepsy | F254z | Partial epilepsy with impairment of consciousness NOS |
| Epilepsy | F2504 | Juvenile absence epilepsy |
| Epilepsy | F255y | Partial epilepsy without impairment of consciousness OS |
| Epilepsy | F25F. | Photosensitive epilepsy |
| Epilepsy | F25A. | Juvenile myoclonic epilepsy |
| Epilepsy | F2515 | Tonic-clonic epilepsy |
| Epilepsy | F2503 | Epileptic seizures - akinetic |
| Epilepsy | F2500 | Petit mal (minor) epilepsy |
| Epilepsy | F2561 | Salaam attacks |
| Epilepsy | F25y2 | Locl-rlt(foc)(part)idiop epilep&epilptic syn seiz locl onset |
| Epilepsy | F2512 | Epileptic seizures - clonic |
| Epilepsy | F259. | Early infant epileptic encephalopathy wth suppression bursts |
| Epilepsy | F2560 | Hypsarrhythmia |
| Epilepsy | F2556 | Simple partial epileptic seizure |
| Epilepsy | F25y5 | Panayiotopoulos syndrome |
| Epilepsy | F25B. | Alcohol-induced epilepsy |
| Epilepsy | F251z | Generalised convulsive epilepsy NOS |
| Epilepsy | F2543 | Limbic system epilepsy |
| Epilepsy | F25E. | Stress-induced epilepsy |
| Epilepsy | F255z | Partial epilepsy without impairment of consciousness NOS |
| Epilepsy | F25yz | Other forms of epilepsy NOS |
| Epilepsy | F2551 | Sensory induced epilepsy |
| Epilepsy | F25y3 | Complex partial status epilepticus |
| Epilepsy | F253. | Grand mal status |
| Epilepsy | F2510 | Grand mal (major) epilepsy |
| Epilepsy | F25y0 | Cursive (running) epilepsy |
| Epilepsy | F250z | Generalised nonconvulsive epilepsy NOS |
| Epilepsy | F257. | Kojevnikov's epilepsy |
| Epilepsy | F25C. | Drug-induced epilepsy |
| Epilepsy | F258. | Post-ictal state |
| Epilepsy | F2544 | Epileptic automatism |
| Epilepsy | F25X. | Status epilepticus, unspecified |
| Epilepsy | F256. | Infantile spasms |
| Epilepsy | F2511 | Neonatal myoclonic epilepsy |
| Epilepsy | F25y. | Other forms of epilepsy |
| Epilepsy | F2505 | Lennox-Gastaut syndrome |
| Epilepsy | F2514 | Epileptic seizures - tonic |
| Epilepsy | F251y | Other specified generalised convulsive epilepsy |
| Epilepsy | F25G. | Severe myoclonic epilepsy in infancy |
| Epilepsy | F252. | Petit mal status |
| Epilepsy | F25y4 | Benign Rolandic epilepsy |
| Epilepsy | F2554 | Visual reflex epilepsy |
| Haemorrhagic stroke | G618. | Intracerebral haemorrhage, multiple localized |
| Haemorrhagic stroke | G615. | Bulbar haemorrhage |
| Haemorrhagic stroke | G611. | Internal capsule haemorrhage |
| Haemorrhagic stroke | G61X1 | Right sided intracerebral haemorrhage, unspecified |
| Haemorrhagic stroke | G619. | Lobar cerebral haemorrhage |
| Haemorrhagic stroke | G61.. | Intracerebral haemorrhage |
| Haemorrhagic stroke | G61X. | Intracerebral haemorrhage in hemisphere, unspecified |
| Haemorrhagic stroke | G610. | Cortical haemorrhage |
| Haemorrhagic stroke | G61X0 | Left sided intracerebral haemorrhage, unspecified |
| Haemorrhagic stroke | G614. | Pontine haemorrhage |
| Haemorrhagic stroke | G600. | Ruptured berry aneurysm |
| Haemorrhagic stroke | G613. | Cerebellar haemorrhage |
| Haemorrhagic stroke | G612. | Basal nucleus haemorrhage |
| Haemorrhagic stroke | G616. | External capsule haemorrhage |
| Haemorrhagic stroke | G617. | Intracerebral haemorrhage, intraventricular |
| Haemorrhagic stroke | G61z. | Intracerebral haemorrhage NOS |
| Heart failure | G5802 | Decompensated cardiac failure |
| Heart failure | 1O1.. | Heart failure confirmed |
| Heart failure | G1yz1 | Rheumatic left ventricular failure |
| Heart failure | G580. | Congestive heart failure |
| Heart failure | G5810 | Acute left ventricular failure |
| Heart failure | G234. | Hyperten heart&renal dis+both(congestv)heart and renal fail |
| Heart failure | G5801 | Chronic congestive heart failure |
| Heart failure | G232. | Hypertensive heart&renal dis wth (congestive) heart failure |
| Heart failure | G582. | Acute heart failure |
| Heart failure | G5803 | Compensated cardiac failure |
| Heart failure | G581. | Left ventricular failure |
| Heart failure | G5800 | Acute congestive heart failure |
| Heart failure | G58z. | Heart failure NOS |
| Heart failure | G58.. | Heart failure |
| Heart failure | G21z1 | Hypertensive heart disease NOS with CCF |
| Hypertension | G21z1 | Hypertensive heart disease NOS with CCF |
| Heart failure | G24z. | Secondary hypertension NOS |
| Heart failure | G230. | Malignant hypertensive heart and renal disease |
| Heart failure | G24z0 | Secondary renovascular hypertension NOS |
| Heart failure | G220. | Malignant hypertensive renal disease |
| Heart failure | G2101 | Malignant hypertensive heart disease with CCF |
| Heart failure | G26.. | Severe hypertension (Nat Inst for Health Clinical Ex 2011) |
| Heart failure | G2y.. | Other specified hypertensive disease |
| Heart failure | G24z1 | Hypertension secondary to drug |
| Heart failure | G28.. | Stage 2 hypertension (NICE - Nat Ins for Hth Clin Excl 2011) |
| Heart failure | G20z. | Essential hypertension NOS |
| Heart failure | G2110 | Benign hypertensive heart disease without CCF |
| Heart failure | G20.. | Essential hypertension |
| Heart failure | G244. | Hypertension secondary to endocrine disorders |
| Heart failure | G2z.. | Hypertensive disease NOS |
| Heart failure | G210. | Malignant hypertensive heart disease |
| Heart failure | G222. | Hypertensive renal disease with renal failure |
| Heart failure | G201. | Benign essential hypertension |
| Heart failure | G234. | Hyperten heart&renal dis+both(congestv)heart and renal fail |
| Heart failure | G24.. | Secondary hypertension |
| Heart failure | G2410 | Secondary benign renovascular hypertension |
| Heart failure | G232. | Hypertensive heart&renal dis wth (congestive) heart failure |
| Heart failure | G211. | Benign hypertensive heart disease |
| Heart failure | G23.. | Hypertensive heart and renal disease |
| Heart failure | G23z. | Hypertensive heart and renal disease NOS |
| Heart failure | 14A2. | H/O: hypertension |
| Heart failure | G250. | Stage 1 hyperten (NICE 2011) without evidnce end organ damge |
| Heart failure | G211z | Benign hypertensive heart disease NOS |
| Heart failure | G2... | Hypertensive disease |
| Heart failure | G22.. | Hypertensive renal disease |
| Heart failure | G210z | Malignant hypertensive heart disease NOS |
| Heart failure | G21.. | Hypertensive heart disease |
| Heart failure | G22z. | Hypertensive renal disease NOS |
| Heart failure | G240z | Secondary malignant hypertension NOS |
| Heart failure | G24zz | Secondary hypertension NOS |
| Heart failure | G221. | Benign hypertensive renal disease |
| Heart failure | G241z | Secondary benign hypertension NOS |
| Heart failure | G25.. | Stage 1 hypertension (NICE - Nat Ins for Hth Clin Excl 2011) |
| Heart failure | G2111 | Benign hypertensive heart disease with CCF |
| Heart failure | G27.. | Hypertension resistant to drug therapy |
| Heart failure | G240. | Secondary malignant hypertension |
| Heart failure | G233. | Hypertensive heart and renal disease with renal failure |
| Heart failure | G251. | Stage 1 hyperten (NICE 2011) with evidnce end organ damge |
| Heart failure | G241. | Secondary benign hypertension |
| Heart failure | G2400 | Secondary malignant renovascular hypertension |
| Heart failure | G202. | Systolic hypertension |
| Heart failure | G21z. | Hypertensive heart disease NOS |
| Heart failure | G21z0 | Hypertensive heart disease NOS without CCF |
| Heart failure | G231. | Benign hypertensive heart and renal disease |
| Heart failure | G200. | Malignant essential hypertension |
| Heart failure | G2100 | Malignant hypertensive heart disease without CCF |
| Heart failure | G21zz | Hypertensive heart disease NOS |
| Heart failure | G203. | Diastolic hypertension |
| Ischaemic heart disease | G3z.. | Ischaemic heart disease NOS |
| Ischaemic heart disease | G33z3 | Angina on effort |
| Ischaemic heart disease | G31.. | Other acute and subacute ischaemic heart disease |
| Ischaemic heart disease | G382. | Postoperative transmural myocardial infarction other sites |
| Ischaemic heart disease | G383. | Postoperative transmural myocardial infarction unspec site |
| Ischaemic heart disease | G34y1 | Chronic myocardial ischaemia |
| Ischaemic heart disease | G33z4 | Ischaemic chest pain |
| Ischaemic heart disease | G33z0 | Status anginosus |
| Ischaemic heart disease | G31y0 | Acute coronary insufficiency |
| Ischaemic heart disease | G330. | Angina decubitus |
| Ischaemic heart disease | G312. | Coronary thrombosis not resulting in myocardial infarction |
| Ischaemic heart disease | G33zz | Angina pectoris NOS |
| Ischaemic heart disease | G33z. | Angina pectoris NOS |
| Ischaemic heart disease | G363. | Ruptur cardiac wall w'out haemopericard/cur comp fol ac MI |
| Ischaemic heart disease | G34z0 | Asymptomatic coronary heart disease |
| Ischaemic heart disease | G33z7 | Stable angina |
| Ischaemic heart disease | G302. | Acute inferolateral infarction |
| Ischaemic heart disease | G3010 | Acute anteroapical infarction |
| Ischaemic heart disease | G31y1 | Microinfarction of heart |
| Ischaemic heart disease | G36.. | Certain current complication follow acute myocardial infarct |
| Ischaemic heart disease | G35.. | Subsequent myocardial infarction |
| Ischaemic heart disease | G34yz | Other specified chronic ischaemic heart disease NOS |
| Ischaemic heart disease | G30y2 | Acute septal infarction |
| Ischaemic heart disease | G31y. | Other acute and subacute ischaemic heart disease |
| Ischaemic heart disease | G301. | Other specified anterior myocardial infarction |
| Ischaemic heart disease | G33z2 | Syncope anginosa |
| Ischaemic heart disease | G32.. | Old myocardial infarction |
| Ischaemic heart disease | G3115 | Acute coronary syndrome |
| Ischaemic heart disease | G34z. | Other chronic ischaemic heart disease NOS |
| Ischaemic heart disease | G364. | Ruptur chordae tendinae/curr comp fol acute myocard infarct |
| Ischaemic heart disease | G34.. | Other chronic ischaemic heart disease |
| Ischaemic heart disease | G30y1 | Acute papillary muscle infarction |
| Ischaemic heart disease | G306. | True posterior myocardial infarction |
| Ischaemic heart disease | G35X. | Subsequent myocardial infarction of unspecified site |
| Ischaemic heart disease | G308. | Inferior myocardial infarction NOS |
| Ischaemic heart disease | G307. | Acute subendocardial infarction |
| Ischaemic heart disease | G38.. | Postoperative myocardial infarction |
| Ischaemic heart disease | G3114 | Worsening angina |
| Ischaemic heart disease | G3400 | Single coronary vessel disease |
| Ischaemic heart disease | G304. | Posterior myocardial infarction NOS |
| Ischaemic heart disease | G38z. | Postoperative myocardial infarction, unspecified |
| Ischaemic heart disease | G30B. | Acute posterolateral myocardial infarction |
| Ischaemic heart disease | G330z | Angina decubitus NOS |
| Ischaemic heart disease | G303. | Acute inferoposterior infarction |
| Ischaemic heart disease | G360. | Haemopericardium/current comp folow acut myocard infarct |
| Ischaemic heart disease | G301z | Anterior myocardial infarction NOS |
| Ischaemic heart disease | G3071 | Acute non-ST segment elevation myocardial infarction |
| Ischaemic heart disease | G30y0 | Acute atrial infarction |
| Ischaemic heart disease | G3113 | Refractory angina |
| Ischaemic heart disease | G311z | Preinfarction syndrome NOS |
| Ischaemic heart disease | G3011 | Acute anteroseptal infarction |
| Ischaemic heart disease | G384. | Postoperative subendocardial myocardial infarction |
| Ischaemic heart disease | G3300 | Nocturnal angina |
| Ischaemic heart disease | G30yz | Other acute myocardial infarction NOS |
| Ischaemic heart disease | G34y. | Other specified chronic ischaemic heart disease |
| Ischaemic heart disease | G3401 | Double coronary vessel disease |
| Ischaemic heart disease | G33z1 | Stenocardia |
| Ischaemic heart disease | G31yz | Other acute and subacute ischaemic heart disease NOS |
| Ischaemic heart disease | G305. | Lateral myocardial infarction NOS |
| Ischaemic heart disease | G30z. | Acute myocardial infarction NOS |
| Ischaemic heart disease | G381. | Postoperative transmural myocardial infarction inferior wall |
| Ischaemic heart disease | G361. | Atrial septal defect/curr comp folow acut myocardal infarct |
| Ischaemic heart disease | G342. | Atherosclerotic cardiovascular disease |
| Ischaemic heart disease | G31y3 | Transient myocardial ischaemia |
| Ischaemic heart disease | G3111 | Unstable angina |
| Ischaemic heart disease | G30.. | Acute myocardial infarction |
| Ischaemic heart disease | G3070 | Acute non-Q wave infarction |
| Ischaemic heart disease | G30X0 | Acute ST segment elevation myocardial infarction |
| Ischaemic heart disease | G34y0 | Chronic coronary insufficiency |
| Ischaemic heart disease | G310. | Postmyocardial infarction syndrome |
| Ischaemic heart disease | G331. | Prinzmetal's angina |
| Ischaemic heart disease | G344. | Silent myocardial ischaemia |
| Ischaemic heart disease | G33.. | Angina pectoris |
| Ischaemic heart disease | G343. | Ischaemic cardiomyopathy |
| Ischaemic heart disease | G33z5 | Post infarct angina |
| Ischaemic heart disease | G311. | Preinfarction syndrome |
| Ischaemic heart disease | G351. | Subsequent myocardial infarction of inferior wall |
| Ischaemic heart disease | G31y2 | Subendocardial ischaemia |
| Ischaemic heart disease | G300. | Acute anterolateral infarction |
| Ischaemic heart disease | G3112 | Angina at rest |
| Ischaemic heart disease | G365. | Rupture papillary muscle/curr comp fol acute myocard infarct |
| Ischaemic heart disease | G353. | Subsequent myocardial infarction of other sites |
| Ischaemic heart disease | G332. | Coronary artery spasm |
| Ischaemic heart disease | G30y. | Other acute myocardial infarction |
| Ischaemic heart disease | G362. | Ventric septal defect/curr comp fol acut myocardal infarctn |
| Ischaemic heart disease | G3y.. | Other specified ischaemic heart disease |
| Ischaemic stroke | G65zz | Transient cerebral ischaemia NOS |
| Ischaemic stroke | G65.. | Transient cerebral ischaemia |
| Ischaemic stroke | G64z. | Cerebral infarction NOS |
| Ischaemic stroke | G66.. | Stroke and cerebrovascular accident unspecified |
| Ischaemic stroke | G64z2 | Left sided cerebral infarction |
| Ischaemic stroke | G641. | Cerebral embolism |
| Ischaemic stroke | G6410 | Cerebral infarction due to embolism of cerebral arteries |
| Ischaemic stroke | G64.. | Cerebral arterial occlusion |
| Ischaemic stroke | G664. | Cerebellar stroke syndrome |
| Ischaemic stroke | G64z4 | Infarction of basal ganglia |
| Ischaemic stroke | G667. | Left sided CVA |
| Ischaemic stroke | G666. | Pure sensory lacunar syndrome |
| Ischaemic stroke | G661. | Anterior cerebral artery syndrome |
| Ischaemic stroke | G668. | Right sided CVA |
| Ischaemic stroke | G64z0 | Brainstem infarction |
| Ischaemic stroke | 14A7. | H/O: CVA/stroke |
| Ischaemic stroke | G640. | Cerebral thrombosis |
| Ischaemic stroke | G64z3 | Right sided cerebral infarction |
| Ischaemic stroke | G662. | Posterior cerebral artery syndrome |
| Ischaemic stroke | G6400 | Cerebral infarction due to thrombosis of cerebral arteries |
| Ischaemic stroke | G663. | Brain stem stroke syndrome |
| Ischaemic stroke | G65z1 | Intermittent cerebral ischaemia |
| Ischaemic stroke | G665. | Pure motor lacunar syndrome |
| Liver disease | J61yz | Other non-alcoholic chronic liver disease NOS |
| Liver disease | J615B | Toxic portal cirrhosis |
| Liver disease | J6156 | Capsular portal cirrhosis |
| Liver disease | J6150 | Unilobular portal cirrhosis |
| Liver disease | J617. | Alcoholic hepatitis |
| Liver disease | J6130 | Alcoholic hepatic failure |
| Liver disease | J615z | Non-alcoholic cirrhosis NOS |
| Liver disease | J615C | Xanthomatous portal cirrhosis |
| Liver disease | G8522 | Oesophageal varices in cirrhosis of the liver |
| Liver disease | J61y6 | Hepatic fibrosis with hepatic sclerosis |
| Liver disease | J612. | Alcoholic cirrhosis of liver |
| Liver disease | J61y. | Other non-alcoholic chronic liver disease |
| Liver disease | J614. | Chronic hepatitis |
| Liver disease | J6141 | Chronic active hepatitis |
| Liver disease | J6155 | Hypertrophic portal cirrhosis |
| Liver disease | J6158 | Juvenile portal cirrhosis |
| Liver disease | J6154 | Fatty portal cirrhosis |
| Liver disease | J61z. | Chronic liver disease NOS |
| Liver disease | J61y1 | Non-alcoholic fatty liver |
| Liver disease | J615y | Portal cirrhosis unspecified |
| Liver disease | J616. | Biliary cirrhosis |
| Liver disease | J6144 | Chronic lobular hepatitis |
| Liver disease | J6151 | Multilobular portal cirrhosis |
| Liver disease | J616z | Biliary cirrhosis NOS |
| Liver disease | J6140 | Chronic persistent hepatitis |
| Liver disease | J61y0 | Chronic yellow liver atrophy |
| Liver disease | J611. | Acute alcoholic hepatitis |
| Liver disease | J615G | Zooparasitic portal cirrhosis |
| Liver disease | J6142 | Chronic aggressive hepatitis |
| Liver disease | J6170 | Chronic alcoholic hepatitis |
| Liver disease | J61y8 | Nonalcoholic steatohepatitis |
| Liver disease | J6153 | Diffuse nodular cirrhosis |
| Liver disease | J613. | Alcoholic liver damage unspecified |
| Liver disease | J6161 | Secondary biliary cirrhosis |
| Liver disease | J610. | Alcoholic fatty liver |
| Liver disease | J615A | Pipe-stem portal cirrhosis |
| Liver disease | J6143 | Recurrent hepatitis |
| Liver disease | J615. | Cirrhosis - non alcoholic |
| Liver disease | J6356 | Toxic liver disease with fibrosis and cirrhosis of liver |
| Liver disease | J615D | Bacterial portal cirrhosis |
| Liver disease | J6162 | Biliary cirrhosis of children |
| Liver disease | C3104 | Glycogenosis with hepatic cirrhosis |
| Liver disease | J61y2 | Hepatosplenomegaly |
| Liver disease | J61y4 | Hepatic fibrosis |
| Liver disease | J615E | Cardituberculous cirrhosis |
| Liver disease | J615F | Syphilitic portal cirrhosis |
| Liver disease | J61y7 | Steatosis of liver |
| Liver disease | Jyu71 | [X]Other and unspecified cirrhosis of liver |
| Liver disease | J6120 | Alcoholic fibrosis and sclerosis of liver |
| Liver disease | J6160 | Primary biliary cirrhosis |
| Liver disease | J6157 | Cardiac portal cirrhosis |
| Liver disease | J614y | Chronic hepatitis unspecified |
| Liver disease | J6152 | Mixed portal cirrhosis |
| Liver disease | J61.. | Cirrhosis and chronic liver disease |
| Liver disease | J61y9 | Fatty change of liver |
| Liver disease | G8523 | Oesophageal varices in alcoholic cirrhosis of the liver |
| Liver disease | J614z | Chronic hepatitis NOS |
| Liver disease | J6159 | Pigmentary portal cirrhosis |
| Liver disease | J61y3 | Portal fibrosis without cirrhosis |
| Liver disease | J615H | Infectious cirrhosis NOS |
| Liver disease | J61y5 | Hepatic sclerosis |
| Respiratory disease | H3y.. | Other specified chronic obstructive airways disease |
| Respiratory disease | H32yz | Other emphysema NOS |
| Respiratory disease | H3... | Chronic obstructive pulmonary disease |
| Respiratory disease | H3200 | Segmental bullous emphysema |
| Respiratory disease | H320. | Chronic bullous emphysema |
| Respiratory disease | H3z.. | Chronic obstructive airways disease NOS |
| Respiratory disease | H37.. | Moderate chronic obstructive pulmonary disease |
| Respiratory disease | H32y2 | MacLeod's unilateral emphysema |
| Respiratory disease | H3121 | Emphysematous bronchitis |
| Respiratory disease | H3310 | Intrinsic asthma without status asthmaticus |
| Respiratory disease | H32y. | Other emphysema |
| Respiratory disease | H32y0 | Acute vesicular emphysema |
| Respiratory disease | H32z. | Emphysema NOS |
| Respiratory disease | H321. | Panlobular emphysema |
| Respiratory disease | H32.. | Emphysema |
| Respiratory disease | H333. | Acute exacerbation of asthma |
| Respiratory disease | H332. | Mixed asthma |
| Respiratory disease | H3202 | Giant bullous emphysema |
| Respiratory disease | H3201 | Zonal bullous emphysema |
| Respiratory disease | H300. | Tracheobronchitis NOS |
| Respiratory disease | H335. | Chronic asthma with fixed airflow obstruction |
| Respiratory disease | H32y1 | Atrophic (senile) emphysema |
| Respiratory disease | H3300 | Extrinsic asthma without status asthmaticus |
| Respiratory disease | H3122 | Acute exacerbation of chronic obstructive airways disease |
| Respiratory disease | H330. | Extrinsic (atopic) asthma |
| Respiratory disease | H331z | Intrinsic asthma NOS |
| Respiratory disease | H334. | Brittle asthma |
| Respiratory disease | H33zz | Asthma NOS |
| Respiratory disease | H330z | Extrinsic asthma NOS |
| Respiratory disease | H33z1 | Asthma attack |
| Respiratory disease | H3A.. | End stage chronic obstructive airways disease |
| Respiratory disease | H3203 | Bullous emphysema with collapse |
| Respiratory disease | H38.. | Severe chronic obstructive pulmonary disease |
| Respiratory disease | H3B.. | Asthma-chronic obstructive pulmonary disease overlap syndrom |
| Respiratory disease | H39.. | Very severe chronic obstructive pulmonary disease |
| Respiratory disease | H33.. | Asthma |
| Respiratory disease | H36.. | Mild chronic obstructive pulmonary disease |
| Respiratory disease | H3y1. | Chron obstruct pulmonary dis wth acute exacerbation, unspec |
| Respiratory disease | H322. | Centrilobular emphysema |
| Respiratory disease | H33z0 | Status asthmaticus NOS |
| Respiratory disease | H3y0. | Chronic obstruct pulmonary dis with acute lower resp infectn |
| Respiratory disease | H331. | Intrinsic asthma |
| Respiratory disease | H320z | Chronic bullous emphysema NOS |
| Respiratory disease | H3311 | Intrinsic asthma with status asthmaticus |
| Respiratory disease | H33z. | Asthma unspecified |
| Respiratory disease | H3301 | Extrinsic asthma with status asthmaticus |
| Respiratory disease | H33z2 | Late-onset asthma |
| Thrombo embolism | G7423 | Embolism and thrombosis of an arm artery NOS |
| Thrombo embolism | G7421 | Embolism and thrombosis of the radial artery |
| Thrombo embolism | G7422 | Embolism and thrombosis of the ulnar artery |
| Thrombo embolism | G7424 | Embolism and thrombosis of the femoral artery |
| Thrombo embolism | G7427 | Embolism and thrombosis of the dorsalis pedis artery |
| Thrombo embolism | G74y7 | Embolism and thrombosis of the axillary artery |
| Thrombo embolism | G74yz | Embolism and thrombosis of other arteries NOS |
| Thrombo embolism | G74y8 | Embolism and thrombosis of the coeliac artery |
| Thrombo embolism | G7426 | Embolism and thrombosis of the anterior tibial artery |
| Thrombo embolism | G74y1 | Embolism and/or thrombosis of the internal iliac artery |
| Thrombo embolism | G7420 | Embolism and thrombosis of the brachial artery |
| Thrombo embolism | G740. | Embolism and thrombosis of the abdominal aorta |
| Thrombo embolism | G74y3 | Embolism and thrombosis of the iliac artery unspecified |
| Thrombo embolism | G7425 | Embolism and thrombosis of the popliteal artery |
| Thrombo embolism | G74y0 | Embolism and/or thrombosis of the common iliac artery |
| Thrombo embolism | G74.. | Arterial embolism and thrombosis |
| Thrombo embolism | G741. | Embolism and thrombosis of the thoracic aorta |
| Thrombo embolism | G74z. | Arterial embolism and thrombosis NOS |
| Thrombo embolism | G74y. | Embolism and thrombosis of other specified artery |
| Thrombo embolism | G7429 | Embolism and thrombosis of a leg artery NOS |
| Thrombo embolism | G74y6 | Embolism and thrombosis of the splenic artery |
| Thrombo embolism | G742B | Post radiological embolism of lower limb artery |
| Thrombo embolism | G742A | Post radiological embolism of upper limb artery |
| Thrombo embolism | G74y9 | Embolism and thrombosis of the hepatic artery |
| Thrombo embolism | G742z | Peripheral arterial embolism and thrombosis NOS |
| Thrombo embolism | G74y2 | Embolism and/or thrombosis of the external iliac artery |
| Thrombo embolism | G743. | Embolism and thrombosis of other and unspec parts aorta |
| Thrombo embolism | G74y5 | Embolism and thrombosis of the subclavian artery |
| Thrombo embolism | G7428 | Embolism and thrombosis of the posterior tibial artery |
| Thrombo embolism | G742. | Embolism and thrombosis of an arm or leg artery |
| Valvular AF | 79100 | Allograft replacement of mitral valve |
| Valvular AF | 79182 | Annuloplasty of valve of heart NEC |
| Valvular AF | G1... | Chronic rheumatic heart disease |
| Valvular AF | 7917. | Closed incision of heart valve |
| Valvular AF | 79170 | Closed mitral valvotomy |
| Valvular AF | G112. | Mitral stenosis with insufficiency |
| Valvular AF | P65.. | Congenital mitral stenosis |
| Valvular AF | G13.. | Diseases of mitral and aortic valves |
| Valvular AF | G130. | Mitral and aortic stenosis |
| Valvular AF | G110. | Mitral stenosis |
| Valvular AF | G131. | Mitral stenosis and aortic insufficiency |
| Valvular AF | 7917z | Closed incision of valve of heart NOS |
| Valvular AF | G11.. | Mitral valve diseases |
| Valvular AF | G13y. | Multiple mitral and aortic valve involvement |
| Valvular AF | G113. | Nonrheumatic mitral valve stenosis |
| Valvular AF | 79160 | Open mitral valvotomy |
| Valvular AF | 791B2 | Operations on mitral subvalvar apparatus |
| Valvular AF | Gyu10 | [X]Other mitral valve diseases |
| Valvular AF | G1yzz | Other rheumatic heart disease NOS |
| Valvular AF | 79190 | Percutaneous transluminal mitral valvotomy |
| Valvular AF | 79102 | Prosthetic replacement of mitral valve |
| Valvular AF | 79103 | Replacement of mitral valve NEC |
| Valvular AF | G111. | Rheumatic mitral insufficiency |
| Valvular AF | 79101 | Xenograft replacement of mitral valve |
| Valvular AF | 7918y | Other specified other open operation on valve of heart |
