# Supplementary figures and images for: An observational study of international normalized ratio control according to NICE criteria in patients with non-valvular atrial fibrillation: the SAIL Warfarin Out of Range Descriptors Study (SWORDS)
Source: Eur Heart J Cardiovasc Pharmacother. 2019 Nov 27;7(1):40–9. doi: 10.1093/ehjcvp/pvz071 (PMC7811400; doi:10.1093/ehjcvp/pvz071)

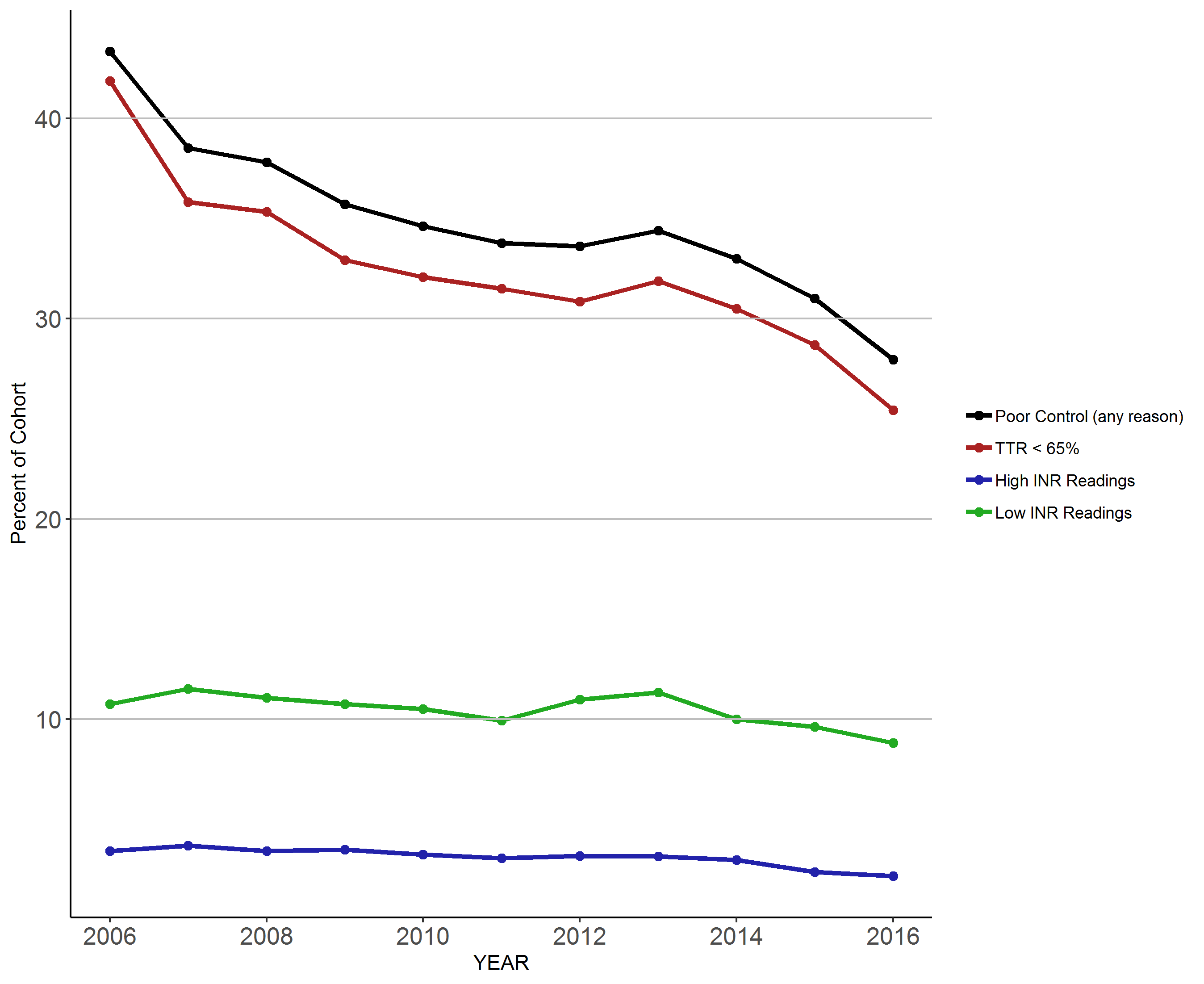

Supplement: pvz071_Supplementary_Data [file pvz071_supplementary_data.zip › SUPfig1_swords.tiff]

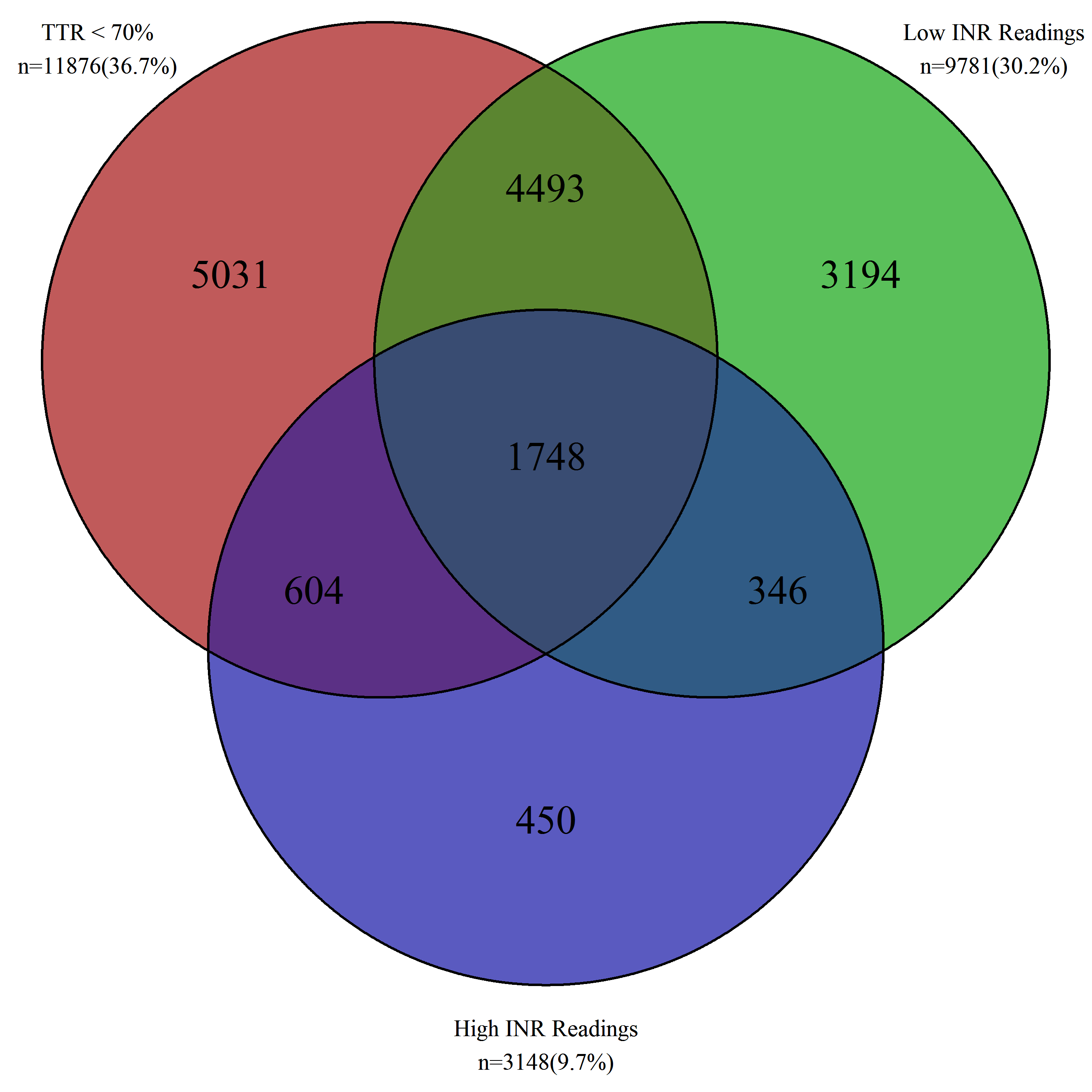

Supplement: pvz071_Supplementary_Data [file pvz071_supplementary_data.zip › supfig2_swords.tiff]
